# Association between dietary mineral intake and new onset diabetes/pre-diabetes after chronic pancreatitis

**DOI:** 10.3389/fnut.2024.1461468

**Published:** 2025-01-07

**Authors:** Bingqing Li, Shan Guo, Wenlu Zong, Yuning Chu, Qi Zhang, Xiaoyan Yin, Tao Mao, Xiaoyu Li

**Affiliations:** ^1^Department of Gastroenterology, The Affiliated Hospital to Qingdao University, Qingdao, China

**Keywords:** mineral intake, nutrition, chronic pancreatitis, pancreatogenic diabetes, trace elements

## Abstract

**Background and aims:**

As the main type of pancreatic diabetes, patients with new diabetes after chronic pancreatitis are often difficult to manage and have poor prognosis. This study aimed to figure out the association between dietary mineral intake and glucose metabolism with chronic pancreatitis.

**Method:**

The study included 114 patients with chronic pancreatitis, who were grouped based on the sequence of onset for chronic pancreatitis and diabetes: normoglycaemia after chronic pancreatitis (NCP), type 2 diabetes (T2DM), and new-onset diabetes or pre-diabetes after chronic pancreatitis (NODCP). The habitual intake of 10 minerals (calcium, chlorine, iodine, iron, magnesium, phosphorus, potassium, selenium, sodium, and zinc) was assessed using a dietary health questionnaire. The differences in mineral intake between the groups were compared, and the relationship between mineral intake and key glucose metabolism markers, including fasting plasma glucose (FPG), glycated hemoglobin (HbA1c), and fasting insulin, was analyzed using regression models.

**Results:**

Compared with normal glycaemic status after chronic pancreatitis, the intake of iron and phosphorus in patients with new diabetes/pre-diabetes after chronic pancreatitis (NODCP) has changed significantly. In the NODCP group, FPG levels were significantly negatively correlated with magnesium intake, while HbA1c levels were significantly negatively correlated with average phosphorus intake. In addition, there is a correlation between fasting insulin and average magnesium intake in the NODCP group. No correlation was found between the intake of other minerals and glucose metabolism in chronic pancreatitis.

**Conclusion:**

The intake of minerals in the diet affects the glycaemic status after chronic pancreatitis. It is necessary to further explore the possible causal relationship and mechanism between mineral intake and diabetes after chronic pancreatitis, so as to provide evidence for nutritional intervention of high-risk patients.

## 1 Introduction

Diabetes mellitus (DM) is a group of metabolic disorders caused by absolute or relative insufficiency of insulin secretion and (or) insulin utilization disorders, which is mainly characterized by hyperglycemia (1). Most diabetes is a primary pancreatic endocrine disease. The most prevalent form is type 2 diabetes. However, about 4–5% of diabetes is developed from exocrine diseases of the pancreas. This type of diabetes is described as pancreatogenous diabetes or type 3c diabetes. It is generally believed that 79% of type 3c diabetes is secondary to chronic pancreatitis (2, 3). Chronic pancreatitis is a multifactorial, fibroinflammatory syndrome in which repetitive episodes of pancreatic inflammation lead to extensive fibrotic tissue replacement, resulting in chronic pain, exocrine and endocrine pancreatic insufficiency, reduced quality of life, and a shorter life expectancy (4). The prevalence of chronic pancreatitis is 13.5–52.4 cases per 100,000, and an incidence of five new cases per 100,000 inhabitants per year (5). The etiology and risk factors of chronic pancreatitis include excessive alcohol consumption, hyperlipidemia, nicotine consumption, pancreatic duct obstruction, immunological factors, hereditary factors, and so on (6).

For patients with CP, the prevalence of diabetes secondary to CP is about 25–80%, and the risk gradually increases with the prolongation of the duration of disease, and tends to stabilize after about 36 months (7). Once the disease is established, there are many differences between type 2 diabetes and type 3c diabetes. Type 2 diabetes is characterized initially by impaired insulin sensitivity and subsequently by an inadequate compensatory insulin response. Different from type 2 diabetes, the earliest pathogenesis of type 3c diabetes secondary to chronic pancreatitis is insufficient insulin secretion. Mild insulin deficiency exists even before the development of type 3c diabetes. As chronic pancreatitis progresses, the extensive fibrosis of the exocrine pancreas slowly destroys the pancreatic islet tissue (8). Additionally, the increase in pro-inflammatory cytokines, like TNF-*α*, can activate a number of intracellular signaling molecules, such as JNK and IKKβ that are critical components of the inflammatory signaling system, leading to impaired insulin action (9, 10). Therefore, diabetes secondary to CP has more unstable blood glucose and poor prognosis. Given the particularity of diabetes secondary to CP, it is unreasonable to apply the management method of type 2 diabetes generally (11). Nutritional therapy has been recognized as an indispensable part of diabetes treatment. Minerals play a crucial role in glucose metabolism, affecting blood sugar regulation and insulin effectiveness (12). Additionally, minerals significantly influence exocrine pancreatic diseases, such as chronic pancreatitis and pancreatic cancer. Magnesium is an important mineral closely related to glucose metabolism. Research indicates that magnesium deficiency is directly linked to insulin resistance, leading to difficulties in blood sugar control. A study found that magnesium supplementation significantly lowered fasting blood glucose and HbA1c levels in patients with diabetes. The bioavailability of magnesium in diabetic patients is often low, partly due to interactions with medications and other dietary components (13). Zinc plays a critical role in the synthesis, storage, and release of insulin. A deficiency in zinc may lead to decreased insulin secretion, thus affecting blood sugar regulation. Studies show that zinc can enhance pancreatic *β*-cell function, improving insulin levels and metabolic status in patients with diabetes. Research indicated that zinc supplementation can increase insulin sensitivity and lower markers of insulin resistance in diabetic patients (14). Selenium is an essential trace mineral that also plays a key role in glucose metabolism. Its antioxidant properties help protect pancreatic cells from oxidative stress, supporting normal insulin secretion. A study found that selenium supplementation could improve insulin sensitivity and lower blood glucose levels in patients with diabetes. High-protein diets can enhance selenium absorption, while high levels of certain minerals, like iron, may compete with selenium for absorption (15). Phosphorus is crucial for glucose metabolism, participating in energy production and cell signaling. Phosphorylation reactions are vital in the insulin signaling pathway, facilitating glucose entry into cells. Research indicates that inadequate phosphorus intake may disrupt insulin action, affecting blood sugar levels (16). Iron is another essential mineral involved in oxygen transport and energy metabolism. Research indicates that iron directly impacts glucose metabolism, especially in patients with diabetes, where low iron levels correlate with decreased insulin sensitivity. However, excess iron intake and disruptions in iron homeostasis can negatively affect glucose metabolism. Excessive iron can induce oxidative stress, causing cellular damage, particularly impacting the function of pancreatic *β*-cells and reducing insulin secretion. Studies have shown that iron accumulation is closely linked to insulin resistance and the development of type 2 diabetes. Research has shown that iron overload can activate ferroptosis-related signaling pathways, leading to *β*-cell apoptosis and exacerbating insulin resistance and hyperglycemia (17). Calcium influences the electrophysiological properties of pancreatic cells and intracellular signaling pathways, promoting insulin release. Overall, the importance of minerals in glucose metabolism cannot be overlooked, especially in managing diabetes and exocrine pancreatic diseases. Paying attention to mineral intake in daily diets is an effective way to maintain health and optimize glucose metabolism (18).

Researchers have confirmed associations of habitual mineral intake with pre-diabetes/diabetes after acute pancreatitis. They found that after the onset of acute pancreatitis, patients with normal blood glucose and patients with pre-diabetes/diabetes have different mineral intake habits (19). In view of this, we try to study the correlation between mineral intake and pre-diabetes and diabetes after chronic pancreatitis.

This study analyzed the differences in mineral intake among three groups: new-onset pre-diabetes/diabetes after chronic pancreatitis (NODCP), pre-existing prediabetes/ type 2 diabetes (T2DM), normal blood glucose after CP (NCP) and the impact of mineral intake on glucose metabolism markers in patients within each group.

## 2 Materials and methods

### 2.1 Study populations

**Figure 1:**
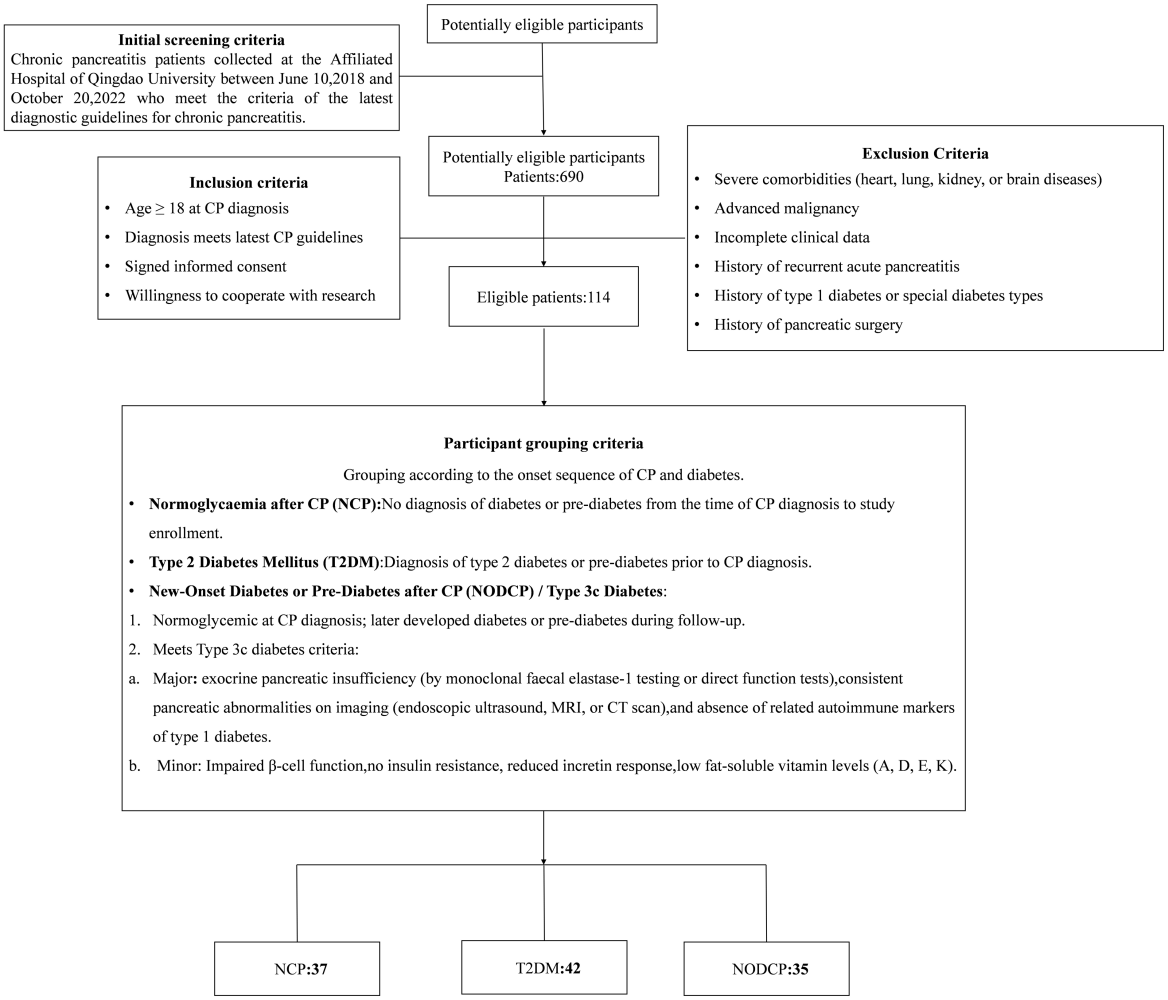
The flowchart for patient screening and enrollment.

This study examined 690 patients diagnosed with chronic pancreatitis (CP) who received treatment at the Affiliated Hospital of Qingdao University from June 2018 to October 2022. Diagnosis was based on the criteria set forth in the latest diagnostic guidelines (20) (Table 1). After applying specific inclusion and exclusion criteria, a total of 114 patients were included in the study. According to the sequence of onset for CP and diabetes, participants were divided into three groups: 37 cases of Normoglycaemia after CP (NCP), 42 cases of Type 2 Diabetes Mellitus (T2DM), and 35 cases of New-Onset Diabetes or Pre-Diabetes after CP (NODCP) / Type 3c Diabetes (Figure 1).

**Table 1:** Diagnostic criteria for chronic pancreatitis.

Diagnostic method	Clinical features and probable imaging criteria, or definitive imaging criteria alone.
Clinical features	Two or more of the following clinical features: repeated upper abdominal pain; abnormal serum or urine pancreatic enzyme concentrations (lipase or amylase activity 2–3 times above the upper limit of normal); abnormal exocrine function (stool elastase less than 200 μg/g).
Imaging criteria	Definitive imaging criteria according to Cambridge classification (Cambridge Grade 4).
Endoscopic retrograde cholangiopancreatography	Grade 3 plus at least one pseudocyst ≤10 mm, pancreatic duct stones, main duct strictures, or extension to neighboring organs.
Ultrasound	Grade 3 plus at least one of pancreatic duct stones, main duct obstruction, inflammatory mass > ×2, or splenic vein thrombosis.
CT or MRI	Grade 2 or Grade 3 plus at least one of pseudocyst ≥10 mm parenchymal calcifications, main duct calcifications, main duct obstruction, or main duct irregularities.

From the diagnosis of CP to this study, participants who did not meet the diagnostic criteria of diabetes or pre-diabetes were assigned to normoglycaemia after CP (NCP). Participants who had been diagnosed with type 2 diabetes or pre-diabetes before being diagnosed with CP were assigned to the diabetes group (T2DM). When diagnosing CP, the blood sugar and glycosylated hemoglobin levels were within the normal range. During follow-up, they met the diagnosis of diabetes or pre-diabetes. These patients were divided into new-onset diabetes or pre-diabetes after CP (NODCP), or 3c diabetes patients. In addition, they need to meeteet the following diagnostic criteria: exocrine pancreatic insufficiency (by monoclonal faecal elastase-1 testing or direct function tests), consistent pancreatic abnormalities on imaging (endoscopic ultrasound, MRI, or CT scan), and absence of related autoimmune markers of type 1 diabetes. Diagnostic criteria for diabetes are shown in Table 2.

**Table 2:** Diagnostic criteria for diabetes and pre-diabetes.

	Diagnostic test	Criteria
**Diabetes** (diagnosis can be made if any of the following criteria is met)	FPG	≥ 7.0 mmol/L
	75-g OGTT 2-h blood glucose	≥ 11.1 mmol/L
	HbA1c	5.6 mmol/L - 6.9 mmol/L
**Pre-diabetes** (diagnosis can be made if any of the following criteria is met)	FPG	5.6 mmol/L - 6.9 mmol/L
	75-g OGTT 2-h blood glucose	7.8 mmol/L - 11.0 mmol/L
	HbA1c	5.7–6.4%

### 2.2 Ethics statement

The study was conducted according to the guidelines in the Declaration of Helsinki. This study involves human participants and was approved by the Ethics Committee of Qingdao University Affiliated Hospital. NO.16 Jiangsu Road, Shinan District, Qingdao, Shandong Province, China. ID: QYFY WZLL 28588. All study participants provided written informed consent.

### 2.3 Evaluation of mineral intake

Based on the dietary habits of the local population, this study used a dietary health assessment questionnaire developed by the Chinese Nutrition Center to evaluate the habitual diet of participants. This questionnaire is available for open access (http://www.yyjy365.org/diet). This system is suitable for the dietary patterns of populations in inland and coastal areas of China. The questionnaire consists of two parts. The first part obtains basic information, including gender, age, and place of residence; The second part investigates the health status and intake of 136 common foods. Common foods are classified into the following categories: grains, potatoes, soy products, vegetables, algae, fresh fruits, nuts and seeds, livestock, poultry, dairy products, eggs, seafood such as fish and shrimp, fast food, sugar and desserts, beverages, seasonings, and others. Participants input the frequency of consuming common foods (such as twice a week, once a day, *et al*) and the average amount consumed per session (such as 100 g per session, 200 mL per session, *et al*) into the system based on their dietary habits. In addition, the system has placed a reference chart for the standard amount of food to help participants more accurately evaluate their dietary intake. The system calculates the average daily nutrient intake of participants based on the intake of different foods. After obtaining information, the system automatically generates the intake of 10 minerals, including calcium (mg), chlorine (mg), iodine (*μ*g), iron (mg), magnesium (mg), phosphorus (mg), potassium (mg), selenium (μ g), sodium (mg), and zinc (mg). Professional evaluation of questionnaire quality: multiple missing and identical options will be excluded. The survey questionnaire evaluated the habitual diet after diagnosis of chronic pancreatitis. Participants with significant changes in dietary habits will be excluded.

### 2.4 Evaluation of outcome indicators and covariates

Obtain fasting venous blood from participants, and measure fasting blood glucose, glycated hemoglobin, and fasting insulin to evaluate glucose metabolism. All study participants were certified by a phlebotomist to be fasting for at least 8 h before blood collection, after which venous blood samples were drawn. Fresh blood samples were analyzed by the Department of Laboratory Medicine at the Affiliated Hospital of Qingdao University. Hemoglobin A1c (HbA1c, mmol/mol) was measured using boronate affinity chromatography, fasting plasma glucose (FPG, mmol/L) was measured by enzymatic colorimetric assay, and fasting insulin (mU/L) was measured using a chemiluminescent sandwich immunoassay. A Homeostasis Model Assessment calculator (HOMA2), developed by Oxford University, was used to estimate HOMA- *β* indices as percentages of a normal reference population (version 2.2.4 Diabetes Trials Unit, University of Oxford, Oxford, UK). HOMA2 is a tool used to evaluate insulin resistance and *β*-cell function, widely applied in the management of diabetes. The HOMA2 model is based on measurements of fasting blood glucose and fasting insulin, providing critical physiological insights. HOMA - *β* is a main indicator of HOMA2. Its calculation formula is as follows.


$$\mathrm{HOMA}-\beta =\left(20\times \mathrm{Fasting Insulin}\right)/\left(FPG-3.5\right)\text{.}$$

It provides insight into how well the beta cells are functioning, which is crucial for diagnosing and managing diabetes. A low HOMA-*β* value indicates diminished *β*-cell function, which may lead to insufficient insulin secretion in diabetic patients (21). Fasting blood glucose refers to the blood sugar level measured after an 8-h fasting period. The normal range is 3.9–5.6 mmol/L, and a level of 7.0 mmol/L or higher can diagnose diabetes. Elevated fasting blood glucose indicates an increased risk of insulin resistance or diabetes. Fasting insulin is defined as the insulin level measured after an 8-h fast, with a normal range typically between 2 and 25 μU/mL (varies by laboratory). It assesses the pancreas’s insulin secretion capacity, and studies show that fasting insulin levels can aid in diagnosing insulin resistance (22). HbA1c reflects the average blood glucose levels over the past 2–3 months, with a normal range of <5.7%, and ≥ 6.5% indicating diabetes. It is useful for evaluating long-term blood glucose control, and research indicates that reductions in HbA1c levels are significantly associated with a lower risk of diabetes-related complications (23). While fasting blood glucose and fasting insulin provide snapshot measurements, the HOMA2 model integrates both to offer a comprehensive assessment of insulin resistance and *β*-cell function, helping physicians gain a fuller understanding of a patient’s metabolic status. Fasting blood glucose and HbA1c are commonly used for initial diabetes diagnosis and long-term monitoring, whereas fasting insulin, along with HOMA-IR and HOMA-*β*, is employed for in-depth metabolic evaluations to guide personalized treatment plans (24). Integrating these indicators allows for the assessment of how minerals affect glucose metabolism in patients. This approach enables the observation of minerals’ impact on insulin resistance and β-cell function in chronic pancreatitis patients, as well as their influence on short-term and long-term blood glucose control, thereby providing a more comprehensive and effective evaluation.

Basic information such as age, gender, smoking, and alcohol consumption, as well as disease characteristics such as disease course, medication, and symptoms, are all from the medical records of participants in our medical institution. Missing information is supplemented by contacting patients during the study. The total energy intake and the proportion of dietary fat intake were obtained from the dietary survey questionnaire filled out by participants.

### 2.5 Statistical analysis

All data analysis was conducted through IBM SPSS Statistics 26. One-way analysis of variance was used to investigate the differences in baseline characteristics between study groups (NODCP, T2DM, and NCP). Present results as mean ± standard deviation or frequency (percentage). Subsequently, non-parametric tests were used to analyze the differences in average mineral intake between groups, in order to investigate the impact of intake differences on different blood glucose levels after CP. Finally, multiple linear regression analysis was conducted within each study group to investigate the relationship between mineral intake and glucose metabolism indicators within different groups. Model 1 has not been adjusted; Model 2 adjusted daily energy intake; Model 3 adjusted daily energy intake and proportion of fat intake; Model 4 adjusted for age, gender, daily energy intake, and proportion of fat intake; Model 5 is based on age, gender, daily energy intake, proportion of fat intake, smoking status, daily alcohol intake, acute pancreatitis, and concurrent pancreatic exocrine dysfunction. The final result is expressed as R^2^, unstandardized B, *p*-value, and 95% confidence interval. In all analyses, *p* < 0.05 is considered statistically significant.

## 3 Results

A total of 114 patients were included in this study, including 37 participants in the NCP, 42 participants in T2DM, and 35 participants in NODCP. Table 3 presents the clinical characteristics related to the study cohort. There was no significant difference among the three groups in age, gender, average daily energy intake, proportion of dietary fat, BMI, course, history of biliary diseases, acute pancreatitis, alcohol and tobacco intake, and pancreatic exocrine insufficiency (PEI) (*p* > 0.05), but there was significant difference in glycosylated hemoglobin, fasting blood glucose, and diabetes medication (*p* < 0.001).

### 3.1 Differences in average mineral intake between groups in dietary minerals

With the NCP group as the control group, there is no significant difference in the intake of various mineral elements between the T2DM and the control group. The difference in average intake of magnesium (*p* = 0.011) and iron (*p* = 0.019) between the NODCP group and the control group was statistically significant, while the difference in intake of other elements was not significant Table 4.

**Table 3:** Characteristics of study participants.

Characteristic	Total	NODCP	T2DM	NCP	p
	*N* = 114	*n* = 37	*n* = 42	*n* = 35	
Age	66.5 (7.7)	65.6 (6.7)	66.3 (7.7)	67.9 (8.7)	0.422
Sex					0.612
Men	72 (63.2)	25 (67.6)	24 (57.1)	23 (65.7)	
Women	42 (36.8)	12 (32.4)	18 (42.9)	12 (34.3)	
Average daily energy intake (kcal)	1844 (391)	1889 (437)	1739 (316)	1924 (405)	0.084
Dietary fat percentage	29.7 (7.80)	31.8 (6.9)	29.2 (8.07)	28.2 (8.10)	0.136
BMI	22.0 (1.72)	21.9 (1.68)	22.3 (1.57)	21.7 (1.92)	0.274
Duration of CP (year)	9.34 (2.41)	9.31 (2.26)	8.81 (1.51)	10.0 (3.21)	0.096
Biliary diseases	33 (29)	11 (30)	13 (31)	9 (26)	0.730
Episodes of AP	29 (25)	8 (22)	11 (26)	10 (29)	0.829
Alcohol intake (g/d)	9.3 (14.9)	10.1 (15.5)	8.8 (15.0)	9.1 (14.4)	0.922
Tobacco intake (cigarette per day)	4.3 (10.0)	5.4 (10.9)	3.6 (9.2)	4.0 (10.1)	0.706
Pancreatic Exocrine Insufficiency	45	16 (43.2)	15 (35.7)	12 (34.3)	0.738
HbA1c (mmol/mol)	6.4 (1.5)	7.0 (2.0)	6.9 (1.5)	5.1 (1.6)	0.000
Fasting plasma glucose (mmol/L)	7.0 (2.6)	8.1 (2.4)	7.6 (3.1)	5.2 (1.8)	0.000
Medication for diabetes					0.000
None	55 (48)	11 (30)	9 (22)	35 (100)	
Oral hypoglycemic drugs	37 (33)	18 (49)	19 (45)	0 (0)	
Insulin	22 (19)	8 (21)	14 (33)	0 (0)	

**Table 4:** Differences in average mineral intake between groups in dietary minerals.

Mineral	NCP	T2DM-NCP		NODCP-NCP	
		T2DM	*p*	NODCP	*p*
Sodium	2.0 (1.6, 2.2)	2.2 (1.7,2.4)	0.053	2.0 (1.5,2.6)	0.300
Chlorine	3.2 (2.5, 3.5)	3.3 (2.6,3.8)	0.092	3.1 (2.4,4.0)	0.370
Calcium	830 (732,1,090)	736 (648,942)	0.069	992 (756,1,048)	0.133
Phosphorus	941 (673,1,265)	895 (651,1,106)	0.927	828 (675,1,164)	0.330
Potassium	3,670 (3,306,4,275)	3,747 (2,976,4,693)	0.927	3,410 (2,395,4,299)	0.078
Magnesium	366 (298,414)	365 (250,435)	0.220	300 (230,386)	**0.011**
Iron	14.4 (13.0,16.1)	14.6 (12.3,16.9)	0.164	17.3 (14.3,18.2)	**0.019**
Iodine	134 (120,159)	131 (109,174)	0.976	129 (102,145)	0.077
Zinc	14.4 (12.2,15.3)	13.9 (12.1,14.9)	0.334	14.1 (11.9,15.0)	0.161
Selenium	58 (43,66)	56 (40,67)	0.984	52 (38,59)	0.086

NODCP, new-onset pre-diabetes/diabetes after chronic pancreatitis; T2DM, pre-existing prediabetes/ type 2 diabetes; NCP, normal blood glucose after CP. CP, chronic pancreatitis. HbA1c, glycated haemoglobin. Data are presented as mean (standard deviation) or frequency (percentage). *p*-values were calculated from one way ANOVA. Significance was set at *p* < 0.05.

NODCP, new-onset pre-diabetes/diabetes after chronic pancreatitis; T2DM, pre-existing prediabetes/ type 2 diabetes; NCP, normal blood glucose after CP. CP, chronic pancreatitis. Data are presented as median (first Quartile, third Quartile). *p*-values were calculated from non parametric tests. Significance was set at *p* < 0.05. Bold represents statistical significance.

### 3.2 Association between mineral intake and FPG in the study group

There was no statistically significant correlation between the minerals and FPG in the NCP group. FPG levels in the NODCP group are associated with minerals. In all models, magnesium is significantly negatively correlated with FPG (*p* = 0.029 in Model 1, *p* = 0.007 in Model 2, *p* = 0.004 in Model 3, *p* = 0.008 in Model 4, and *p* = 0.033 in Model 4). In all models of the T2DM group, FPG levels were significantly negatively correlated with phosphorus (*p* = 0.036 in Model 1, p = 0.033 in Model 2, *p* = 0.037 in Model 3, *p* = 0.045 in Model 4, and *p* = 0.024 in Model 5) Table 5.

**Table 5:** Associations between mineral intake and fasting blood glucose in the research group.

Mineral	Model	NCP (*n* = 37)		NODCP (*n* = 35)		T2DM (*n* = 42)	
		*p*	*β* coefficient (95%CI)	*p*	*β* coefficient (95%CI)	*p*	*Β* coefficient (95%CI)
Sodium	1	0.261	−0.262 (−0.158 to 0.329)	0.892	−0.026 (−1.816 to 1.589)	0.619	−0.139 (−3.230 to 1.955)
	2	0.555	−0.108 (−0.756 to 0.414)	0.935	−0.015 (−1.681 to 1.551)	0.615	−0.142 (−3.274 to 1.971)
	3	0.254	−0.281 (−1.232 to 0.3433)	0.671	−0.08 (−2.004 to 1.313)	0.575	−0.167 (−3.536 to 2.004)
	4	0.745	−0.061 (−0.697 to 0.504)	0.935	0.018 (−1.832 to 1.984)	0.623	−0.150 (−3.525 to 2.150)
	5	0.490	−0.173 (−1.082 to 0.535)	0.640	0.132 (−1.965 to 3.096)	0.944	0.025 (−3.287 to 3.519)
chlorine	1	0.581	−0.097 (−0.455 to 0.259)	0.793	0.045 (−0.828 to 1.076)	0.401	0.203 (−0.754 to 1.831)
	2	0.252	−0.276 (−0.771 to 0.212)	0.671	0.074 (−0.765 to 1.174)	0.496	0.169 (−0.886 to 1.787)
	3	0.758	−0.058 (−0.443 to 0.326)	0.922	0.018 (−0.960 to 1.058)	0.494	0.173 (−0.901 to 1.821)
	4	0.489	−0.173 (−0.696 to 0.345)	0.596	0.105 (−0.817 to 1.398)	0.568	0.149 (−1.014 to 1.809)
	5	0.145	−0.697 (−1.689 to 0.277)	0.335	0.214 (−0.652 to 1.840)	0.973	0.010 (−1.658 to 1.712)
Calcium	1	0.395	−0.243 (−0.001 to 0.001)	0.834	0.044 (−0.001 to 0.002)	0.277	0.248 (−0.001 to 0.003)
	2	0.357	−0.280 (−0.002 to 0.001)	0.994	−0.002 (−0.001 to 0.001)	0.377	0.210 (−0.001 to 0.003)
	3	0.354	−0.290 (−0.002 to 0.001)	0.755	0.064 (−0.001 to 0.002)	0.360	0.226 (−0.001 to 0.003)
	4	0.685	−0.129 (−0.001 to 0.001)	0.621	−0.126 (−0.002 to 0.001)	0.387	0.217 (−0.001 to 0.003)
	5	0.715	−0.165 (−0.002 to 0.001)	0.436	−0.276 (−0.003 to 0.002)	0.892	−0.043 (−0.003 to 0.003)
Phosphorus	1	0.324	0.250 (0.000 to 0.001)	0.238	0.262 (0.000 to 0.002)	0.036	−0.703 (−0.004 to 0.000)
	2	0.340	0.246 (0.000 to 0.001)	0.126	0.392 (0.000 to 0.002)	0.033	−0.731 (−0.004 to 0.000)
	3	0.351	0.245 (0.000 to 0.001)	0.129	0.322 (0.000 to 0.002)	0.037	−0.726 (−0.004 to 0.000)
	4	0.224	0.383 (0.000 to 0.001)	0.202	0.290 (0.000 to 0.002)	0.045	−0.710 (−0.004 to 0.000)
	5	0.453	0.291 (0.000 to 0.001)	0.323	0.305 (−0.001 to 0.002)	0.024	−1.020 (−0.005 to 0.000)
Potassium	1	0.556	0.137 (0.000 to 0.001)	0.229	0.327 (0.000 to 0.002)	0.997	0.001 (−0.001 to 0.001)
	2	0.555	0.140 (0.000 to 0.001)	0.116	0.415 (0.000 to 0.002)	0.995	−0.002 (−0.001 to 0.001)
	3	0.527	0.159 (0.000 to 0.001)	0.110	0.417 (0.000 to 0.002)	0.982	0.006 (−0.001 to 0.001)
	4	0.555	0.156 (0.000 to 0.001)	0.052	0.596 (0.000 to 0.003)	0.999	0.000 (−0.001 to 0.001)
	5	0.373	0.412 (−0.003 to 0.005)	0.089	0.644 (0.000 to 0.003)	0.733	−0.117 (−0.002 to 0.001)
Magnesium	1	0.314	0.244 (−0.001 to 0.004)	0.029	−0.618 (−0.018 to −0.001)	0.559	−0.100 (−0.007 to 0.004)
	2	0.312	0.251 (−0.001 to 0.004)	0.007	−0.790 (−0.021 to −0.004)	0.600	−0.090 (−0.007 to 0.004)
	3	0.345	0.241 (−0.001 to 0.004)	0.004	−0.835 (−0.022 to −0.004)	0.547	−0.126 (−0.008 to 0.005)
	4	0.207	0.327 (−0.001 to 0.004)	0.008	−0.815 (−0.022 to −0.004)	0.499	−0.148 (−0.009 to 0.004)
	5	0.502	0.232 (−0.003 to 0.005)	0.033	−0.901 (−0.027 to −0.001)	0.328	−0.246 (−0.012 to 0.004)
Iron	1	0.222	−0.349 (−0.086 to 0.021)	0.723	−0.089 (−0.092 to 0.065)	0.791	−0.066 (−0.085 to 0.065)
	2	0.277	−0.322 (−0.085 to 0.026)	0.425	−0.196 (−0.106 to 0.046)	0.753	−0.080 (−0.088 to 0.064)
	3	0.285	−0.324 (−0.087 to 0.027)	0.439	−0.188 (−0.104 to 0.047)	0.834	−0.056 (−0.089 to 0.072)
	4	0.078	−0.612 (−0.120 to 0.007)	0.394	−0.221 (−0.115 to 0.047)	0.815	−0.064 (−0.092 to 0.073)
	5	0.051	−1.066 (−0.196 to 0.001)	0.539	−0.189 (−0.127 to 0.069)	0.859	−0.058 (−0.108 to 0.091)
Iodine	1	0.836	0.049 (−0.011 to 0.013)	0.888	0.027 (−0.031 to 0.036)	0.809	0.054 (−0.024 to 0.030)
	2	0.808	0.059 (−0.011 to 0.014)	0.867	0.031 (−0.029 to 0.034)	0.679	0.092 (−0.023 to 0.034)
	3	0.804	0.061 (−0.011 to 0.014)	0.945	0.013 (−0.031 to 0.033)	0.687	0.096 (−0.023 to 0.034)
	4	0.956	0.014 (−0.013 to 0.013)	0.881	0.029 (−0.031 to 0.036)	0.660	0.108 (−0.023 to 0.036)
	5	0.861	−0.064 (−0.021 to 0.018)	0.793	0.064 (−0.038 to 0.049)	0.677	0.108 (−0.025 to 0.038)
Zinc	1	0.872	−0.043 (−0.100 to 0.086)	0.196	0.320 (−0.108 to 0.499)	0.476	0.288 (−0.177 to 0.456)
	2	0.857	−0.049 (−0.103 to 0.087)	0.118	0.372 (−0.062 to 0.517)	0.280	0.464 (−0.193 to 0.642)
	3	0.896	−0.037 (−0.105 to 0.092)	0.143	0.345 (−0.077 to 0.498)	0.363	0.418 (−0.246 to 0.651)
	4	0.776	−0.079 (−0.111 to 0.084)	0.142	0.373 (−0.083 to 0.538)	0.337	0.457 (−0.242 to 0.684)
	5	0.820	−0.110 (−0.196 to 0.158)	0.168	0.467 (−0.134 to 0.705)	0.115	1.042 (−0.135 to 1.143)
Selenium	1	0.160	0.324 (−0.002 to 0.011)	0.565	0.141 (−0.011 to 0.020)	0.375	0.175 (−0.009 to 0.020)
	2	0.163	0.327 (−0.002 to 0.012)	0.678	0.079 (−0.012 to 0.017)	0.489	0.171 (−0.010 to 0.020)
	3	0.176	0.325 (−0.002 to 0.012)	0.841	0.047 (−0.013 to 0.016)	0.529	0.160 (−0.010 to 0.020)
	4	0.349	0.226 (−0.004 to 0.010)	0.582	0.142 (−0.012 to 0.021)	0.629	0.128 (−0.012 to 0.020)
	5	0.532	−0.413 (−0.026 to 0.014)	0.834	0.074 (−0.020 to 0.025)	0.993	−0.003 (−0.022 to 0.022)

### 3.3 Association between mineral intake and HbA1c in the study group

In the NODCP group, there was a significant negative correlation between HbA1c levels and average phosphorus intake (*p* = 0.029 in Model 1, *p* = 0.043 in Model 2, and p = 0.043 in Model 3). However, there is no correlation between the intake of other study minerals in the NODCP group and HbA1c.

No correlation was found between the studied mineral intake and HbA1c levels in all models of the NCP and T2DM groups (Table 6).

**Table 6:** Associations between mineral intake and HbA1c in the research group.

Mineral	Model	NCP (*n* = 37)		NODCP (*n* = 35)		T2DM (*n* = 42)	
		*p*	*β* coefficient (95%CI)	*p*	*β* coefficient (95%CI)	*p*	*β* coefficient (95%CI)
Sodium	1	0.475	−0.145 (−0.759 to 0.364)	0.923	0.017 (−1.192 to 1.312)	0.860	0.052 (−0.417 to 0.496)
	2	0.698	0.064 (−0.367 to 0.542)	0.905	0.021 (−1.193 to 1.340)	0.890	−0.043 (−0.507 to 0.442)
	3	0.589	−0.107 (−0.696 to −0.405)	0.647	0.083 (−1.007 to 1.593)	0.944	−0.023 (−0.528 to 0.493)
	4	0.914	0.019 (−0.450 to 0.501)	0.639	0.099 (−1.183 to 1.185)	0.719	−0.131 (−0.668 to 0.468)
	5	0.849	0.031 (−0.405 to 0.490)	0.592	0.122 (−0.785 to 1.341)	0.732	−0.124 (−0.660 to 0.471)
Chlorine	1	0.781	−0.049 (−0.265 to 0.350)	0.818	−0.039 (−0.872 to 0.693)	0.769	0.076 (−0.196 to 0.263)
	2	0.577	−0.111 (−0.449 to 0.256)	0.860	−0.031 (−0.878 to 0.736)	0.766	0.079 (−0.202 to 0.272)
	3	0.912	0.018 (−0.269 to 0.300)	0.790	0.048 (−0.716 to 0.934)	0.893	0.038 (−0.239 to 0.273)
	4	0.524	−0.137 (−0.502 to 0.264)	0.951	0.012 (−0.901 to 0.957)	0.955	0.017 (−0.264 to 0.279)
	5	0.647	−0.093 (−0.445 to 0.282)	0.912	−0.022 (−0.998 to 0.896)	0.896	−0.040 (−0.291 to 0.256)
Calcium	1	0.111	−0.424 (−0.001 to 0.000)	0.810	−0.046 (−0.001 to 0.001)	0.360	0.221 (0.000 to 0.001)
	2	0.167	−0.362 (−0.001 to 0.000)	0.749	−0.062 (−0.001 to 0.001)	0.288	0.272 (0.000 to 0.001)
	3	0.218	−0.322 (−0.001 to 0.000)	0.529	−0.124 (−0.001 to 0.001)	0.356	0.252 (0.000 to 0.001)
	4	0.199	−0.374 (−0.001 to 0.000)	0.721	−0.089 (−0.002 to 0.001)	0.269	0.360 (0.000 to 0.001)
	5	0.225	−0.324 (−0.001 to 0.000)	0.738	0.085 (−0.002 to 0.001)	0.425	0.265 (0.000 to 0.001)
Phosphorus	1	0.484	−0.165 (−0.001 to 0.000)	**0.029**	−0.447 (−0.002 to 0.000)	0.642	0.158 (0.000 to 0.000)
	2	0.503	−0.154 (−0.001 to 0.000)	**0.043**	−0.423 (−0.002 to 0.000)	0.597	0.184 (0.000 to 0.000)
	3	0.500	−0.154 (−0.001 to 0.000)	**0.043**	−0.417 (−0.002 to 0.000)	0.609	0.194 (0.000 to 0.000)
	4	0.305	−0.296 (−0.001 to 0.000)	0.059	0.429 (−0.002 to 0.000)	0.604	0.206 (0.000 to 0.000)
	5	0.466	−0.173 (−0.001 to 0.000)	0.068	−0.462 (−0.002 to 0.000)	0.885	0.060 (0.000 to 0.000)
Potassium	1	0.716	0.076 (0.000 to 0.000)	0.946	0.016 (−0.001 to 0.001)	0.286	−0.287 (0.000 to 0.000)
	2	0.752	0.064 (0.000 to 0.000)	0.848	0.047 (−0.001 to 0.001)	0.335	−0.261 (0.000 to 0.000)
	3	0.997	0.001 (0.000 to 0.000)	0.852	0.045 (−0.001 to 0.001)	0.341	−0.273 (0.000 to 0.000)
	4	0.764	0.068 (0.000 to 0.000)	0.789	0.077 (−0.001 to 0.001)	0.635	−0.164 (0.000 to 0.000)
	5	0.944	−0.015 (0.000 to 0.000)	0.901	0.039 (−0.001 to 0.001)	0.662	−0.150 (0.000 to 0.000)
Magnesium	1	0.634	0.103 (−0.002 to 0.002)	0.566	0.139 (−0.005 to 0.008)	0.434	0.142 (−0.001 to 0.001)
	2	0.654	0.095 (−0.002 to 0.002)	0.760	0.079 (−0.006 to 0.008)	0.996	0.001 (−0.001 to 0.001)
	3	0.543	0.130 (−0.002 to 0.002)	0.637	0.121 (−0.005 to 0.008)	0.872	−0.038 (−0.001 to 0.001)
	4	0.815	0.054 (−0.002 to 0.002)	0.622	0.137 (−0.006 to 0.009)	0.520	−0.157 (−0.002 to 0.001)
	5	0.590	0.118 (−0.001 to 0.003)	0.560	0.186 (−0.006 to 0.011)	0.321	−0.258 (−0.001 to 0.001)
Iron	1	0.686	0.102 (−0.033 to 0.049)	0.569	−0.129 (−0.074 to 0.042)	0.075	−0.473 (−0.024 to 0.001)
	2	0.899	0.032 (−0.038 to 0.043)	0.478	−0.166 (−0.081 to 0.039)	0.145	−0.398 (−0.023 to 0.004)
	3	0.895	0.033 (−0.038 to 0.043)	0.452	−0.174 (−0.081 to 0.037)	0.163	−0.431 (−0.026 to 0.005)
	4	0.673	0.126 (−0.039 to 0.059)	0.574	−0.142 (−0.083 to 0.047)	0.182	−0.428 (−0.026 to 0.005)
	5	0.994	0.002 (−0.043 to 0.044)	0.610	−0.132 (−0.084 to 0.050)	0.140	−0.477 (−0.027 to 0.004)
Iodine	1	0.232	0.259 (−0.004 to 0.015)	0.672	−0.074 (−0.030 to 0.020)	0.615	−0.120 (−0.006 to 0.004)
	2	0.305	0.218 (−0.004 to 0.014)	0.680	−0.072 (−0.030 to 0.020)	0.680	−0.103 (−0.006 to 0.004)
	3	0.320	0.210 (−0.005 to 0.014)	0.751	−0.055 (−0.029 to 0.021)	0.796	−0.069 (−0.006 to 0.005)
	4	0.228	0.284 (−0.004 to 0.016)	0.632	−0.089 (−0.033 to 0.020)	0.972	−0.009 (−0.005 to 0.005)
	5	0.430	0.180 (−0.006 to 0.014)	0.616	−0.096 (−0.034 to 0.021)	0.952	0.016 (−0.005 to 0.006)
Zinc	1	0.954	0.015 (−0.074 to 0.079)	0.956	−0.012 (−0.229 to 0.217)	0.999	−0.001 (−0.056 to 0.056)
	2	0.873	0.040 (−0.069 to 0.081)	0.978	0.006 (−0.224 to 0.230)	0.767	−0.142 (−0.089 to 0.066)
	3	0.949	−0.016 (−0.079 to 0.074)	0.885	0.032 (−0.210 to 0.241)	0.953	−0.032 (−0.092 to 0.087)
	4	0.789	0.071 (−0.070 to 0.072)	0.929	−0.022 (−0.260 to 0.239)	0.520	−0.380 (−0.127 to 0.066)
	5	0.902	0.035 (−0.081 to 0.092)	0.815	−0.065 (−0.322 to 0.256)	0.783	−0.169 (−0.114 to 0.087)
Selenium	1	0.191	0.264 (−0.002 to 0.008)	0.343	−0.209 (−0.016 to 0.006)	0.395	0.221 (−0.001 to 0.004)
	2	0.168	0.273 (−0.002 to 0.008)	0.315	−0.225 (−0.017 to 0.006)	0.494	0.180 (−0.002 to 0.003)
	3	0.157	0.279 (−0.001 to 0.008)	0.427	−0.177 (−0.016 to 0.007)	0.659	0.137 (−0.002 to 0.004)
	4	0.183	0.288 (−0.002 to 0.009)	0.344	−0.241 (−0.019 to 0.007)	0.750	−0.109 (−0.004 to 0.003)
	5	0.572	0.178 (−0.006 to 0.010)	0.339	−0.251 (−0.020 to 0.007)	0.425	−0.305 (−0.005 to 0.002)

Bold represents statistical significance.

### 3.4 Associations between mineral intake and insulin traits in the study groups

There is a correlation between fasting insulin and average magnesium intake in the NODCP group (*p* = 0.038 in Model 1, *p* = 0.042 in Model 2, and p = 0.042 in Model 3). However, there is no correlation between the intake of other minerals in the NODCP group and fasting insulin (Table 7).

**Table 7:** Associations between mineral intake and fasting insulin in the study groups.

Mineral	Model	NCP (*n* = 37)		NODCP (*n* = 35)		T2DM (*n* = 42)	
		*p*	*β* coefficient (95%CI)	*p*	*β* coefficient (95%CI)	*p*	*β* coefficient (95%CI)
Sodium	1	0.824	−0.049 (−3.553 to 2.854)	0.375	0.151 (−2.002 to 5.145)	0.965	−0.011 (−3.759 to 3.601)
	2	0.880	0.026 (−2.352 to 2.731)	0.368	0.157 (−2.034 to 5.302)	0.940	−0.019 (−3.861 to 3.582)
	3	0.936	−0.018 (−3.453 to 3.194)	0.470	0.134 (−2.522 to 5.309)	0.828	−0.055 (−4.236 to 3,417)
	4	0.925	−0.023 (−2.350 to 2.144)	0.298	0.201 (−1.277 to 3.990)	0.865	−0.045 (−4.276 to 3.615)
	5	0.999	0.000 (−2.596 to 2.591)	0.533	0.131 (−3.105 to 5.839)	0.899	−0.030 (−3.833 to 3.381)
Chlorine	1	0.902	0.021 (−1.520 to 1.717)	0.135	0.250 (−0.555 to 3.936)	0.887	−0.030 (−1.987 to 1.726)
	2	0.930	−0.020 (−2.180 to 1.999)	0.198	0.217 (−0.807 to 3.746)	0.785	−0.060 (−2.172 to 1.655)
	3	0.766	0.053 (−1.413 to 1.902)	0.178	0.241 (−0.779 to 4.030)	0.775	−0.063 (−2.203 to 1.659)
	4	0.784	0.052 (−1.503 to 1.974)	0.147	0.276 (−0.693 to 4.418)	0.561	−0.135 (−2.597 to 1.439)
	5	0.723	−0.082 (−2.544 to 1.759)	0.194	0.260 (−0.945 to 4.454)	0.428	−0.169 (−2.571 to 1.124)
Calcium	1	0.948	0.019 (−0.005 to 0.005)	0.653	0.083 (−0.002 to 0.004)	0.260	−0.213 (−0.004 to 0.001)
	2	0.851	0.056 (−0.002 to 0.003)	0.699	0.074 (−0.003 to 0.004)	0.215	−0.243 (−0.005 to 0.001)
	3	0.937	0.024 (−0.005 to 0.005)	0.635	0.096 (−0.003 to 0.004)	0.252	−0.227 (−0.005 to 0.001)
	4	0.735	0.111 (−0.004 to 0.006)	0.544	0.122 (−0.002 to 0.004)	0.167	−0.288 (−0.005 to 0.001)
	5	0.859	0.056 (−0.005 to 0.006)	0.370	0.222 (−0.002 to 0.006)	0.099	−0.318 (−0.005 to 0.000)
Phosphorus	1	0.560	0.143 (−0.001 to 0.003)	0.544	−0.114 (−0.003 to 0.002)	0.090	−0.464 (−0.004 to 0.000)
	2	0.573	0.140 (−0.002 to 0.003)	0.561	−0.111 (−0.003 to 0.002)	0.075	−0.503 (−0.005 to 0.000)
	3	0.621	0.125 (−0.002 to 0.003)	0.581	−0.108 (−0.003 to 0.002)	0.104	−0.468 (−0.005 to 0.000)
	4	0.857	0.049 (−0.002 to 0.003)	0.404	−0.168 (−0.004 to 0.002)	0.181	−0.364 (−0.004 to 0.001)
	5	0.476	−0.216 (−0.003 to 0.002)	0.548	−0.126 (−0.003 to 0.002)	0.064	−0.515 (−0.005 to 0.000)
Potassium	1	0.471	0.163 (−0.001 to 0.003)	0.580	0.120 (−0.002 to 0.003)	0.751	0.073 (−0.001 to 0.002)
	2	0.515	0.149 (−0.001 to 0.003)	0.591	0.118 (−0.002 to 0.003)	0.761	0.070 (−0.001 to 0.002)
	3	0.445	0.181 (−0.001 to 0.003)	0.566	0.130 (−0.002 to 0.003)	0.723	0.083 (−0.001 to 0.002)
	4	−0.378	0.230 (−0.001 to 0.003)	0.557	0.132 (−0.002 to 0.003)	0.704	0.090 (−0.001 to 0.002)
	5	0.439	0.193 (−0.001 to 0.003)	0.699	0.099 (−0.002 to 0.003)	0.830	0.047 (−0.001 to 0.002)
Magnesium	1	0.101	0.401 (−0.002 to 0.021)	**0.038**	−0.513 (−0.038 to −0.001)	0.112	−0.243 (−0.014 to 0.002)
	2	0.113	0.392 (−0.002 to 0.021)	**0.042**	−0.534 (−0.040 to −0.001)	0.137	−0.236 (−0.014 to 0.002)
	3	0.154	0.362 (−0.003 to 0.020)	**0.042**	−0.552 (−0.041 to −0.001)	0.102	−0.304 (−0.017 to 0.002)
	4	0.180	0.352 (−0.004 to 0.021)	0.073	−0.489 (−0.039 to 0.002)	0.063	−0.364 (−0.018 to 0.001)
	5	0.272	0.278 (−0.006 to 0.019)	0.063	−0.525 (−0.041 to 0.001)	**0.041**	−0.369 (−0.018 to 0.000)
Iron	1	0.328	−0.238 (−0.305 to 0.106)	0.795	−0.057 (−0.187 to 0.145)	0.323	0.219 (−0.054 to 0.158)
	2	0.286	−0.266 (−0.321 to 0.099)	0.770	−0.066 (−0.195 to 0.146)	0.324	0.221 (−0.054 to 0.159)
	3	0.342	−0.242 (−0.317 to 0.114)	0.764	−0.069 (−0.200 to 0.148)	0.271	0.254 (−0.050 to 0.017)
	4	0.326	−0.262 (−0.335 to 0.117)	0.994	0.002 (−0.178 to 0.180)	0.264	0.277 (−0.052 to 0.184)
	5	0.906	−0.033 (−0.257 to 0.229)	0.989	0.003 (−0.183 to 0.186)	0.471	0.165 (−0.071 to 0.149)
Iodine	1	0.865	−0.041 (−0.059 to 0.050)	0.762	−0.052 (−0.083 to 0.061)	0.162	−0.269 (−0.062 to 0.011)
	2	0.793	−0.064 (−0.062 to 0.048)	0.764	−0.052 (−0.084 to 0.062)	0.261	−0.228 (−0.061 to 0.017)
	3	0.826	−0.054 (−0.062 to 0.050)	0.748	−0.057 (−0.087 to 0.063)	0.251	−0.235 (−0.062 to 0.017)
	4	0.923	−0.025 (−0.061 to 0.056)	0.595	−0.095 (−0.095 to 0.056)	0.314	−0.209 (−0.060 to 0.020)
	5	0.704	0.097 (−0.047 to 0.069)	0.525	−0.117 (−0.102 to 0.054)	0.414	−0.155 (−0.052 to 0.022)
Zinc	1	0.418	−0.229 (−0.623 to 0.267)	0.734	−0.073 (−0.755 to 0.539)	0.158	−0.340 (−0.640 to 0.108)
	2	0.464	−0.210 (−0.614 to 0.289)	0.752	−0.069 (−0.763 to 0.558)	0.649	−0.162 (−0.688 to 0.435)
	3	0.574	−0.167 (−0.600 to 0.340)	0.739	−0.074 (−0.783 to 0.563)	0.476	−0.280 (−0.838 to 0.401)
	4	0.524	−0.197 (−0.643 to 0.337)	0.505	−0.154 (−0.930 to 0.470)	0.503	−0.276 (−0.866 to 0.435)
	5	0.632	−0.141 (−0.580 to 0.361)	0.466	−0.173 (−0.979 to 0.463)	0.948	0.026 (−0.604 to 0.644)
Selenium	1	0.428	−0.177 (−0.041 to 0.018)	0.461	−0.159 (−0.044 to 0.021)	0.407	0.186 (−0.013 to 0.030)
	2	0.439	−0.174 (−0.041 to 0.019)	0.452	−0.167 (−0.046 to 0.021)	0.429	0.179 (−0.013 to 0.030)
	3	0.423	−0.183 (−0.043 to 0.018)	0.426	−0.182 (−0.048 to 0.021)	0.491	0.158 (−0.014 to 0.029)
	4	0.340	−0.231 (−0.048 to 0.017)	0.299	−0.243 (−0.053 to 0.017)	0.924	0.024 (−0.024 to 0.026)
	5	0.467	−0.169 (−0.043 to 0.020)	0.211	−0.317 (−0.062 to 0.014)	0.648	0.108 (−0.018 to 0.028)

Bold represents statistical significance.

There is a correlation between HOMA - *β* and average phosphorus intake in the T2DM group (Model 3 p = 0.042) (Table 8).

**Table 8:** Associations between mineral intake and HOMA-β in the study groups.

Mineral	*M*odel	NCP (*n* = 37)		NODCP (*n* = 35)		T2DM (*n* = 42)	
		*p*	*β* coefficient (95%CI)	*p*	*β* coefficient (95%CI)	*p*	*β* coefficient (95%CI)
Sodium	1	0.712	−0.078 (−14.22 to 9.865)	0.228	−0.221 (−15.02 to 3.731)	0.150	0.399 (−2.108 to 13.152)
	2	0.630	−0.091 (−13.26 to 8.158)	0.173	−0.271 (−17.08 to 3.242)	0.154	0.402 (−2.204 to 13.335)
	3	0.624	−0.094 (−13.48 to 8.226)	0.187	−0.263 (−16.93 to 3.493)	0.184	0.387 (−2.691 to 13.421)
	4	0.609	0.100 (−13.90 to 8.355)	0.182	−0.284 (−18.13 to 3.646)	0.159	0.420 (−2.413 to 14.042)
	5	0.978	−0.008 (−17.21 to 16.76)	0.257	−0.228 (−16.07 to 4.450)	0.223	0.357 (−3.184 to 13.051)
Chlorine	1	0.605	0.090 (−7.943 to 4.699)	0.211	−0.210 (−9.023 to 2.068)	0.094	−0.407 (−7.111 to 0.586)
	2	0.732	−0.080 (−9.982 to 7.126)	0.203	−0.233 (−9.870 to 2.180)	0.115	−0.396 (−7.166 to 0.822)
	3	0.735	−0.081 (−10.22 to 7.324)	0.277	−0.201 (−9.455 to 2.805)	0.120	−0.397 (−7.264 to 0.881)
	4	0.703	−0.094 (−10.76 to 7.403)	0.318	−0.189 (−9.378 to 3.145)	0.098	−0.438 (−0.006 to 0.006)
	5	0.684	−0.083 (−8.886 to 5.904)	0.169	−0.321 (−13.03 to 2.429)	0.070	−0.477 (−7.976 to 0.340)
Calcium	1	0.494	−0.191 (−0.023 to 0.012)	0.542	0.121 (−0.006 to 0.011)	0.987	−0.003 (−0.006 to 0.006)
	2	0.517	−0.198 (−0.026 to 0.013)	0.435	0.168 (−0.005 to 0.012)	0.969	0.008 (−0.006 to 0.006)
	3	0.506	−0.218 (−0.027 to 0.014)	0.315	0.226 (−0.005 to 0.014)	0.948	0.015 (−0.006 to 0.006)
	4	0.495	−0.230 (−0.028 to 0.014)	0.307	0.270 (−0.005 to 0.016)	0.978	0.006 (−0.006 to −0.006)
	5	0.656	−0.161 (−0.028 to 0.018)	0.294	0.284 (−0.005 to 0.017)	0.751	−0.072 (−0.007 to 0.005)
Phosphorus	1	0.765	−0.070 (−0.009 to 0.007)	0.806	−0.050 (−0.007 to 0.006)	0.098	0.502 (−0.001 to 0.009)
	2	0.777	−0.072 (−0.010 to 0.007)	0.843	−0.041 (−0.007 to 0.006)	0.101	0.517 (−0.001 to 0.009)
	3	0.842	−0.054 (−0.010 to 0.008)	0.753	−0.066 (−0.007 to 0.006)	0.103	0.531 (−0.001 to 0.010)
	4	0.727	−0.112 (−0.013 to 0.009)	0.792	−0.057 (−0.007 to 0.006)	0.088	0.570 (−0.001 to 0.010)
	5	0.848	−0.065 (−0.013 to 0.010)	0.898	−0.029 (−0.007 to 0.006)	**0.042**	0.687 (0.000 to 0.011)
Potassium	1	0.909	0.025 (−0.008 to 0.008)	0.717	0.084 (−0.005 to 0.007)	0.407	−0.212 (−0.005 to 0.002)
	2	0.908	0.029 (−0.009 to 0.010)	0.649	0.109 (−0.005 to 0.008)	0.416	−0.211 (−0.005 to 0.002)
	3	0.933	0.022 (−0.009 to 0.010)	0.925	0.024 (−0.006 to 0.007)	0.436	−0.206 (−0.005 to 0.002)
	4	0.960	0.013 (−0.010 to 0.550)	0.991	0.003 (−0.007 to 0.007)	0.421	−0.215 (−0.005 to 0.002)
	5	0.883	0.040 (−0.010 to 0.011)	0.946	0.019 (−0.007 to 0.007)	0.545	−0.158 (−0.005 to 0.002)
Magnesium	1	0.162	0.326 (−0.013 to 0.073)	0.571	−0.145 (−0.062 to 0.035)	0.715	−0.063 (−0.019 to 0.013)
	2	0.205	0.323 (−0.018 to 0.077)	0.493	−0.190 (−0.070 to 0.035)	0.709	−0.065 (−0.019 to 0.013)
	3	0.215	0.324 (−0.019 to 0.078)	0.572	−0.158 (−0.068 to 0.038)	0.653	−0.092 (−0.023 to 0.015)
	4	0.256	0.307 (−0.022 to 0.079)	0.566	−0.164 (−0.070 to 0.039)	0.555	−0.125 (−0.025 to 0.014)
	5	0.245	0.322 (−0.022 to 0.081)	0.520	−0.191 (−0.074 to 0.039)	0.416	−0.171 (−0.027 to 0.012)
Iron	1	0.920	−0.023 (−0.811 to 0.735)	0.955	−0.013 (−0.448 to 0.424)	0.598	−0.129 (−0.277 to 0.162)
	2	0.940	−0.019 (−0.886 to 0.822)	0.928	−0.022 (−0.472 to 0.432)	0.602	−0.130 (−0.281 to 0.165)
	3	0.963	0.012 (−0.903 to 0.863)	0.949	0.016 (−0.445 to 0.474)	0.651	−0.117 (−0.283 to 0.180)
	4	0.900	0.038 (−0.957 to 1.081)	0.924	0.024 (−0.450 to 0.494)	0.573	−0.149 (−0.303 to 0.171)
	5	0.964	0.014 (−1.028 to 1.074)	0.996	0.001 (−0.490 to 0.492)	0.911	0.031 (−0.234 to 0.262)
Iodine	1	0.124	−0.361 (−0.361 to 0.046)	0.371	0.165 (−0.105 to 0.272)	0.059	−0.410 (−0.149 to 0.003)
	2	0.156	−0.359 (−0.377 to 0.064)	0.416	0.154 (−0.116 to 0.272)	0.065	−0.425 (−0.157 to 0.005)
	3	0.161	−0.366 (−0.387 to 0.069)	0.428	0.151 (−0.119 to 0.271)	0.068	−0.428 (−0.159 to 0.006)
	4	0.219	−0.339 (−0.391 to 0.095)	0.474	0.140 (−0.131 to 0.272)	0.084	−0.411 (−0.157 to 0.010)
	5	0.231	−0.336 (−0.395 to 0.102)	0.451	0.152 (−0.131 to 0.285)	0.072	−0.422 (−0.158 to 0.007)
Zinc	1	0.489	0.188 (−1.102 to 2.244)	0.854	−0.042 (−1.850 to 1.543)	0.227	−0.322 (−1.245 to 0.307)
	2	0.518	0.193 (−1.270 to 2.446)	0.823	−0.053 (−1.940 to 1.556)	0.330	−0.390 (−1.739 to 0.604)
	3	0.518	0.199 (−1.308 to 2.518)	0.749	−0.076 (−2.041 to 1.488)	0.327	−0.437 (−1.940 to 0.668)
	4	0.505	0.211 (−1.329 to 2.613)	0.714	−0.090 (−2.156 to 1.500)	0.410	−0.376 (−1.886 to 0.792)
	5	0.418	0.283 (−1.315 to 3.038)	0.807	−0.063 (−2.151 to 1.692)	0.175	−0.654 (−2.358 to 0.452)
Selenium	1	0.750	−0.068 (−0.129 to 0.094)	0.081	−0.414 (−0.161 to 0.010)	0.611	0.126 (−0.033 to 0.055)
	2	0.762	−0.069 (−0.138 to 0.102)	0.071	−0.449 (−0.171 to 0.008)	0.610	0.128 (−0.034 to 0.056)
	3	0.810	−0.058 (−0.142 to 0.112)	0.061	−0.471 (−0.176 to 0.004)	0.641	0.120 (−0.036 to 0.057)
	4	0.859	−0.044 (−0.143 to 0.120)	0.067	−0.504 (−0.191 to 0.007)	0.790	0.071 (−0.042 to 0.054)
	5	0.990	−0.003 (−0.141 to 0.139)	0.080	−0.493 (−0.191 to 0.012)	0.637	−0.135 (−0.063 to 0.039)

Bold represents statistical significance.

## 4 Discussion

This study was the first to investigate the habitual mineral intake in patients with CP, comparing the average intake of 10 minerals across three groups: NODCP, T2DM, and NCP. Significant differences in magnesium and iron intake were found between the NODCP and NCP groups, while no such differences were observed between the T2DM and NCP groups. Additionally, a negative correlation between magnesium intake and fasting plasma glucose (FPG) was observed in the NODCP group, and a negative correlation between phosphorus intake and HbA1c was found in the NODCP group, whereas phosphorus intake in the T2DM group was negatively correlated with FPG. The relationship between iron and chronic pancreatitis and glucose metabolism.

Iron is a mineral that is an important component of certain proteins and a cofactor of enzymes involved in redox reactions. There are two forms of iron intake in the human body (heme and non-heme). Iron absorption occurs through the brush-like border membrane of the small intestine via the heme carrier protein (HCP 1) and divalent metal transporter protein (DMT 1). Non-heme iron requires the reduction of trivalent iron to divalent iron to be absorbed. The internal balance of iron is strictly regulated by ferritin, and the inflammatory state also leads to the upregulation of ferritin, triggered by pro-inflammatory cytokines such as interleukin-6 (25).

The disruption of iron homeostasis is related to various diseases. Animal experiments have confirmed that iron-overloaded mice exhibit increased pancreatic oxidative stress, elevated levels of malondialdehyde, decreased SOD and glutathione peroxidase activity, and observed acinar atrophy, extensive immune cell infiltration, and pancreatic fibrosis in the pancreas, indicating that secondary iron overload is a risk factor for pancreatitis (26). Research has shown that iron plays an important role in the calcification process related to pancreatic fibrosis, and compared to patients with CP, the iron content in pancreatic tissue of patients with calcified pancreatitis is significantly increased. Another study suggests that pancreatic exocrine dysfunction may be directly related to iron storage syndrome affecting the pancreas (27).

There is evidence that there is a relationship between increased iron intake and impaired glucose metabolism, leading to an increased risk of type 2 diabetes, gestational diabetes, and metabolic syndrome. An increase in the frequency of diabetes was also observed in iron overload diseases (hemochromatosis and *β* - thalassemia) due to insulin resistance and destruction of pancreatic *β* cells (28). Claire et al.’s research suggests a negative correlation between iron intake and fasting insulin levels in patients with hyperglycemia after acute pancreatitis, providing new insights into the role of iron intake in insulin sensitivity (29). However, research on the effect of iron on individual glucose metabolism after CP is very limited. Some argue that the primary risk factors for CP and pancreatic iron overload play a synergistic role in mediating the destruction of pancreatic beta cells. In addition, the persistent chronic inflammatory response of the pancreas and high levels of serum inflammatory factors (such as interleukin-6) can also lead to the upregulation of ferritin, further leading to disturbances in iron metabolism and glucose metabolism (19). In the future, more rigorous animal experiments and clinical studies are needed to prove the correlation between iron metabolism and hyperglycemia after CP, and investigate the role of low iron diet in preventing new diabetes after pancreatitis.

### 4.1 The relationship between magnesium and chronic pancreatitis and glucose metabolism

Magnesium is a divalent intracellular cation present in human cells. Magnesium plays a crucial role in many biological processes, including oxidative phosphorylation, energy generation, glycolysis, protein, and nucleic acid synthesis. Magnesium plays a role in the synthesis of adenosine triphosphate (ATP) in mitochondria to form Magnesium-ATP. In preclinical, epidemiological, and clinical human studies, magnesium deficiency, low serum magnesium levels, and reduced dietary magnesium intake are all associated with increased production of oxygen free radicals, low-grade systemic inflammation, and increased levels of inflammatory markers and pro-inflammatory molecules (30).

Consistent evidence shows that magnesium deficiency is related to the change in insulin sensitivity and the progress of diabetes, and magnesium metabolic disorder may mediate the impairment of insulin glucose uptake. Low levels of cellular and/or ionized plasma magnesium are found in patients with diabetes. The study confirmed that magnesium intake was negatively correlated with the incidence of new diabetes (31).

Henriette’s team’s study investigated the deficiency of trace elements in patients with CP, with magnesium deficiency accounting for approximately 17%. The demographic and disease characteristics of patients with CP and their relationship with trace element status were carried out to confirm that magnesium deficiency is related to the occurrence of diabetes in patients with CP (32).

As a continuously progressing chronic inflammatory fibrosis response, CP is characterized by oxidative stress throughout. Magnesium has a mild antioxidant effect, and magnesium deficiency not only increases oxidative stress but also reduces antioxidant defense ability. Providing appropriate doses of magnesium supplements for patients with suspected trace element deficiencies in CP has potential value in improving endocrine dysfunction, delaying pathological progression, and preventing complications. However, due to the exocrine dysfunction in patients with CP, further clinical trials are needed to explore the dosage of trace element supplementation.

### 4.2 The relationship between phosphorus and chronic pancreatitis and glucose metabolism

Phosphorus plays a crucial biochemical role by participating in cellular and extracellular metabolism as a component of nucleic acids, cell membranes, and high-energy compounds used in metabolism [such as adenosine triphosphate (ATP)], and by regulating the activity of many enzymes. CP may experience a certain degree of phosphorus deficiency due to inadequate exocrine function (33, 34). Claire et al. (9) studied that in patients with diabetes after acute pancreatitis, the phosphorus intake was significantly reduced compared with the normal blood glucose group after acute pancreatitis.

Wu et al. found that the serum phosphorus level in patients with type 2 diabetes was significantly reduced, suggesting that there may be a disorder of phosphorus metabolism. A nationwide cohort study found that the association between dietary phosphorus intake and new-onset diabetes was U-shaped. Among subjects with phosphorus intake<921.6 mg/day, the risk of new-onset diabetes significantly decreased with the increase of dietary phosphorus intake, while among subjects with phosphorus intake ≥921.6 mg/day, the risk increased with the increase of dietary phosphorus intake (35). It is suggested that phosphorus deficiency can reduce the risk of new-onset diabetes, which is consistent with our research results.

### 4.3 The relationship between other minerals and glucose metabolism, as well as the mutual influence between pancreatic endocrine/exocrine diseases and mineral metabolism

In this study, no correlation was found between the other seven minerals and glucose metabolism indicators in patients with chronic pancreatitis. Previous studies have suggested that elements such as zinc, selenium, and calcium play important roles in glucose metabolism. Zinc is involved in the synthesis of insulin and is co-released with insulin during secretion (36). It stabilizes insulin by promoting its dimerization and hexamerization, preventing its premature degradation. This stabilizing effect is critical for maintaining insulin availability and bioactivity. Insulin is stored in the pancreas in a complex with zinc ions, and upon secretion, this complex facilitates proper glucose homeostasis. Additionally, zinc participates in the regulation of glucose transporters and insulin receptors, contributing to glucose uptake and utilization in peripheral tissues like muscle and adipose tissue. Zinc has significant antioxidant properties, which help to reduce oxidative stress—a key factor in the development of diabetic complications, including diabetic retinopathy, neuropathy, nephropathy, and cardiovascular disease (37). Diseases such as chronic pancreatitis can affect the pancreas’s ability to store and secrete both zinc and insulin. Zinc absorption may also be impaired due to pancreatic insufficiency. Diabetic patients, especially those with poorly controlled blood sugar, are at a higher risk of zinc deficiency (38). Chronic hyperglycemia can lead to increased urinary excretion of zinc and may also impair zinc absorption in the gastrointestinal tract (39). This leads to a negative zinc balance in the body. Pancreatic endocrine and exocrine diseases aggravate the lack of zinc, thus promoting the development of diabetes nephropathy, diabetes retinopathy and other diseases.

Apart from zinc, diabetes mellitus affects the absorption and excretion of other minerals through various mechanisms, leading to mineral deficiencies that may exacerbate diabetes complications. First, diabetes often results in impaired intestinal absorption, gastrointestinal dysfunction, and drug interference, which reduce the absorption of minerals such as magnesium andcalcium. Meanwhile, hyperglycemia and kidney damage caused by diabetes can increase the excretion of these minerals, especially in patients with diabetic nephropathy. Excessive excretion of minerals, including magnesium, potassium, and calcium, may further aggravate metabolic disturbances, insulin resistance, and the development of diabetes-related complications. For example, magnesium deficiency not only leads to cardiovascular issues such as arrhythmias but may also worsen insulin resistance, exacerbating diabetes symptoms. Calcium deficiency is associated with osteoporosis and hypertension, and diabetes patients are more prone to these issues, increasing the risk of fractures. Additionally, chromium deficiency is closely linked to insulin resistance, making blood sugar control more difficult and potentially worsening the progression of diabetes. Therefore, diabetes patients often experience multiple mineral deficiencies, which not only affect daily metabolic functions but may also increase the risk of cardiovascular, skeletal, and immune system health issues. Timely monitoring and supplementation of minerals, particularly magnesium, calcium, and chromium, is crucial for diabetes management and the prevention of complications.

However, our research results are negative, which may be due to local dietary habits leading to relatively consistent intake of these minerals in the population. In the future, we need participants with more diverse dietary habits from a wider range of regions to join this study to verify the effects of more minerals on glucose metabolism in patients with chronic pancreatitis.

## 5 Conclusion

This study found significant differences in the intake of iron and phosphorus between the NODCP and NCP groups, with the NODCP group exhibiting a higher intake of these minerals. Additionally, in the NODCP group, a significant negative correlation was observed between magnesium intake and FPG levels, while phosphorus intake was negatively correlated with HbA1c levels. These findings highlight the potential role of specific mineral intake, particularly magnesium and phosphorus, in the regulation of glucose metabolism in patients with NODCP, These results highlight the potential impact of mineral intake on the development and progression of NODCP. Given these associations, future randomized controlled trials are warranted to explore the causal mechanisms between mineral intake and NODCP, which could support the development of targeted nutritional interventions for individuals at risk of developing NODCP.

## Data Availability

The raw data supporting the conclusions of this article will be made available by the authors, without undue reservation.
