# Supplementary material for: Association between dietary mineral intake and new onset diabetes/pre-diabetes after chronic pancreatitis
Source: Front Nutr. 2025 Jan 7;11:1461468. doi: 10.3389/fnut.2024.1461468 (PMC11747559; doi:10.3389/fnut.2024.1461468)
Supplement: Supplementary file 1 [file Supplementary_file_1.docx]

**Dietary habit survey**

- **Health Status**

**Sex：** □ Male □Female

Pregnant？：□ no □ yes ＿＿weeks of pregnancy

Menopause？：□ no □ yes Menopause for ＿＿ year(s)

**Physical examination：**Stature: ＿＿ m Abdominal circumference: ＿＿ cm

Blood pressure: ＿＿/＿＿ mmhg

Current weight: ＿＿ kg

Changes in weight after adulthood (18 years and above):

The minimum weight: ＿＿ kg; Age:＿＿

The maximum weight: ＿＿ kg; Age:＿＿

**Sleep quality:** Sleep for ＿＿ hours per night;

Siesta: □ no □ yes ＿＿ hours;


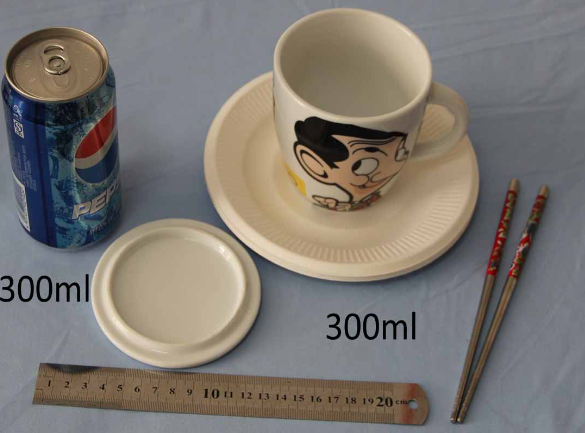
 **Drinking water:** Drink ＿＿ glasses of water per day(300ml per cup)

**Smoking:** □ never □ cessation status □ less than 1 cigarette per day

□ less than 10 cigarettes per day □10~20 cigarettes per day

□ More than 20 cigarettes per day

**Drinking:** □ never □ cessation status

□ be on the drink: baijiu (Chinese liquor) : ＿＿ ml per day

beer : ＿＿ ml per day

wine: ＿＿ ml per day

**Drink tea:** □ never □ cessation status □ frequently

black tea: ＿＿ glasses of water per day(300ml per cup)

green tea: ＿＿ glasses of water per day(300ml per cup)

scented tea: ＿＿ glasses of water per day(300ml per cup)

**Labor intensity:** 1. Work intensity:

□Light physical activity (retired, office workers, watch repairers, salespersons, teachers, etc.)

□Moderate physical activity (students, drivers, electricians, metalworkers, etc.)

□Heavy physical activity (farmers, athletes, dancers, steelworkers, loaders, etc.)

2.Daily working time of ＿＿ hour (at least 1 hour)

**Sports:** 1. Frequency of regular exercise: ＿＿ times a week hours one time

**Disease : 1.Cold** Number of colds in the past year:

**2.Diabetes** Do you have diabetes? □ No □ Yes □ Not sure

Years with diabetes:

Currently taking medication:

Most recent measurement of glycated hemoglobin (HbA1c): %.

Do you inject insulin? □ No □ Yes; Insulin has been injected for years.

Is there a family history of diabetes (blood relatives)?

□ No □ Yes □ Not sure

**3.Hypertension** Do you have hypertension? □ No □ Yes □ Not sure

Years with hypertension:

Currently taking medication.

Is there a family history of hypertension (blood relatives)? □ No □ Yes □ Not sure

**4.Hyperlipidemia** Do you have hyperlipidemia? □ No □ Yes □ Not sure

Currently taking medication: (If untreated, write "none")

**5.Fatty Liver** Do you have fatty liver? □ No □ Yes □ Not sure

Currently taking medication: (If untreated, write "none")

**6.Hepatitis** Do you have hepatitis? □ No □ Yes □ Not sure

Hepatitis A Hepatitis B Hepatitis C

**7.Heart Disease** Do you have heart disease? □ No □ Yes □ Not sure

**8.Other Diseases** Please list any other diseases you are suffering from:

**Eating Meals：** The number of people who regularly eat at home is＿＿people.

**Cooking Oil Situation Preferences:**

Like light flavors; Moderate oil usage; Like oily food.

What type of oil do you use at home, and how much? (If none, write 0)

Soybean oil, approximate monthly usage: ＿＿barrels

Peanut oil, approximate monthly usage:＿＿barrels

Corn oil, approximate monthly usage:＿＿barrels

Sunflower oil, approximate monthly usage:＿＿barrels

Salad oil, approximate monthly usage:＿＿barrels

Olive oil, approximate monthly usage:＿＿barrels

Other vegetable oils, approximate monthly usage:＿＿barrels

Animal fat, approximate monthly usage:＿＿kg

**Salt Usage Situation Preference:**

Light; Moderate; Heavy.

Monthly usage of salt is ＿＿g, soy sauce usage is＿＿g,

pickled vegetables and sauces＿＿g.

- **The situation of consuming grains and their products.**

**1.The situation of consuming rice (white rice)**


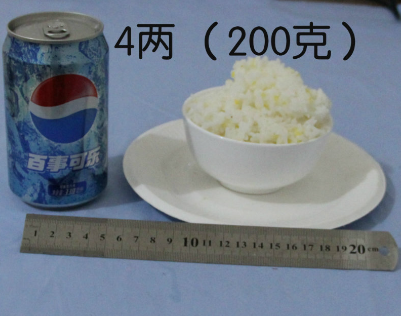


The rice in the picture weighs approximately 200 g

(1) The frequency of consumption is:

□ Never □ 1 to 3 times per month □ Once a week □ 2 to 3 times a week

□ 4 to 5 times a week □ Once a day □ Twice a day □ Three times a day or more

1. The amount of rice you consume each time is:

□ Less than or equal to 50 grams □ 50 to 150 grams □ 150 to 250 grams

□ 250 to 350 grams □ 350 to 450 grams □ 500 grams or more

**2.The situation of consuming** **wheat flour (including homemade products such as steamed buns, flower rolls, and pancakes)**


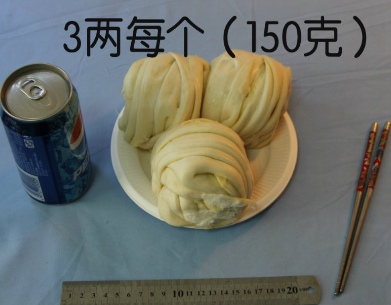


The wheat flour in the picture weighs approximately 150 g

(1) The frequency of consumption is:

□ Never □ 1 to 3 times per month □ Once a week □ 2 to 3 times a week

□ 4 to 5 times a week □ Once a day □ Twice a day □ Three times a day or more

1. The amount of wheat flour you consume each time is:

□ Less than or equal to 50 grams □ 50 to 150 grams □ 150 to 250 grams

□ 250 to 350 grams □ 350 to 450 grams □ 500 grams or more

**3.The situation of consuming millet.**


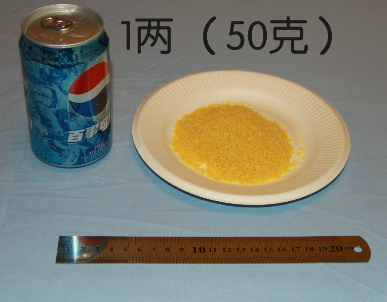


The millet in the picture weighs approximately 50 g

(1) The frequency of consumption is:

□ Never □ 1 to 3 times per month □ Once a week □ 2 to 3 times a week

□ 4 to 5 times a week □ Once a day □ Twice a day □ Three times a day or more

1. The amount of millet you consume each time is:

□ Less than or equal to 50 grams □ 50 to 150 grams □ 150 to 250 grams

□ 250 to 350 grams □ 350 to 450 grams □ 500 grams or more

**4.The situation of consuming corn.**


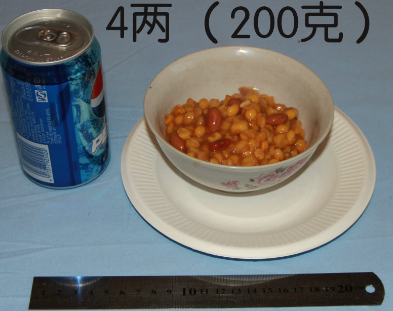
The corn in the picture weighs approximately 200 g

(1) The frequency of consumption is:

□ Never □ 1 to 3 times per month □ Once a week □ 2 to 3 times a week

□ 4 to 5 times a week □ Once a day □ Twice a day □ Three times a day or more

1. The amount of corn you consume each time is:

□ Less than or equal to 50 grams □ 50 to 150 grams □ 150 to 250 grams

□ 250 to 350 grams □ 350 to 450 grams □ 500 grams or more

**5.The situation of consuming** **noodles.**


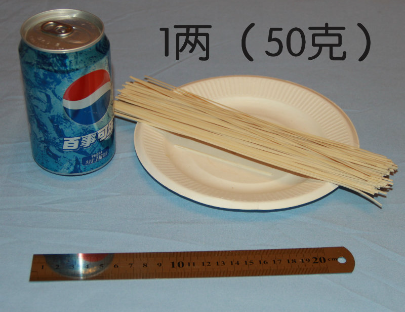
The noodles in the picture weighs approximately 50 g

The corn in the picture weighs approximately 200 g

(1) The frequency of consumption is:

□ Never □ 1 to 3 times per month □ Once a week □ 2 to 3 times a week

□ 4 to 5 times a week □ Once a day □ Twice a day □ Three times a day or more

(5)The amount of noodles you consume each time is:

□ Less than or equal to 50 grams □ 50 to 150 grams □ 150 to 250 grams

□ 250 to 350 grams □ 350 to 450 grams □ 500 grams or more

- **The situation of consuming starches from tubers and their products.**

1. **The situation of consuming tubers (potatoes, taro).**


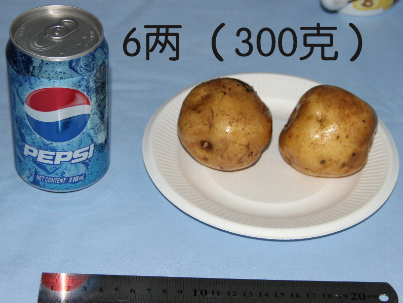
300g

(1) The frequency of consumption is:

□ Never □ 1 to 3 times per month □ Once a week □ 2 to 3 times a week

□ 4 to 5 times a week □ Once a day □ Twice a day □ Three times a day or more

(5)The amount of tubers you consume each time is:

□ Less than or equal to 50 grams □ 50 to 150 grams □ 150 to 250 grams

□ 250 to 350 grams □ 350 to 450 grams □ 500 grams or more

1. **The situation of consuming vermicelli.**


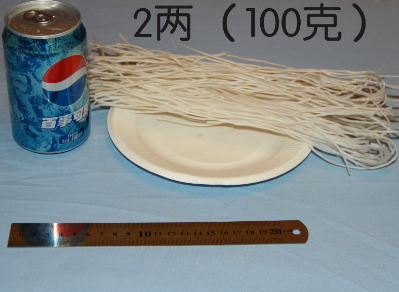
100g

(1) The frequency of consumption is:

□ Never □ 1 to 3 times per month □ Once a week □ 2 to 3 times a week

□ 4 to 5 times a week □ Once a day □ Twice a day □ Three times a day or more

(5)The amount of vermicelli you consume each time is:

□ Less than or equal to 50 grams □ 50 to 150 grams □ 150 to 250 grams

□ 250 to 350 grams □ 350 to 450 grams □ 500 grams or more

- **The situation of consuming vermicelli.**

1. **The situation of consuming tofu.**


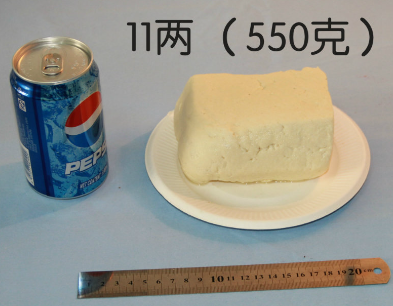
550g

(1) The frequency of consumption is:

□ Never □ 1 to 3 times per month □ Once a week □ 2 to 3 times a week

□ 4 to 5 times a week □ Once a day □ Twice a day □ Three times a day or more

(5)The amount of tofu you consume each time is:

□ Less than or equal to 50 grams □ 50 to 150 grams □ 150 to 250 grams

□ 250 to 350 grams □ 350 to 450 grams □ 500 grams or more

1. **The consumption of mung beans.**


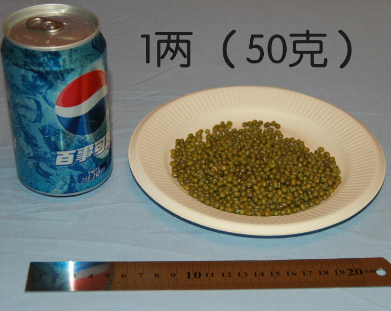
50g

(1) The frequency of consumption is:

□ Never □ 1 to 3 times per month □ Once a week □ 2 to 3 times a week

□ 4 to 5 times a week □ Once a day □ Twice a day □ Three times a day or more

(5)The amount of beans you consume each time is:

□ Less than or equal to 50 grams □ 50 to 150 grams □ 150 to 250 grams

□ 250 to 350 grams □ 350 to 450 grams □ 500 grams or more

1. **The consumption of soybean milk**


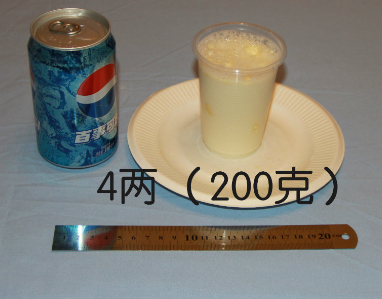
200g

(1) The frequency of consumption is:

□ Never □ 1 to 3 times per month □ Once a week □ 2 to 3 times a week

□ 4 to 5 times a week □ Once a day □ Twice a day □ Three times a day or more

(5)The amount of soybean milk you consume each time is:

□ Less than or equal to 50 grams □ 50 to 150 grams □ 150 to 250 grams

□ 250 to 350 grams □ 350 to 450 grams □ 500 grams or more

1. **The consumption of soybeans.**


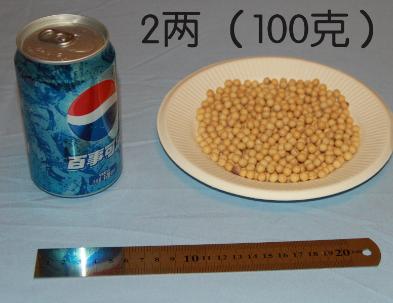
100g

(1) The frequency of consumption is:

□ Never □ 1 to 3 times per month □ Once a week □ 2 to 3 times a week

□ 4 to 5 times a week □ Once a day □ Twice a day □ Three times a day or more

(5)The amount of soybeans you consume each time is:

□ Less than or equal to 50 grams □ 50 to 150 grams □ 150 to 250 grams

□ 250 to 350 grams □ 350 to 450 grams □ 500 grams or more

1. **The consumption of other legumes (such as adzuki beans, kidney beans, black beans, and red bean paste).**


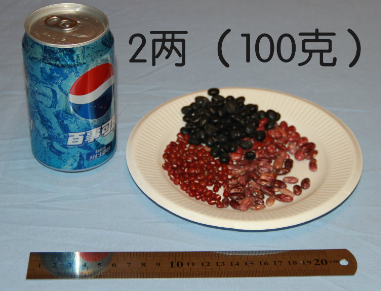
100g

(1) The frequency of consumption is:

□ Never □ 1 to 3 times per month □ Once a week □ 2 to 3 times a week

□ 4 to 5 times a week □ Once a day □ Twice a day □ Three times a day or more

(5)The amount of other legumes you consume each time is:

□ Less than or equal to 50 grams □ 50 to 150 grams □ 150 to 250 grams

□ 250 to 350 grams □ 350 to 450 grams □ 500 grams or more

- **The consumption of vegetable foods.**

1. **The consumption of carrots.**


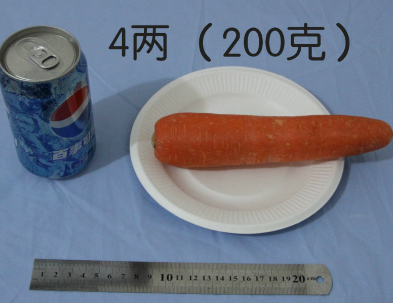
200g

(1) The frequency of consumption is:

□ Never □ 1 to 3 times per month □ Once a week □ 2 to 3 times a week

□ 4 to 5 times a week □ Once a day □ Twice a day □ Three times a day or more

(5)The amount of carrots you consume each time is:

□ Less than or equal to 50 grams □ 50 to 150 grams □ 150 to 250 grams

□ 250 to 350 grams □ 350 to 450 grams □ 500 grams or more

1. **The consumption of radishes.**


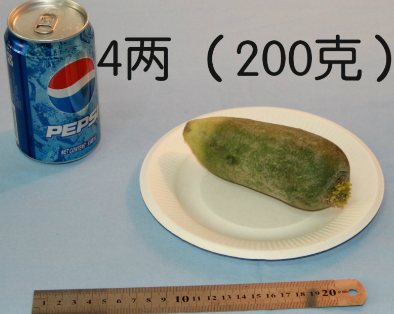
200g

(1) The frequency of consumption is:

□ Never □ 1 to 3 times per month □ Once a week □ 2 to 3 times a week

□ 4 to 5 times a week □ Once a day □ Twice a day □ Three times a day or more

(5)The amount of radishes you consume each time is:

□ Less than or equal to 50 grams □ 50 to 150 grams □ 150 to 250 grams

□ 250 to 350 grams □ 350 to 450 grams □ 500 grams or more

1. **The consumption of bean sprouts.**


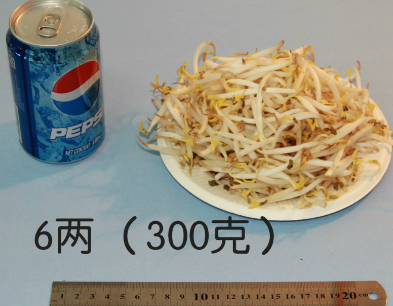
300g

(1) The frequency of consumption is:

□ Never □ 1 to 3 times per month □ Once a week □ 2 to 3 times a week

□ 4 to 5 times a week □ Once a day □ Twice a day □ Three times a day or more

(5)The amount of bean sprouts you consume each time is:

□ Less than or equal to 50 grams □ 50 to 150 grams □ 150 to 250 grams

□ 250 to 350 grams □ 350 to 450 grams □ 500 grams or more

1. **The consumption of string beans.**


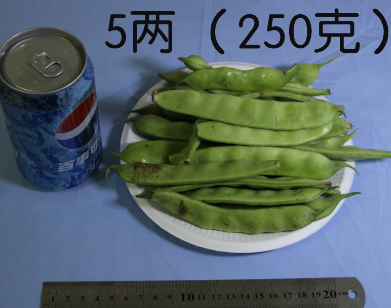
250g

(1) The frequency of consumption is:

□ Never □ 1 to 3 times per month □ Once a week □ 2 to 3 times a week

□ 4 to 5 times a week □ Once a day □ Twice a day □ Three times a day or more

(5)The amount of string beans you consume each time is:

□ Less than or equal to 50 grams □ 50 to 150 grams □ 150 to 250 grams

□ 250 to 350 grams □ 350 to 450 grams □ 500 grams or more

1. **The consumption of eggplants.**


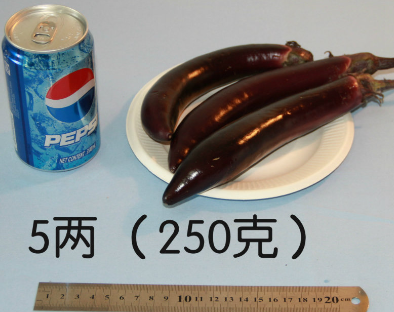
250g

(1) The frequency of consumption is:

□ Never □ 1 to 3 times per month □ Once a week □ 2 to 3 times a week

□ 4 to 5 times a week □ Once a day □ Twice a day □ Three times a day or more

(5)The amount of eggplants you consume each time is:

□ Less than or equal to 50 grams □ 50 to 150 grams □ 150 to 250 grams

□ 250 to 350 grams □ 350 to 450 grams □ 500 grams or more

1. **The consumption of cucumbers.**


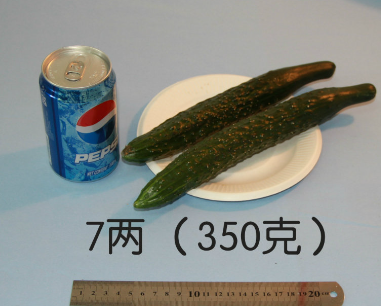
350g

(1) The frequency of consumption is:

□ Never □ 1 to 3 times per month □ Once a week □ 2 to 3 times a week

□ 4 to 5 times a week □ Once a day □ Twice a day □ Three times a day or more

(5)The amount of cucumbers you consume each time is:

□ Less than or equal to 50 grams □ 50 to 150 grams □ 150 to 250 grams

□ 250 to 350 grams □ 350 to 450 grams □ 500 grams or more

1. **The consumption of pumpkin (squash).**


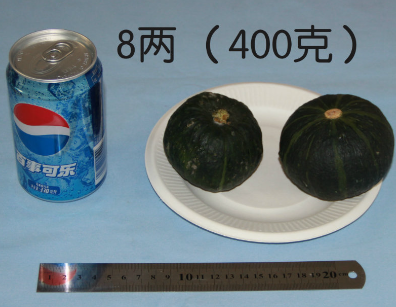
400g

(1) The frequency of consumption is:

□ Never □ 1 to 3 times per month □ Once a week □ 2 to 3 times a week

□ 4 to 5 times a week □ Once a day □ Twice a day □ Three times a day or more

(5)The amount of pumpkin you consume each time is:

□ Less than or equal to 50 grams □ 50 to 150 grams □ 150 to 250 grams

□ 250 to 350 grams □ 350 to 450 grams □ 500 grams or more

1. **The consumption of bell peppers (large green peppers).**


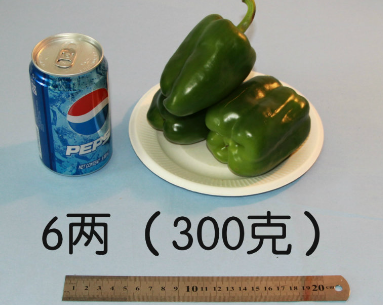
300g

(1) The frequency of consumption is:

□ Never □ 1 to 3 times per month □ Once a week □ 2 to 3 times a week

□ 4 to 5 times a week □ Once a day □ Twice a day □ Three times a day or more

(5)The amount of bell peppers you consume each time is:

□ Less than or equal to 50 grams □ 50 to 150 grams □ 150 to 250 grams

□ 250 to 350 grams □ 350 to 450 grams □ 500 grams or more

1. **The consumption of garlic.**


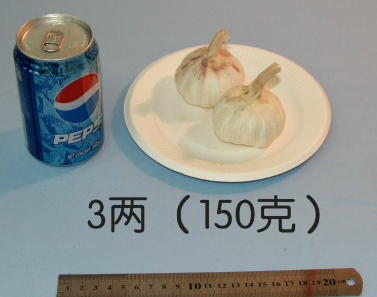
150g

(1) The frequency of consumption is:

□ Never □ 1 to 3 times per month □ Once a week □ 2 to 3 times a week

□ 4 to 5 times a week □ Once a day □ Twice a day □ Three times a day or more

(5)The amount of garlic you consume each time is:

□ Less than or equal to 50 grams □ 50 to 150 grams □ 150 to 250 grams

□ 250 to 350 grams □ 350 to 450 grams □ 500 grams or more

1. **The consumption of napa cabbage.**


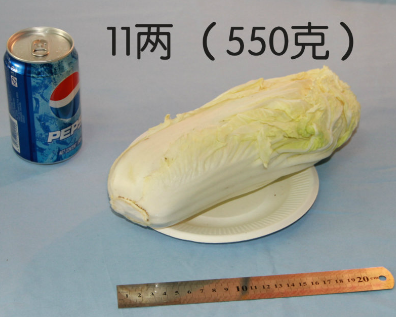
550g

(1) The frequency of consumption is:

□ Never □ 1 to 3 times per month □ Once a week □ 2 to 3 times a week

□ 4 to 5 times a week □ Once a day □ Twice a day □ Three times a day or more

(5)The amount of napa cabbage you consume each time is:

□ Less than or equal to 50 grams □ 50 to 150 grams □ 150 to 250 grams

□ 250 to 350 grams □ 350 to 450 grams □ 500 grams or more

1. **The consumption of spinach.**


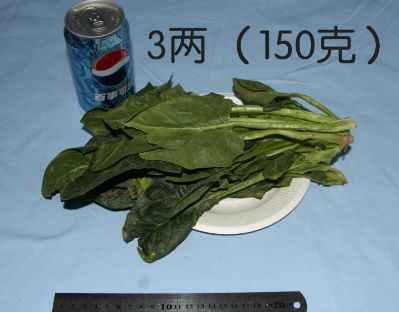
150g

(1) The frequency of consumption is:

□ Never □ 1 to 3 times per month □ Once a week □ 2 to 3 times a week

□ 4 to 5 times a week □ Once a day □ Twice a day □ Three times a day or more

(5)The amount of spinach you consume each time is:

□ Less than or equal to 50 grams □ 50 to 150 grams □ 150 to 250 grams

□ 250 to 350 grams □ 350 to 450 grams □ 500 grams or more

1. **The consumption of cabbage.**


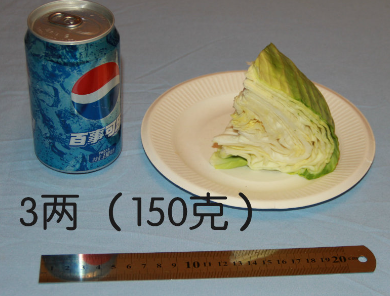
150g

(1) The frequency of consumption is:

□ Never □ 1 to 3 times per month □ Once a week □ 2 to 3 times a week

□ 4 to 5 times a week □ Once a day □ Twice a day □ Three times a day or more

(5)The amount of cabbage you consume each time is:

□ Less than or equal to 50 grams □ 50 to 150 grams □ 150 to 250 grams

□ 250 to 350 grams □ 350 to 450 grams □ 500 grams or more

1. **The consumption of celery.**


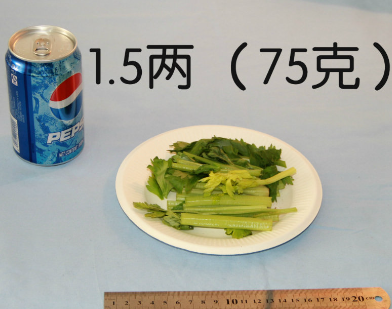
75g

(1) The frequency of consumption is:

□ Never □ 1 to 3 times per month □ Once a week □ 2 to 3 times a week

□ 4 to 5 times a week □ Once a day □ Twice a day □ Three times a day or more

(5)The amount of celery you consume each time is:

□ Less than or equal to 50 grams □ 50 to 150 grams □ 150 to 250 grams

□ 250 to 350 grams □ 350 to 450 grams □ 500 grams or more

1. **The consumption of rapeseed greens.**


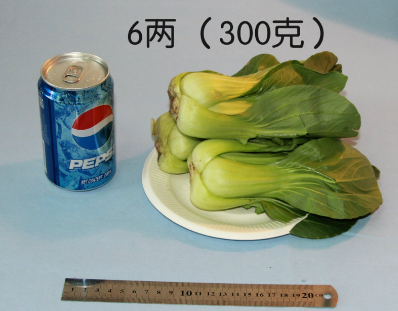
300g

(1) The frequency of consumption is:

□ Never □ 1 to 3 times per month □ Once a week □ 2 to 3 times a week

□ 4 to 5 times a week □ Once a day □ Twice a day □ Three times a day or more

(5)The amount of rapeseed greens you consume each time is:

□ Less than or equal to 50 grams □ 50 to 150 grams □ 150 to 250 grams

□ 250 to 350 grams □ 350 to 450 grams □ 500 grams or more

1. **The consumption of zucchini.**


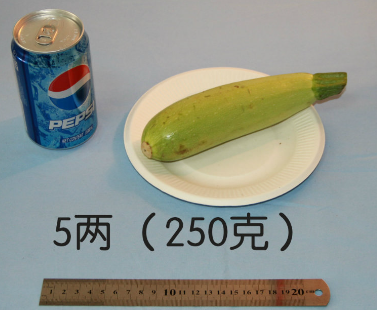
250g

(1) The frequency of consumption is:

□ Never □ 1 to 3 times per month □ Once a week □ 2 to 3 times a week

□ 4 to 5 times a week □ Once a day □ Twice a day □ Three times a day or more

(5)The amount of zucchini you consume each time is:

□ Less than or equal to 50 grams □ 50 to 150 grams □ 150 to 250 grams

□ 250 to 350 grams □ 350 to 450 grams □ 500 grams or more

1. **The consumption of tomatoes.**


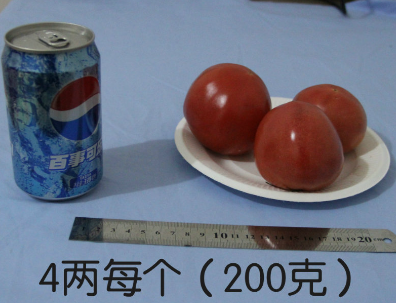
200g

(1) The frequency of consumption is:

□ Never □ 1 to 3 times per month □ Once a week □ 2 to 3 times a week

□ 4 to 5 times a week □ Once a day □ Twice a day □ Three times a day or more

(5)The amount of tomatoes you consume each time is:

□ Less than or equal to 50 grams □ 50 to 150 grams □ 150 to 250 grams

□ 250 to 350 grams □ 350 to 450 grams □ 500 grams or more

1. **The consumption of cauliflower.**


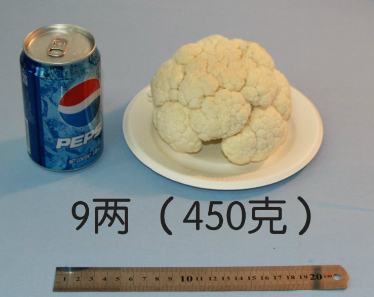
450g

(1) The frequency of consumption is:

□ Never □ 1 to 3 times per month □ Once a week □ 2 to 3 times a week

□ 4 to 5 times a week □ Once a day □ Twice a day □ Three times a day or more

(5)The amount of cauliflower you consume each time is:

□ Less than or equal to 50 grams □ 50 to 150 grams □ 150 to 250 grams

□ 250 to 350 grams □ 350 to 450 grams □ 500 grams or more

1.
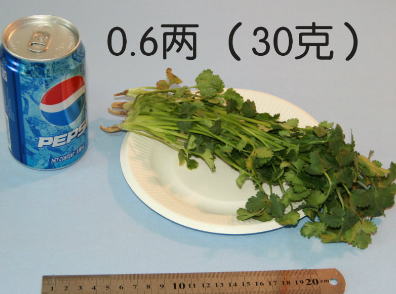
**The consumption of cilantro.**

(1) The frequency of consumption is:

□ Never □ 1 to 3 times per month □ Once a week □ 2 to 3 times a week

□ 4 to 5 times a week □ Once a day □ Twice a day □ Three times a day or more

(5)The amount of cilantro you consume each time is:

□ Less than or equal to 20 grams □ 20 to 30 grams □ 40 to 50 grams

□ 60 to 70 grams □ 80 to 90 grams □ 100 grams or more

1. **The consumption of lettuce.**


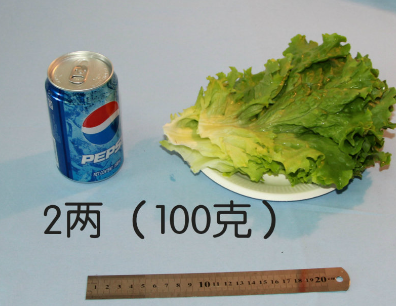
100g

(1) The frequency of consumption is:

□ Never □ 1 to 3 times per month □ Once a week □ 2 to 3 times a week

□ 4 to 5 times a week □ Once a day □ Twice a day □ Three times a day or more

(5)The amount of lettuce you consume each time is:

□ Less than or equal to 50 grams □ 50 to 150 grams □ 150 to 250 grams

□ 250 to 350 grams □ 350 to 450 grams □ 500 grams or more

1. **The consumption of onions.**


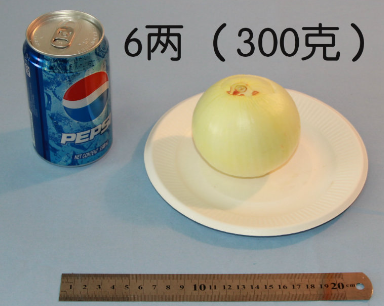
300g

(1) The frequency of consumption is:

□ Never □ 1 to 3 times per month □ Once a week □ 2 to 3 times a week

□ 4 to 5 times a week □ Once a day □ Twice a day □ Three times a day or more

(5)The amount of onions you consume each time is:

□ Less than or equal to 50 grams □ 50 to 150 grams □ 150 to 250 grams

□ 250 to 350 grams □ 350 to 450 grams □ 500 grams or more

1. **The consumption of scallions (green onions).**


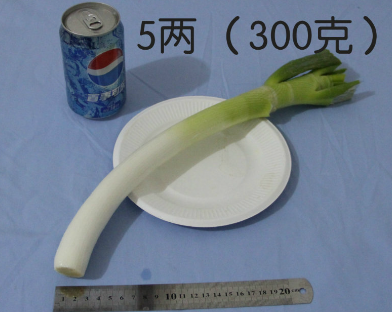
300g

(1) The frequency of consumption is:

□ Never □ 1 to 3 times per month □ Once a week □ 2 to 3 times a week

□ 4 to 5 times a week □ Once a day □ Twice a day □ Three times a day or more

(5)The amount of scallions you consume each time is:

□ Less than or equal to 20 grams □ 20 to 30 grams □ 40 to 50 grams

□ 60 to 70 grams □ 80 to 90 grams □ 100 grams or more

1. **The consumption of lotus root.**


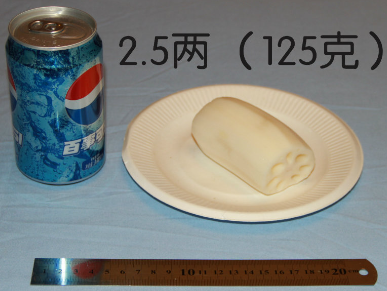
125g

(1) The frequency of consumption is:

□ Never □ 1 to 3 times per month □ Once a week □ 2 to 3 times a week

□ 4 to 5 times a week □ Once a day □ Twice a day □ Three times a day or more

(5)The amount of lotus root you consume each time is:

□ Less than or equal to 50 grams □ 50 to 150 grams □ 150 to 250 grams

□ 250 to 350 grams □ 350 to 450 grams □ 500 grams or more

- **The consumption of mushrooms and algae.**

1. **The consumption of fresh mushrooms**


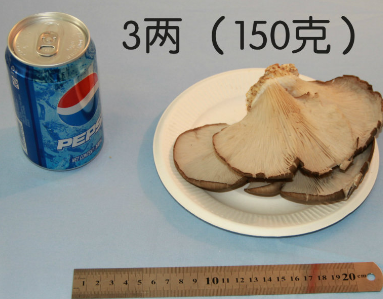
150g

(1) The frequency of consumption is:

□ Never □ 1 to 3 times per month □ Once a week □ 2 to 3 times a week

□ 4 to 5 times a week □ Once a day □ Twice a day □ Three times a day or more

(5)The amount of fresh mushrooms you consume each time is:

□ Less than or equal to 50 grams □ 50 to 150 grams □ 150 to 250 grams

□ 250 to 350 grams □ 350 to 450 grams □ 500 grams or more

1. **The consumption of kelp**


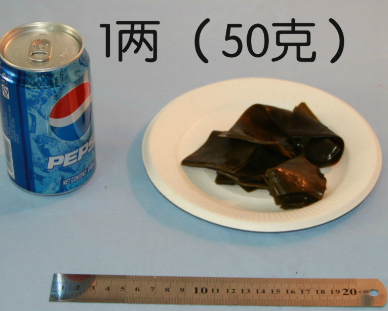
50g

(1) The frequency of consumption is:

□ Never □ 1 to 3 times per month □ Once a week □ 2 to 3 times a week

□ 4 to 5 times a week □ Once a day □ Twice a day □ Three times a day or more

(5)The amount of kelp you consume each time is:

□ Less than or equal to 50 grams □ 50 to 150 grams □ 150 to 250 grams

□ 250 to 350 grams □ 350 to 450 grams □ 500 grams or more

1.
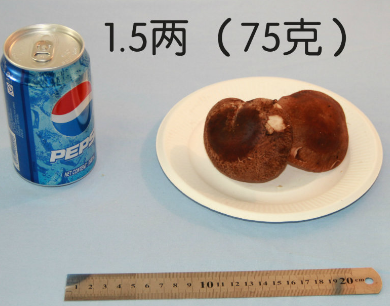
**The consumption of shiitake mushrooms**

75g

(1) The frequency of consumption is:

□ Never □ 1 to 3 times per month □ Once a week □ 2 to 3 times a week

□ 4 to 5 times a week □ Once a day □ Twice a day □ Three times a day or more

(5)The amount of shiitake mushrooms you consume each time is:

□ Less than or equal to 50 grams □ 50 to 150 grams □ 150 to 250 grams

□ 250 to 350 grams □ 350 to 450 grams □ 500 grams or more

- **The situation of consuming fresh fruits.**

1. **The situation of consuming apples**


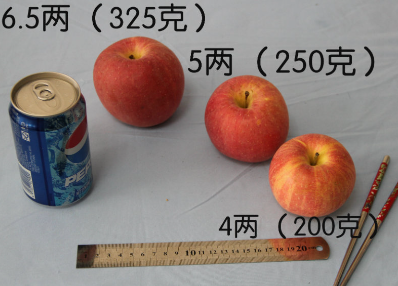
200g,250g,325g

(1) The frequency of consumption is:

□ Never □ 1 to 3 times per month □ Once a week □ 2 to 3 times a week

□ 4 to 5 times a week □ Once a day □ Twice a day □ Three times a day or more

(5)The amount of apples you consume each time is:

□ Less than or equal to 50 grams □ 50 to 150 grams □ 150 to 250 grams

□ 250 to 350 grams □ 350 to 450 grams □ 500 grams or more

1. **The situation of consuming pears**


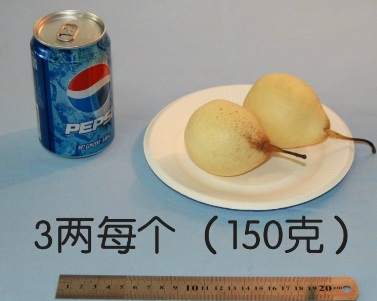
150g

(1) The frequency of consumption is:

□ Never □ 1 to 3 times per month □ Once a week □ 2 to 3 times a week

□ 4 to 5 times a week □ Once a day □ Twice a day □ Three times a day or more

(5)The amount of pears you consume each time is:

□ Less than or equal to 50 grams □ 50 to 150 grams □ 150 to 250 grams

□ 250 to 350 grams □ 350 to 450 grams □ 500 grams or more

1. **The situation of consuming bananas**


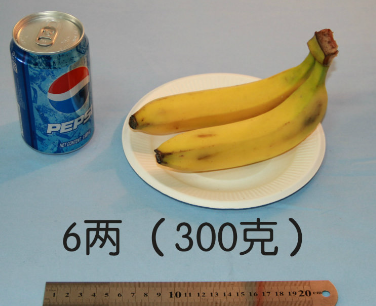
300g

(1) The frequency of consumption is:

□ Never □ 1 to 3 times per month □ Once a week □ 2 to 3 times a week

□ 4 to 5 times a week □ Once a day □ Twice a day □ Three times a day or more

(5)The amount of bananas you consume each time is:

□ Less than or equal to 50 grams □ 50 to 150 grams □ 150 to 250 grams

□ 250 to 350 grams □ 350 to 450 grams □ 500 grams or more

1. **The situation of consuming dates**


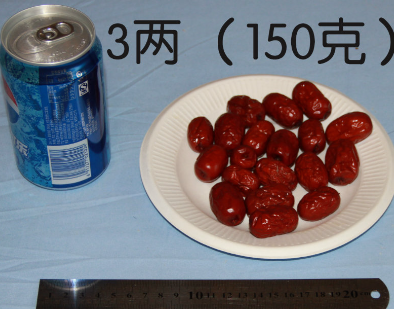
150g

(1) The frequency of consumption is:

□ Never □ 1 to 3 times per month □ Once a week □ 2 to 3 times a week

□ 4 to 5 times a week □ Once a day □ Twice a day □ Three times a day or more

(5)The amount of dates you consume each time is:

□ Less than or equal to 50 grams □ 50 to 150 grams □ 150 to 250 grams

□ 250 to 350 grams □ 350 to 450 grams □ 500 grams or more

1. **The situation of consuming citrus fruits**


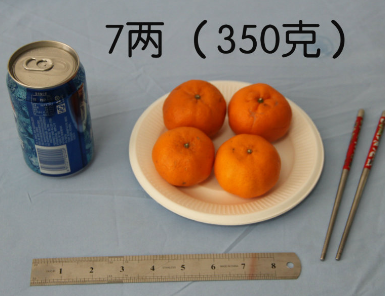
350g

(1) The frequency of consumption is:

□ Never □ 1 to 3 times per month □ Once a week □ 2 to 3 times a week

□ 4 to 5 times a week □ Once a day □ Twice a day □ Three times a day or more

(5)The amount of citrus fruits you consume each time is:

□ Less than or equal to 50 grams □ 50 to 150 grams □ 150 to 250 grams

□ 250 to 350 grams □ 350 to 450 grams □ 500 grams or more

1. **The situation of consuming grapes**


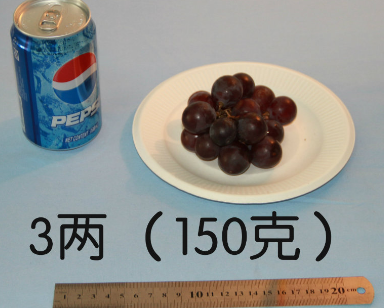
150g

(1) The frequency of consumption is:

□ Never □ 1 to 3 times per month □ Once a week □ 2 to 3 times a week

□ 4 to 5 times a week □ Once a day □ Twice a day □ Three times a day or more

(5)The amount of grapes you consume each time is:

□ Less than or equal to 50 grams □ 50 to 150 grams □ 150 to 250 grams

□ 250 to 350 grams □ 350 to 450 grams □ 500 grams or more

1. **The situation of consuming strawberries**


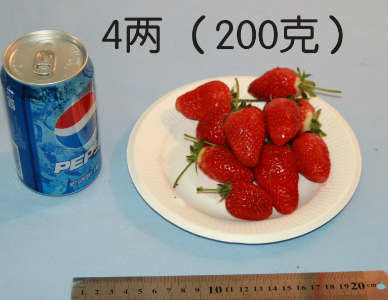
200g

(1) The frequency of consumption is:

□ Never □ 1 to 3 times per month □ Once a week □ 2 to 3 times a week

□ 4 to 5 times a week □ Once a day □ Twice a day □ Three times a day or more

(5)The amount of strawberries you consume each time is:

□ Less than or equal to 50 grams □ 50 to 150 grams □ 150 to 250 grams

□ 250 to 350 grams □ 350 to 450 grams □ 500 grams or more

1. **The situation of consuming watermelon**


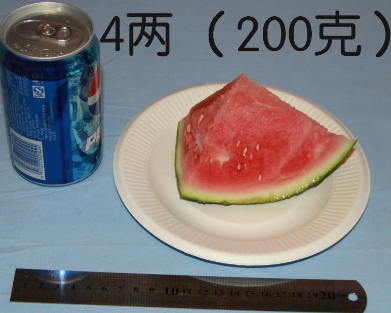
200g

(1) The frequency of consumption is:

□ Never □ 1 to 3 times per month □ Once a week □ 2 to 3 times a week

□ 4 to 5 times a week □ Once a day □ Twice a day □ Three times a day or more

(5)The amount of watermelon you consume each time is:

□ Less than or equal to 50 grams □ 50 to 150 grams □ 150 to 250 grams

□ 250 to 350 grams □ 350 to 450 grams □ 500 grams or more

1. **The situation of consuming longan**


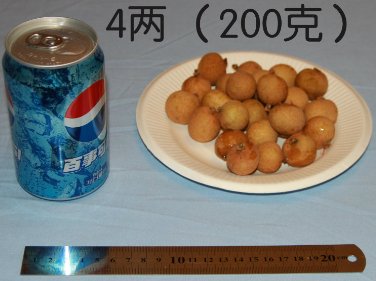
200g

(1) The frequency of consumption is:

□ Never □ 1 to 3 times per month □ Once a week □ 2 to 3 times a week

□ 4 to 5 times a week □ Once a day □ Twice a day □ Three times a day or more

(5)The amount of longan you consume each time is:

□ Less than or equal to 50 grams □ 50 to 150 grams □ 150 to 250 grams

□ 250 to 350 grams □ 350 to 450 grams □ 500 grams or more

1. **The situation of consuming peaches**


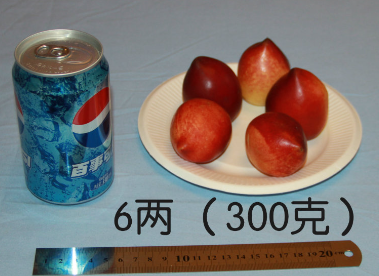
300g

(1) The frequency of consumption is:

□ Never □ 1 to 3 times per month □ Once a week □ 2 to 3 times a week

□ 4 to 5 times a week □ Once a day □ Twice a day □ Three times a day or more

(5)The amount of peaches you consume each time is:

□ Less than or equal to 50 grams □ 50 to 150 grams □ 150 to 250 grams

□ 250 to 350 grams □ 350 to 450 grams □ 500 grams or more

1. **The situation of consuming pineapples**


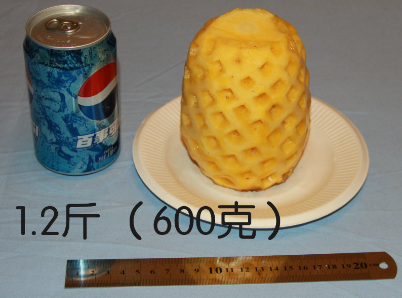
600g

(1) The frequency of consumption is:

□ Never □ 1 to 3 times per month □ Once a week □ 2 to 3 times a week

□ 4 to 5 times a week □ Once a day □ Twice a day □ Three times a day or more

(5)The amount of pineapples you consume each time is:

□ Less than or equal to 50 grams □ 50 to 150 grams □ 150 to 250 grams

□ 250 to 350 grams □ 350 to 450 grams □ 500 grams or more

1. **The situation of consuming mangoes**


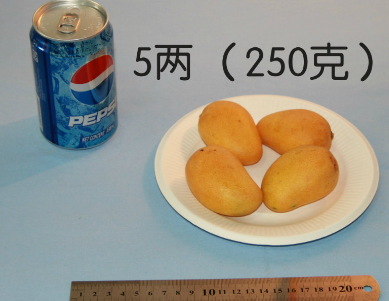
250g

(1) The frequency of consumption is:

□ Never □ 1 to 3 times per month □ Once a week □ 2 to 3 times a week

□ 4 to 5 times a week □ Once a day □ Twice a day □ Three times a day or more

(5)The amount of mangoes you consume each time is:

□ Less than or equal to 50 grams □ 50 to 150 grams □ 150 to 250 grams

□ 250 to 350 grams □ 350 to 450 grams □ 500 grams or more

1. **The situation of consuming kiwis**


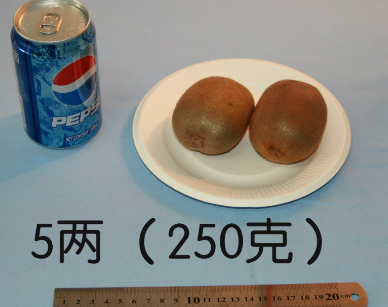
**250g**

(1) The frequency of consumption is:

□ Never □ 1 to 3 times per month □ Once a week □ 2 to 3 times a week

□ 4 to 5 times a week □ Once a day □ Twice a day □ Three times a day or more

(5)The amount of kiwis you consume each time is:

□ Less than or equal to 50 grams □ 50 to 150 grams □ 150 to 250 grams

□ 250 to 350 grams □ 350 to 450 grams □ 500 grams or more

- **The situation of consuming nuts and seeds**

**1. The situation of consuming dried walnuts**


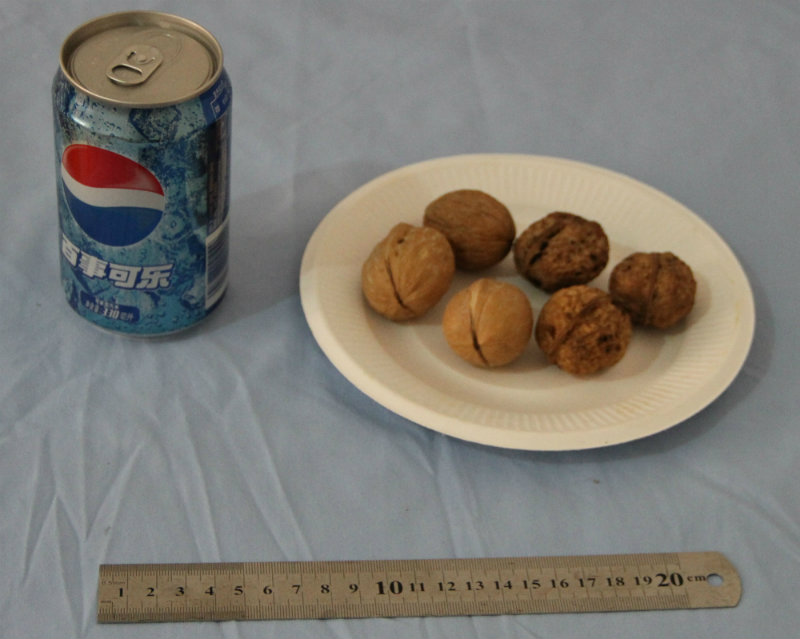
**50g**

(1) The frequency of consumption is:

□ Never □ 1 to 3 times per month □ Once a week □ 2 to 3 times a week

□ 4 to 5 times a week □ Once a day □ Twice a day □ Three times a day or more

(5)The amount of dried walnuts you consume each time is:

□ Less than or equal to 50 grams □ 50 to 150 grams □ 150 to 250 grams

□ 250 to 350 grams □ 350 to 450 grams □ 500 grams or more

**2. The situation of consuming chestnut**


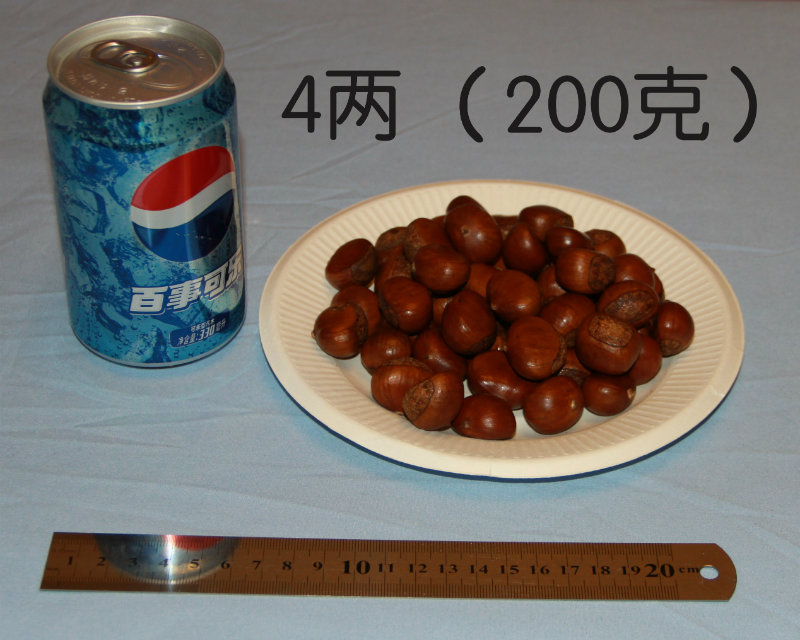
**200g**

(1) The frequency of consumption is:

□ Never □ 1 to 3 times per month □ Once a week □ 2 to 3 times a week

□ 4 to 5 times a week □ Once a day □ Twice a day □ Three times a day or more

(5)The amount of chestnuts you consume each time is:

□ Less than or equal to 50 grams □ 50 to 150 grams □ 150 to 250 grams

□ 250 to 350 grams □ 350 to 450 grams □ 500 grams or more

**3.The situation of consuming melon seeds**


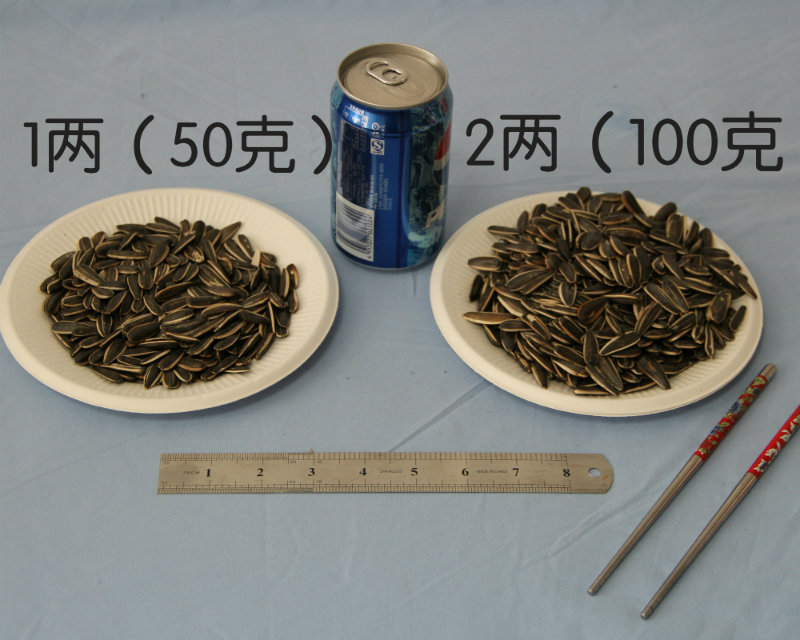


(1) The frequency of consumption is:

□ Never □ 1 to 3 times per month □ Once a week □ 2 to 3 times a week

□ 4 to 5 times a week □ Once a day □ Twice a day □ Three times a day or more

(5)The amount of melon seeds you consume each time is:

□ Less than or equal to 50 grams □ 50 to 150 grams □ 150 to 250 grams

□ 250 to 350 grams □ 350 to 450 grams □ 500 grams or more

**4. The situation of consuming pine nuts**


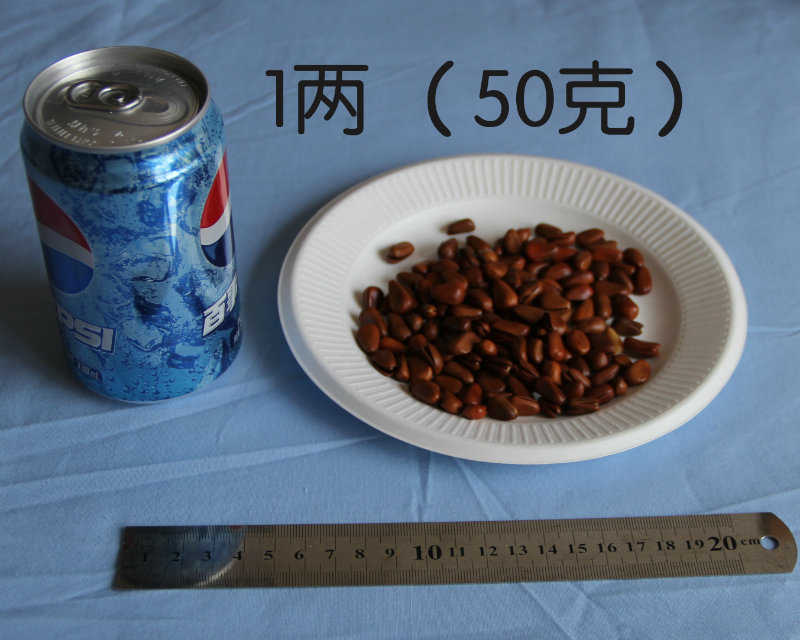
**50g**

(1) The frequency of consumption is:

□ Never □ 1 to 3 times per month □ Once a week □ 2 to 3 times a week

□ 4 to 5 times a week □ Once a day □ Twice a day □ Three times a day or more

(5)The amount of pine nuts you consume each time is:

□ Less than or equal to 50 grams □ 50 to 150 grams □ 150 to 250 grams

□ 250 to 350 grams □ 350 to 450 grams □ 500 grams or more

**5.The situation of consuming hazelnuts**


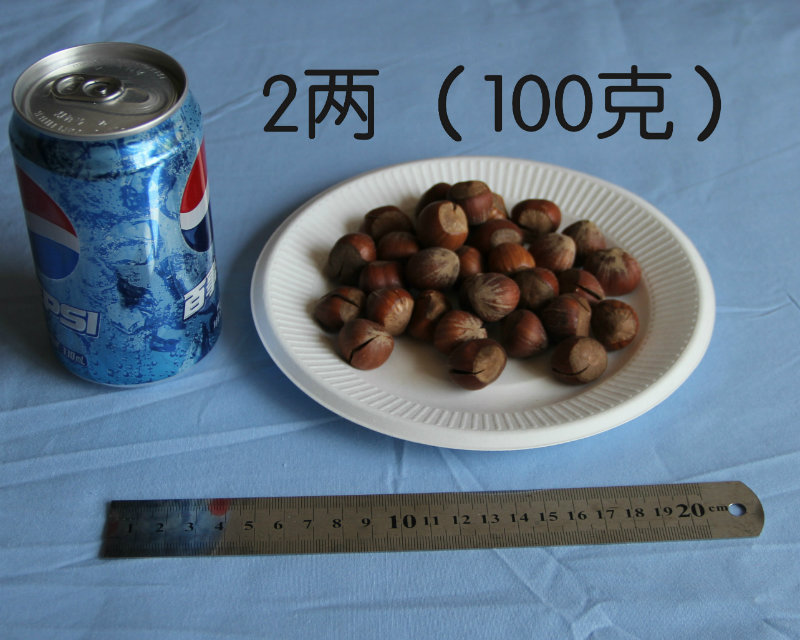
100g

(1) The frequency of consumption is:

□ Never □ 1 to 3 times per month □ Once a week □ 2 to 3 times a week

□ 4 to 5 times a week □ Once a day □ Twice a day □ Three times a day or more

(5)The amount of hazelnuts you consume each time is:

□ Less than or equal to 50 grams □ 50 to 150 grams □ 150 to 250 grams

□ 250 to 350 grams □ 350 to 450 grams □ 500 grams or more

**6.The situation of consuming cashew**


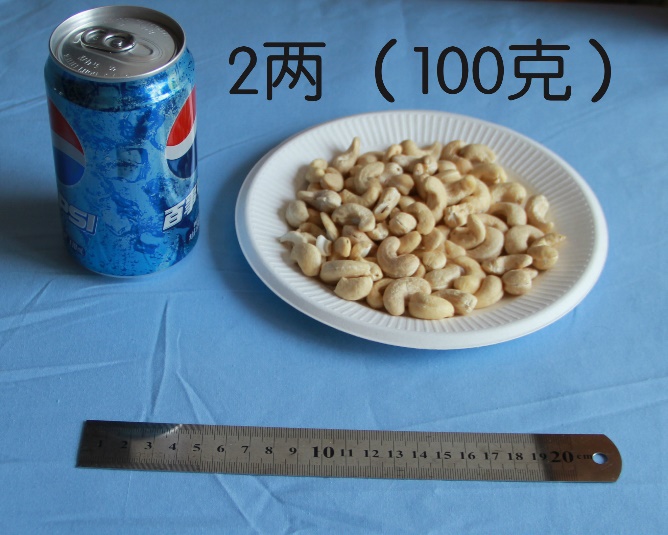
100g

(1) The frequency of consumption is:

□ Never □ 1 to 3 times per month □ Once a week □ 2 to 3 times a week

□ 4 to 5 times a week □ Once a day □ Twice a day □ Three times a day or more

(5)The amount of cashew you consume each time is:

□ Less than or equal to 50 grams □ 50 to 150 grams □ 150 to 250 grams

□ 250 to 350 grams □ 350 to 450 grams □ 500 grams or more

**7.The situation of consuming almond**


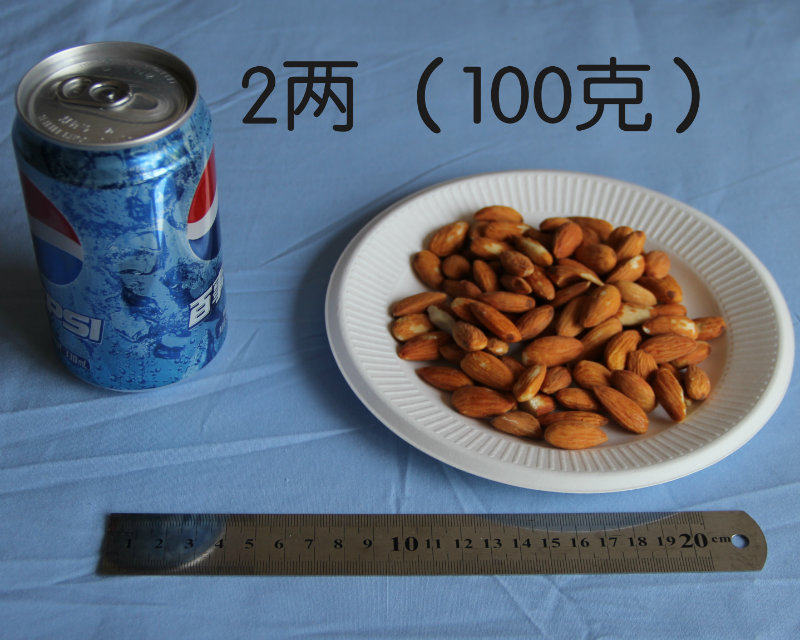
100g

(1) The frequency of consumption is:

□ Never □ 1 to 3 times per month □ Once a week □ 2 to 3 times a week

□ 4 to 5 times a week □ Once a day □ Twice a day □ Three times a day or more

(5)The amount of almond you consume each time is:

□ Less than or equal to 50 grams □ 50 to 150 grams □ 150 to 250 grams

□ 250 to 350 grams □ 350 to 450 grams □ 500 grams or more

**8.The situation of consuming peanut**


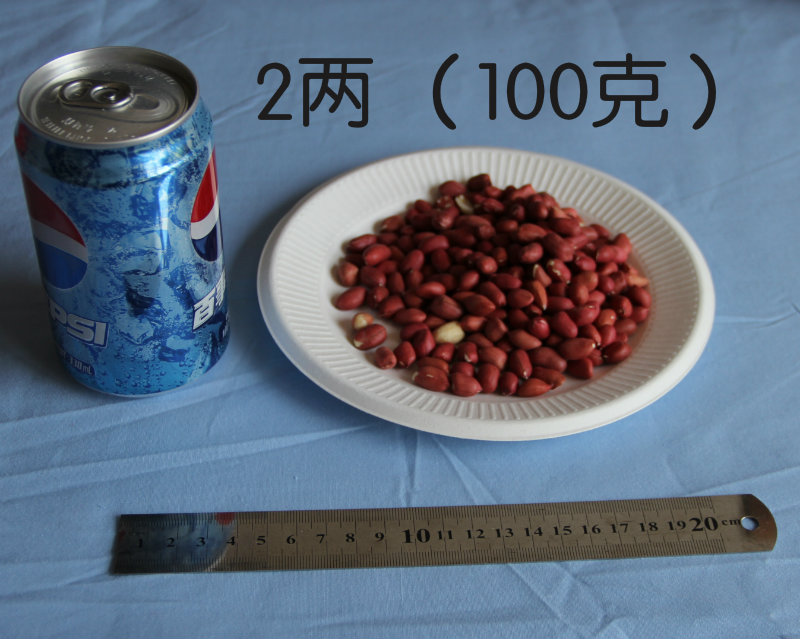
100g

(1) The frequency of consumption is:

□ Never □ 1 to 3 times per month □ Once a week □ 2 to 3 times a week

□ 4 to 5 times a week □ Once a day □ Twice a day □ Three times a day or more

(5)The amount of peanut you consume each time is:

□ Less than or equal to 50 grams □ 50 to 150 grams □ 150 to 250 grams

□ 250 to 350 grams □ 350 to 450 grams □ 500 grams or more

**9.The situation of consuming pistachio nut**


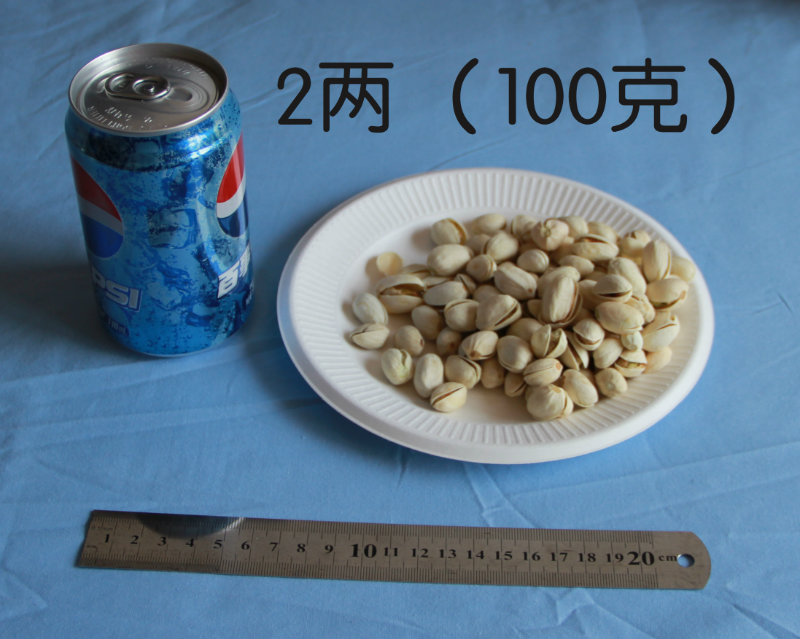
100g

(1) The frequency of consumption is:

□ Never □ 1 to 3 times per month □ Once a week □ 2 to 3 times a week

□ 4 to 5 times a week □ Once a day □ Twice a day □ Three times a day or more

(5)The amount of pistachio nut you consume each time is:

□ Less than or equal to 50 grams □ 50 to 150 grams □ 150 to 250 grams

□ 250 to 350 grams □ 350 to 450 grams □ 500 grams or more

- **The situation of consuming livestock and their food**

**1.The situation of consuming pork**


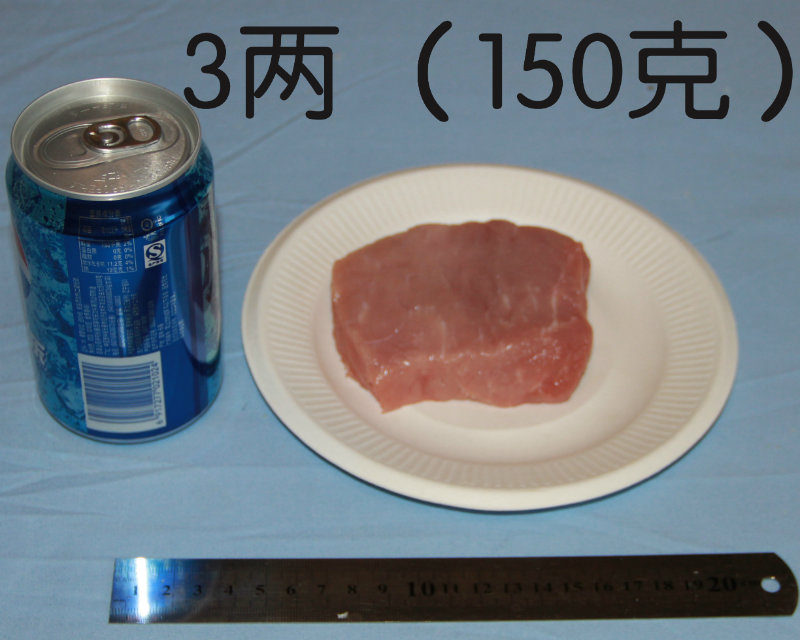
150g

(1) The frequency of consumption is:

□ Never □ 1 to 3 times per month □ Once a week □ 2 to 3 times a week

□ 4 to 5 times a week □ Once a day □ Twice a day □ Three times a day or more

(5)The amount of pork you consume each time is:

□ 50 grams □100 grams □ 150 grams □ 200 grams

□ 250 grams □ 300 grams □ 500 grams or more

**2.The situation of consuming liver of pig**

**
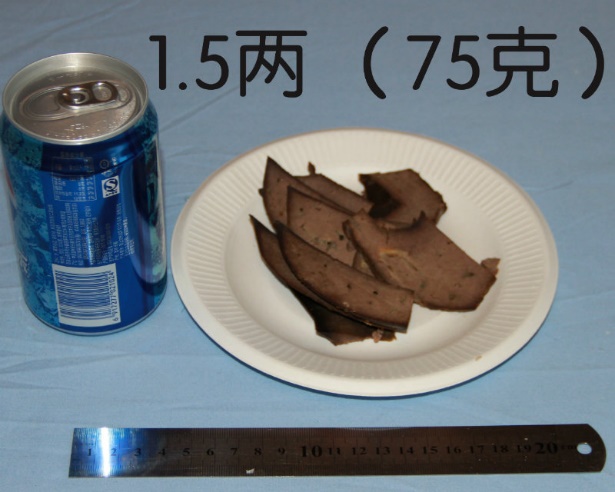
75g**

(1) The frequency of consumption is:

□ Never □ 1 to 3 times per month □ Once a week □ 2 to 3 times a week

□ 4 to 5 times a week □ Once a day □ Twice a day □ Three times a day or more

(5)The amount of liver of pig you consume each time is:

□ 50 grams □100 grams □ 150 grams □ 200 grams

□ 250 grams □ 300 grams □ 500 grams or more

**3. The situation of consuming beef and mutton**

**
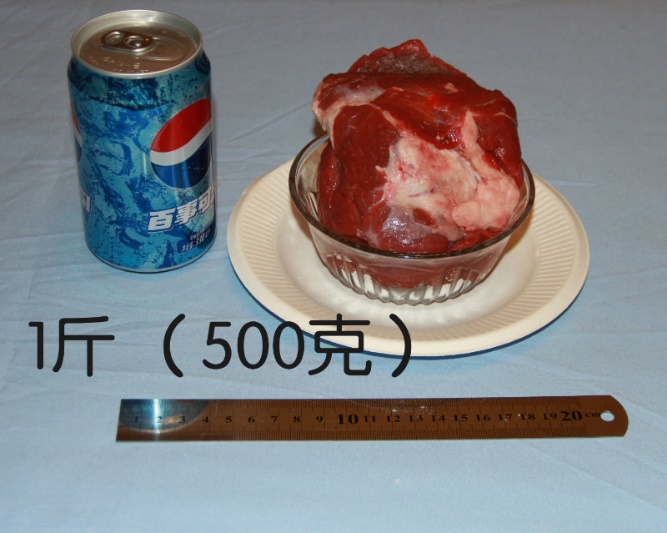
500g**

(1) The frequency of consumption is:

□ Never □ 1 to 3 times per month □ Once a week □ 2 to 3 times a week

□ 4 to 5 times a week □ Once a day □ Twice a day □ Three times a day or more

(5)The amount of beef and mutton you consume each time is:

□ 50 grams □100 grams □ 150 grams □ 200 grams

□ 250 grams □ 300 grams □ 500 grams or more

**4. The situation of consuming liver of beef and sheep**

**
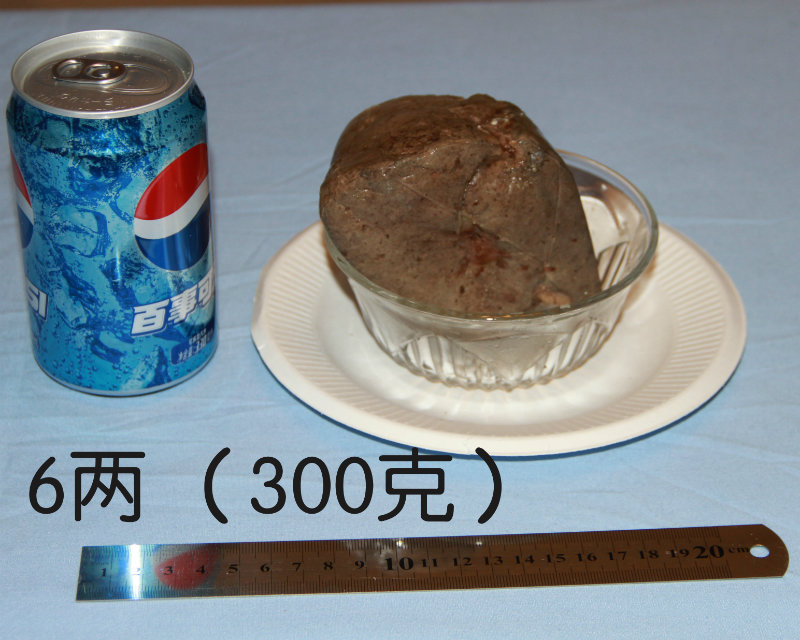
300g**

(1) The frequency of consumption is:

□ Never □ 1 to 3 times per month □ Once a week □ 2 to 3 times a week

□ 4 to 5 times a week □ Once a day □ Twice a day □ Three times a day or more

(5)The amount of liver of beef and sheep you consume each time is:

□ 50 grams □100 grams □ 150 grams □ 200 grams

□ 250 grams □ 300 grams □ 500 grams or more

**5.The situation of consuming sausage and ham sausage**

**
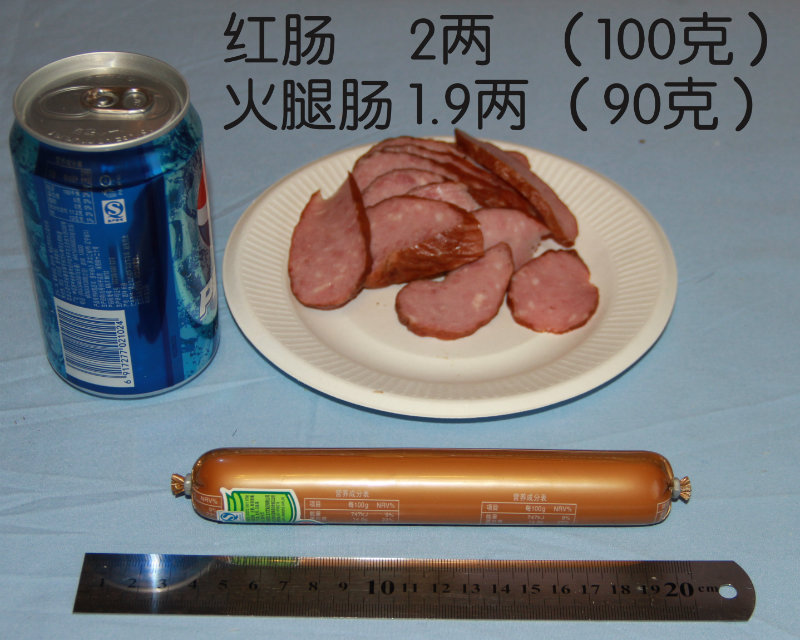
**

(1) The frequency of consumption is:

□ Never □ 1 to 3 times per month □ Once a week □ 2 to 3 times a week

□ 4 to 5 times a week □ Once a day □ Twice a day □ Three times a day or more

(5)The amount of sausage and ham sausage you consume each time is:

□ 50 grams □100 grams □ 150 grams □ 200 grams

□ 250 grams □ 300 grams □ 500 grams or more

- **The situation of consuming poultry and their food**

**1.The situation of consuming chicken**

(1) The frequency of consumption is:

□ Never □ 1 to 3 times per month □ Once a week □ 2 to 3 times a week

□ 4 to 5 times a week □ Once a day □ Twice a day □ Three times a day or more

(5)The amount of chicken you consume each time is:

□ 50 grams □100 grams □ 150 grams □ 200 grams

□ 250 grams □ 300 grams □ 500 grams or more

**2.The situation of consuming chicken liver**

(1) The frequency of consumption is:

□ Never □ 1 to 3 times per month □ Once a week □ 2 to 3 times a week

□ 4 to 5 times a week □ Once a day □ Twice a day □ Three times a day or more

(5)The amount of chicken liver you consume each time is:

□ 50 grams □100 grams □ 150 grams □ 200 grams

□ 250 grams □ 300 grams □ 500 grams or more

**3.The situation of consuming duck and goose**

**
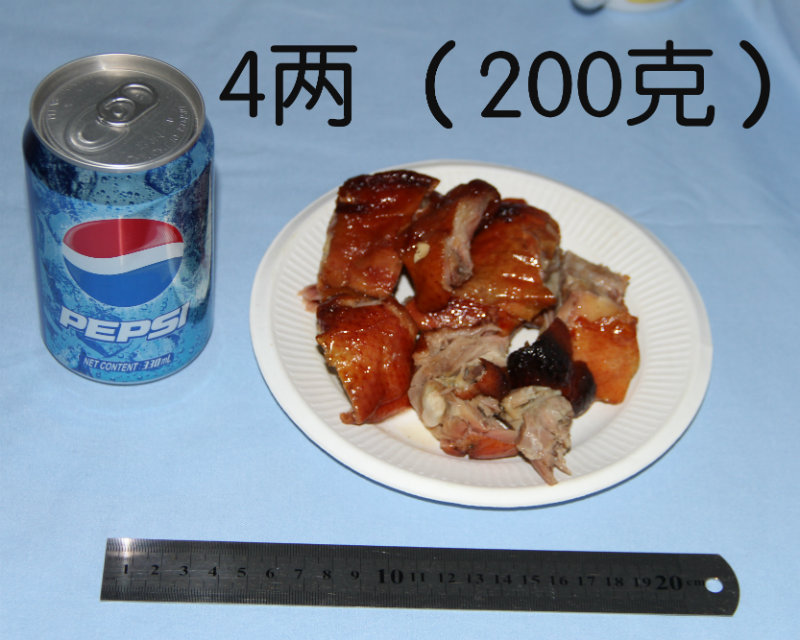
200g**

(1) The frequency of consumption is:

□ Never □ 1 to 3 times per month □ Once a week □ 2 to 3 times a week

□ 4 to 5 times a week □ Once a day □ Twice a day □ Three times a day or more

(5)The amount of duck and goose you consume each time is:

□ 50 grams □100 grams □ 150 grams □ 200 grams

□ 250 grams □ 300 grams □ 500 grams or more

**4.The situation of consuming fried chicken**

**
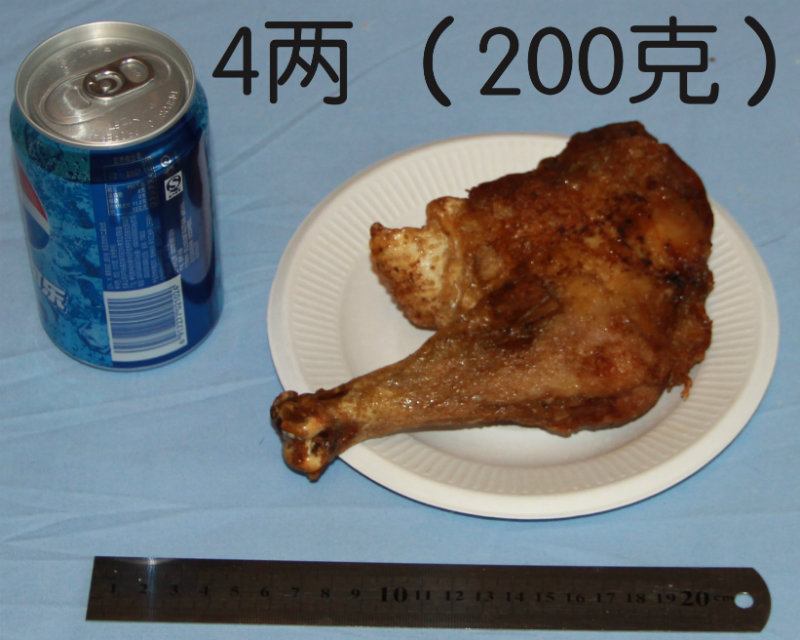
200g**

(1) The frequency of consumption is:

□ Never □ 1 to 3 times per month □ Once a week □ 2 to 3 times a week

□ 4 to 5 times a week □ Once a day □ Twice a day □ Three times a day or more

(5)The amount of fried chicken you consume each time is:

□ 50 grams □100 grams □ 150 grams □ 200 grams

□ 250 grams □ 300 grams □ 500 grams or more

- **The situation of consuming dairy and its products**

**1.The situation of consuming powdered milk**

**
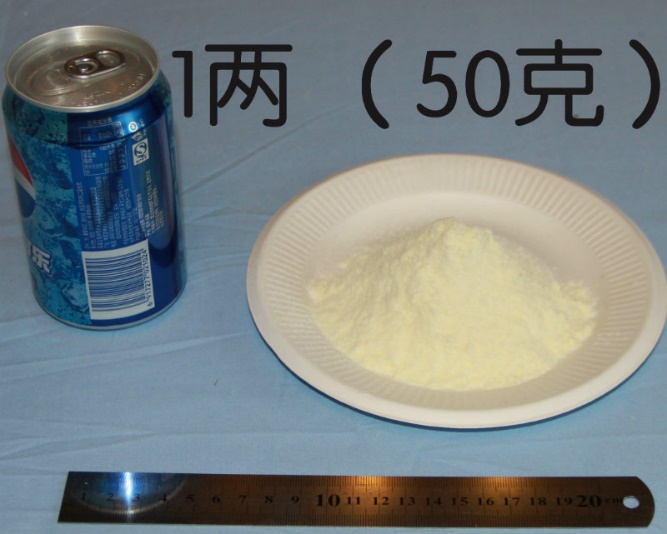
50g**

(1) The frequency of consumption is:

□ Never □ 1 to 3 times per month □ Once a week □ 2 to 3 times a week

□ 4 to 5 times a week □ Once a day □ Twice a day □ Three times a day or more

(5)The amount of powdered milk you consume each time is:

□ Less than or equal to 25 grams □ 25 to 50 grams □ 50 to 75 grams

□ 75 to 80 grams □ 80 to 100 grams □ 100 grams or more

**2.The situation of consuming fresh milk**

**
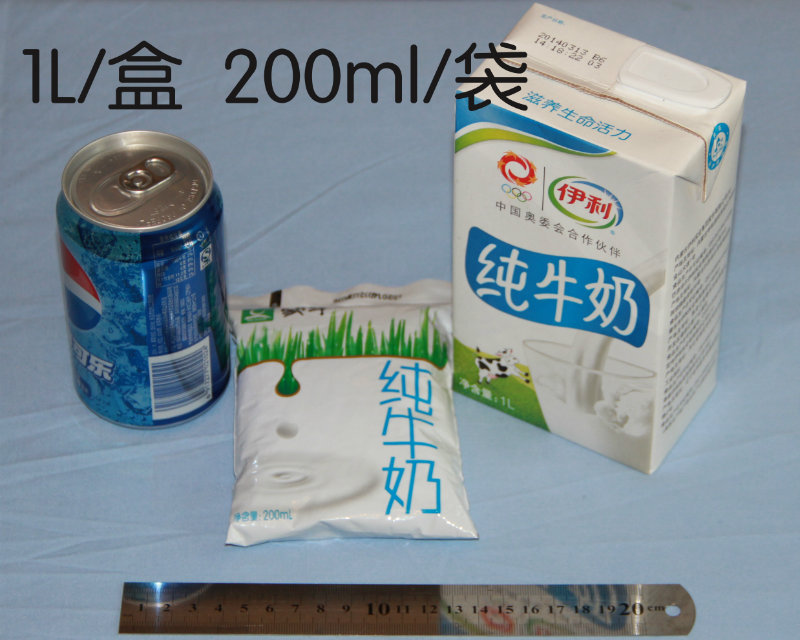
**

(1) The frequency of consumption is:

□ Never □ 1 to 3 times per month □ Once a week □ 2 to 3 times a week

□ 4 to 5 times a week □ Once a day □ Twice a day □ Three times a day or more

(5)The amount of fresh milk you consume each time is:

□ Less than or equal to 100ml □ 100ml to 150ml □ 200ml to 250ml (a bag)

□ 300ml to 350ml □ 400 to 450ml □ 500ml or more ( a small bag is normally 221ml)

**3. .The situation of consuming yoghurt**


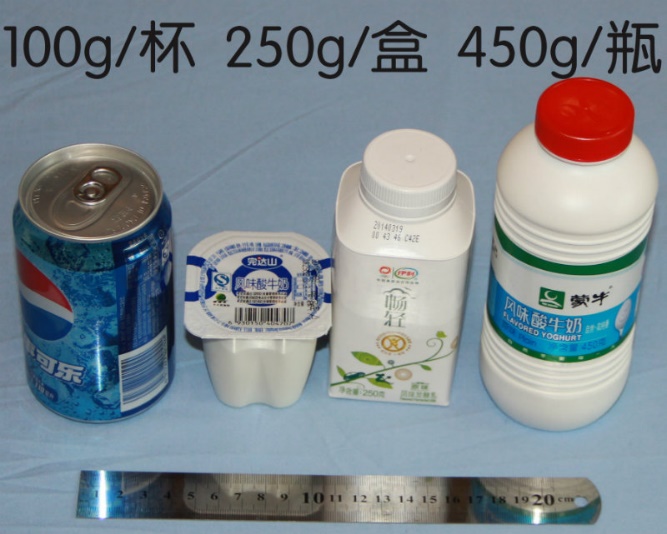


(1) The frequency of consumption is:

□ Never □ 1 to 3 times per month □ Once a week □ 2 to 3 times a week

□ 4 to 5 times a week □ Once a day □ Twice a day □ Three times a day or more

(5)The amount of yoghurt you consume each time is:

□ Less than or equal to 100ml □ 100ml to 150ml（a glass） □ 200ml to 250ml (a bottle)

□ 300ml to 350ml □ 400 to 450ml □ 500ml or more

- **The situation of consuming eggs and their food products**

**1.The situation of consuming eggs**

**
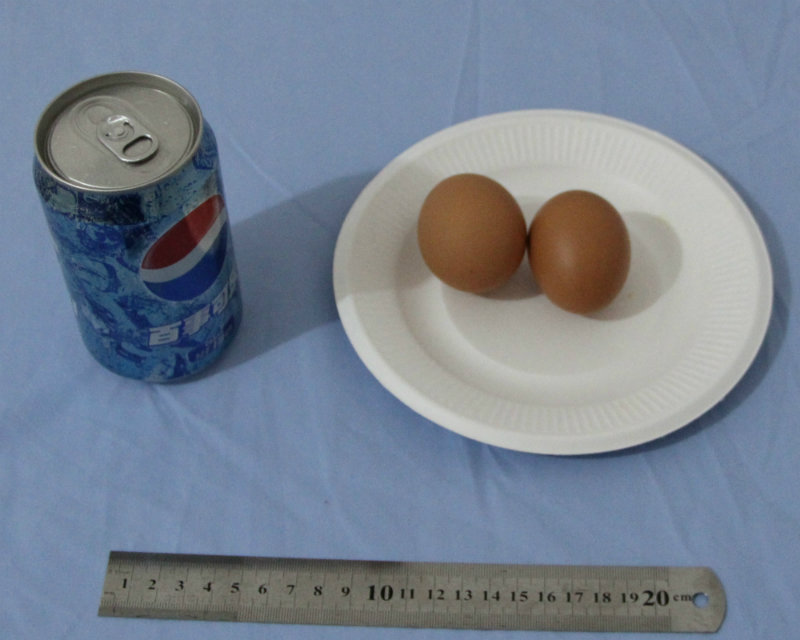
**

(1) The frequency of consumption is:

□ Never □ 1 to 3 times per month □ Once a week □ 2 to 3 times a week

□ 4 to 5 times a week □ Once a day □ Twice a day □ Three times a day or more

(5)The amount of eggs you consume each time is:

□ Less than or equal to one □ Two □ Three

□Four □ Five □ Six □ Six or more

**2.The situation of consuming duck eggs**

**
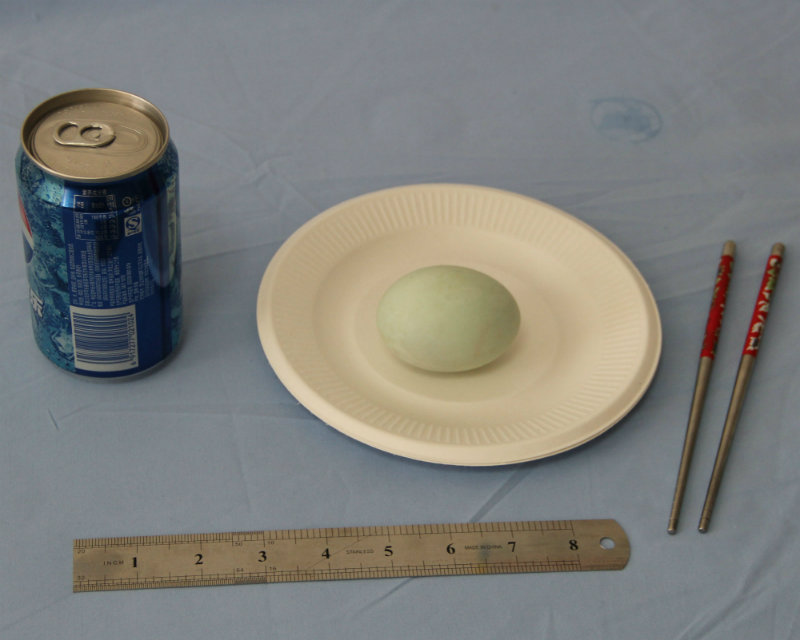
**

(1) The frequency of consumption is:

□ Never □ 1 to 3 times per month □ Once a week □ 2 to 3 times a week

□ 4 to 5 times a week □ Once a day □ Twice a day □ Three times a day or more

(5)The amount of duck eggs you consume each time is:

□ Less than or equal to one □ Two □ Three

□Four □ Five □ Six □ Six or more

**3.The situation of consuming preserved duck eggs**

**
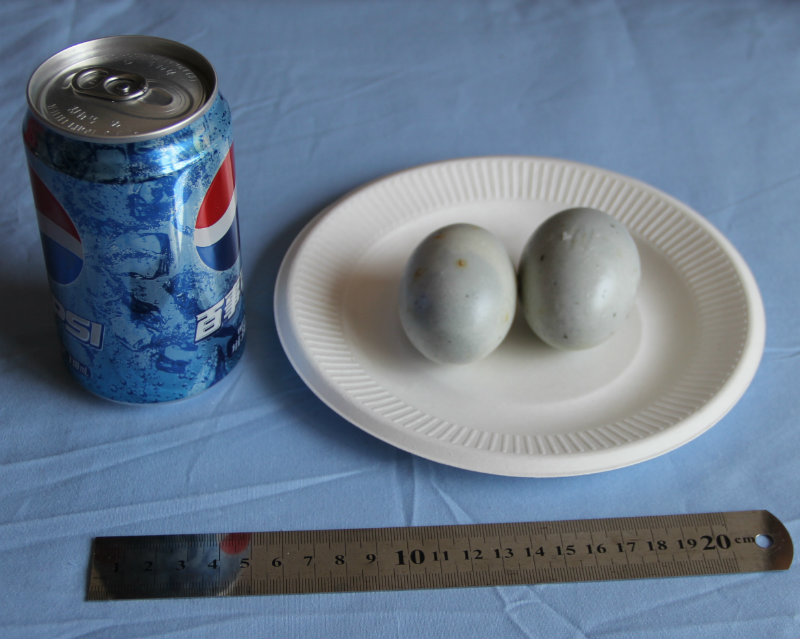
**

(1) The frequency of consumption is:

□ Never □ 1 to 3 times per month □ Once a week □ 2 to 3 times a week

□ 4 to 5 times a week □ Once a day □ Twice a day □ Three times a day or more

(5)The amount of preserved duck eggs you consume each time is:

□ Less than or equal to one □ Two □ Three

□Four □ Five □ Six □ Six or more

**4.The situation of consuming goose eggs**

(1) The frequency of consumption is:

□ Never □ 1 to 3 times per month □ Once a week □ 2 to 3 times a week

□ 4 to 5 times a week □ Once a day □ Twice a day □ Three times a day or more

(5)The amount of goose eggs you consume each time is:

□ Less than or equal to one □ Two □ Three

□Four □ Five □ Six □ Six or more

**4.The situation of consuming quail eggs**


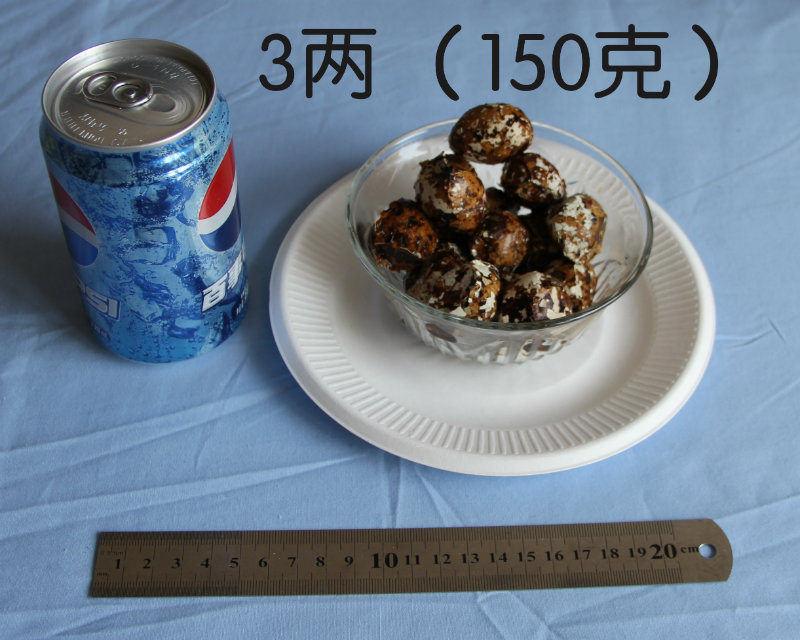
150g

(1) The frequency of consumption is:

□ Never □ 1 to 3 times per month □ Once a week □ 2 to 3 times a week

□ 4 to 5 times a week □ Once a day □ Twice a day □ Three times a day or more

(5)The amount of quail eggs you consume each time is:

□ Less than or equal to one □ Two □ Three

□Four □ Five □ Six □ Six or more

- **The situation of consuming fish, shrimp, crabs and shellfish**

**1.The situation of consuming fish**


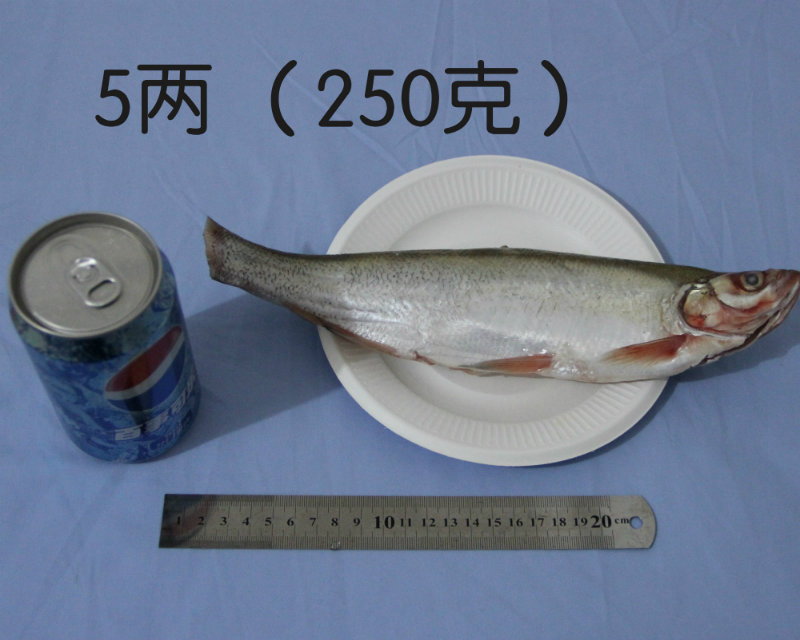
250g

(1) The frequency of consumption is:

□ Never □ 1 to 3 times per month □ Once a week □ 2 to 3 times a week □ 4 to 5 times a week □ Once a day □ Twice a day □ Three times a day or more

(5)The amount of fish you consume each time is:

□ Less than or equal to 50 grams □ 50 to 150 grams □ 200 to 250 grams

□ 300 to 350 grams □ 400 to 450 grams □ 500 grams or more

**2.The situation of consuming shrimp**


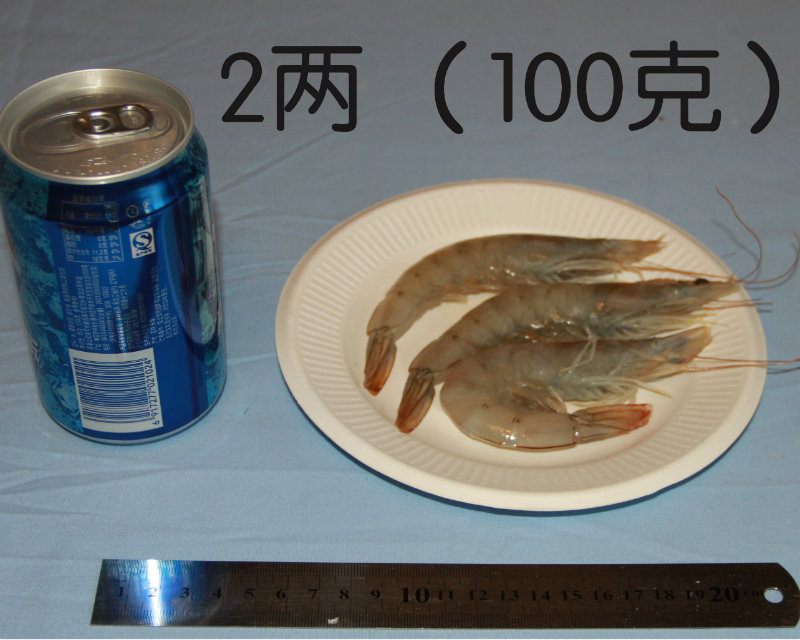
100g

(1) The frequency of consumption is:

□ Never □ 1 to 3 times per month □ Once a week □ 2 to 3 times a week □ 4 to 5 times a week □ Once a day □ Twice a day □ Three times a day or more

(5)The amount of shrimp you consume each time is:

□ Less than or equal to 50 grams □ 50 to 150 grams □ 200 to 250 grams

□ 300 to 350 grams □ 400 to 450 grams □ 500 grams or more

**3.The situation of consuming dried shrimp**


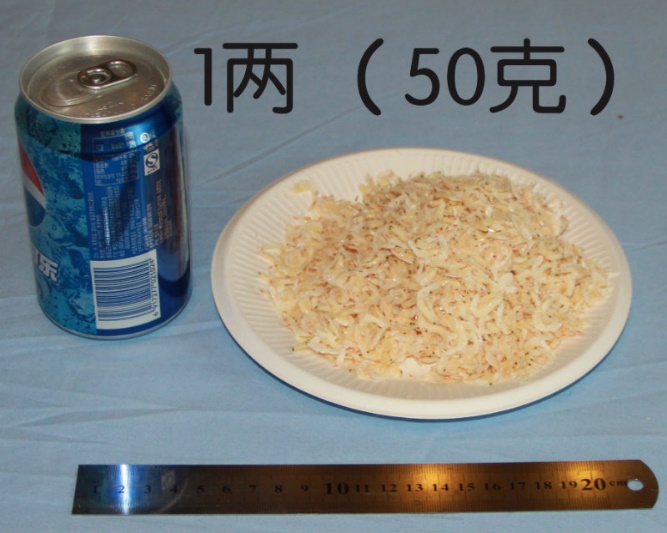
50g

(1) The frequency of consumption is:

□ Never □ 1 to 3 times per month □ Once a week □ 2 to 3 times a week □ 4 to 5 times a week □ Once a day □ Twice a day □ Three times a day or more

(5)The amount of dried shrimp you consume each time is:

□ Less than or equal to 50 grams □ 50 to 150 grams □ 200 to 250 grams

□ 300 to 350 grams □ 400 to 450 grams □ 500 grams or more

**4.The situation of consuming crab**

(1) The frequency of consumption is:

□ Never □ 1 to 3 times per month □ Once a week □ 2 to 3 times a week □ 4 to 5 times a week □ Once a day □ Twice a day □ Three times a day or more

(5)The amount of crab you consume each time is:

□ Less than or equal to 50 grams □ 50 to 150 grams □ 200 to 250 grams

□ 300 to 350 grams □ 400 to 450 grams □ 500 grams or more

**5.The situation of consuming squid**

(1) The frequency of consumption is:

□ Never □ 1 to 3 times per month □ Once a week □ 2 to 3 times a week □ 4 to 5 times a week □ Once a day □ Twice a day □ Three times a day or more

(5)The amount of squid you consume each time is:

□ Less than or equal to 50 grams □ 50 to 150 grams □ 200 to 250 grams

□ 300 to 350 grams □ 400 to 450 grams □ 500 grams or more

**6.The situation of consuming shellfish**


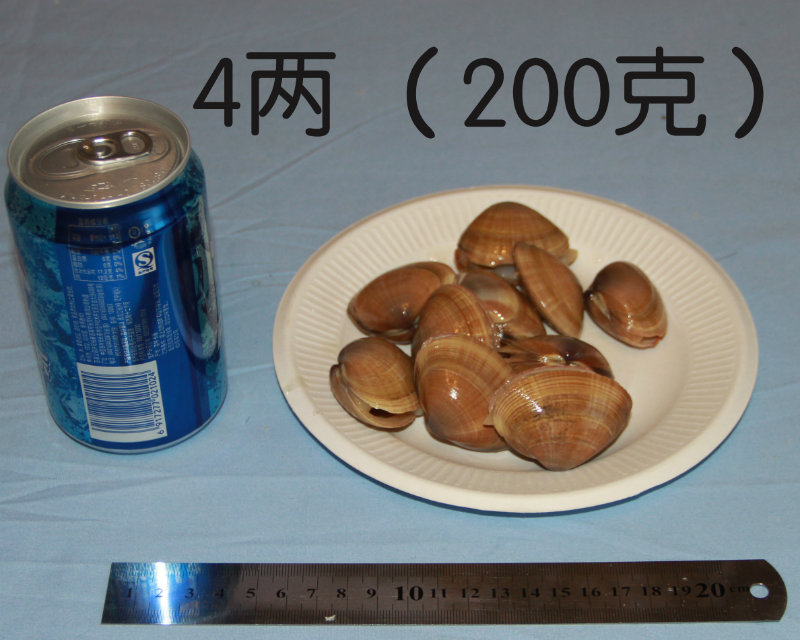
200g

(1) The frequency of consumption is:

□ Never □ 1 to 3 times per month □ Once a week □ 2 to 3 times a week □ 4 to 5 times a week □ Once a day □ Twice a day □ Three times a day or more

(5)The amount of shellfish you consume each time is:

□ Less than or equal to 50 grams □ 50 to 150 grams □ 200 to 250 grams

□ 300 to 350 grams □ 400 to 450 grams □ 500 grams or more

**7.The situation of consuming fresh jellyfish**

(1) The frequency of consumption is:

□ Never □ 1 to 3 times per month □ Once a week □ 2 to 3 times a week □ 4 to 5 times a week □ Once a day □ Twice a day □ Three times a day or more

(5)The amount of fresh jellyfish you consume each time is:

□ Less than or equal to 50 grams □ 50 to 150 grams □ 200 to 250 grams

□ 300 to 350 grams □ 400 to 450 grams □ 500 grams or more

- **The situation of consuming fast food**

**1.The situation of consuming pastry**


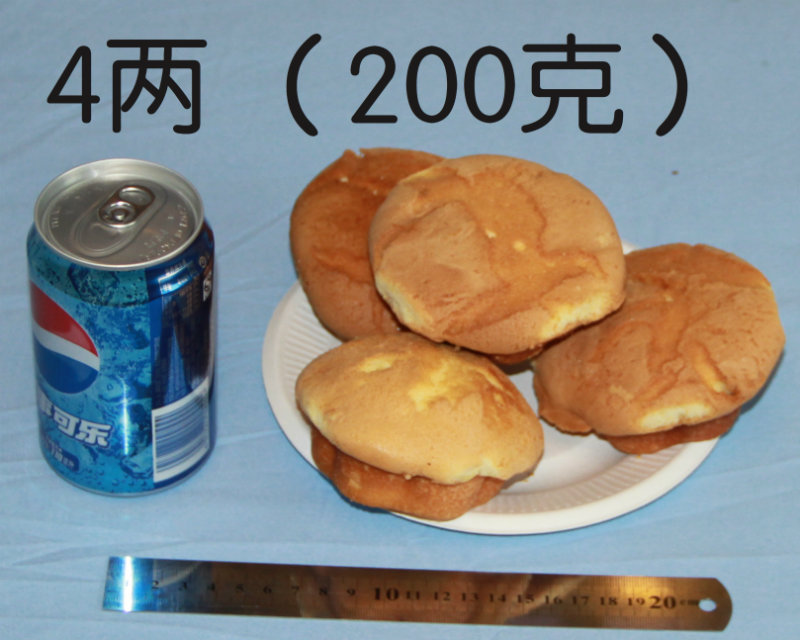
200g

(1) The frequency of consumption is:

□ Never □ 1 to 3 times per month □ Once a week □ 2 to 3 times a week □ 4 to 5 times a week □ Once a day □ Twice a day □ Three times a day or more

(5)The amount of pastry you consume each time is:

□ Less than or equal to 50 grams □ 50 to 150 grams □ 200 to 250 grams

□ 300 to 350 grams □ 400 to 450 grams □ 500 grams or more

**2.The situation of consuming instant noodles**


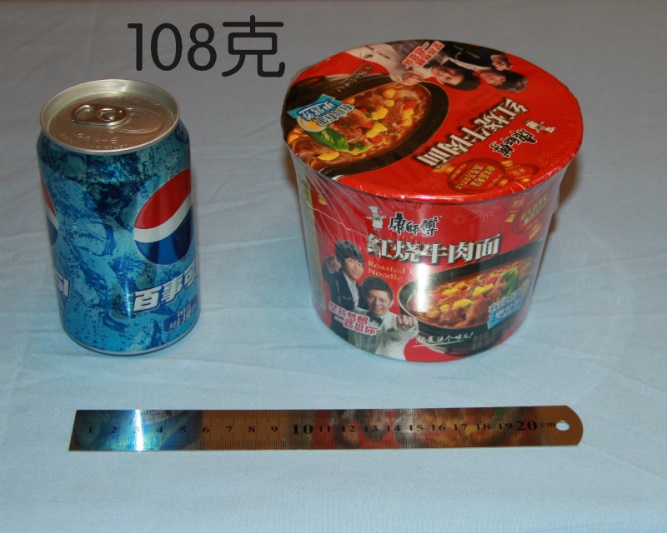
108g

(1) The frequency of consumption is:

□ Never □ 1 to 3 times per month □ Once a week □ 2 to 3 times a week □ 4 to 5 times a week □ Once a day □ Twice a day □ Three times a day or more

(5)The amount of instant noodles you consume each time is:

□ Less than or equal to 50 grams □ 50 to 150 grams □ 200 to 250 grams

□ 300 to 350 grams □ 400 to 450 grams □ 500 grams or more

**3.The situation of consuming bread**


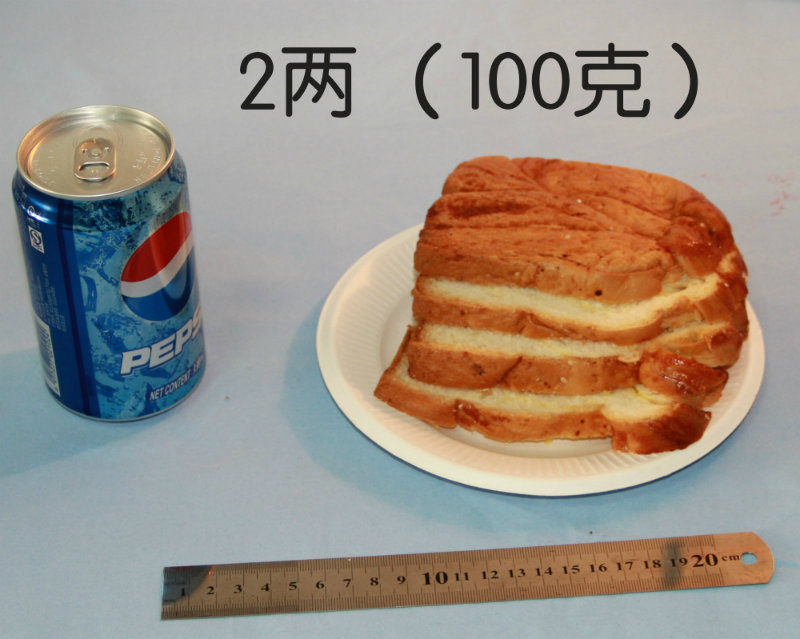
100g

(1) The frequency of consumption is:

□ Never □ 1 to 3 times per month □ Once a week □ 2 to 3 times a week □ 4 to 5 times a week □ Once a day □ Twice a day □ Three times a day or more

(5)The amount of bread you consume each time is:

□ Less than or equal to 50 grams □ 50 to 150 grams □ 200 to 250 grams

□ 300 to 350 grams □ 400 to 450 grams □ 500 grams or more

**4.The situation of consuming cornmeal**


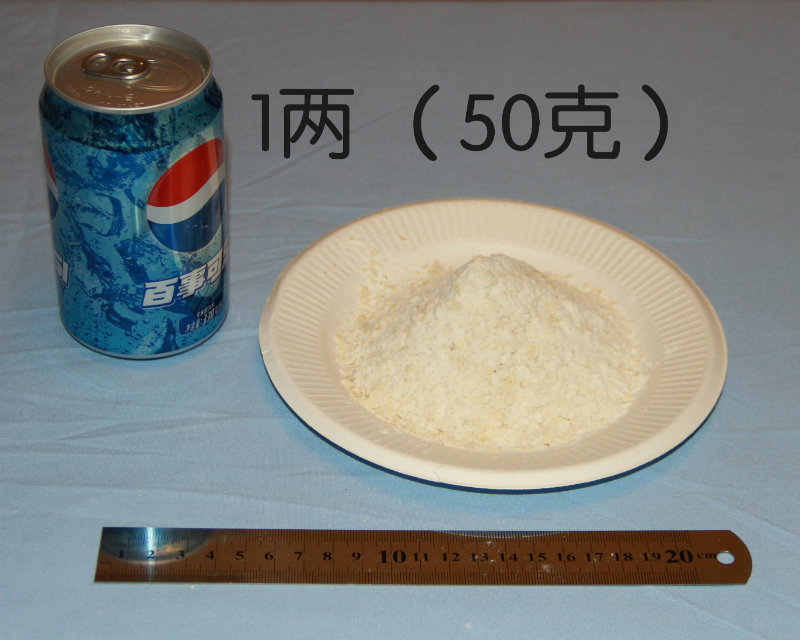
50g

(1) The frequency of consumption is:

□ Never □ 1 to 3 times per month □ Once a week □ 2 to 3 times a week □ 4 to 5 times a week □ Once a day □ Twice a day □ Three times a day or more

(5)The amount of cornmeal you consume each time is:

□ Less than or equal to 50 grams □ 50 to 150 grams □ 200 to 250 grams

□ 300 to 350 grams □ 400 to 450 grams □ 500 grams or more

**5.The situation of consuming biscuit**

**
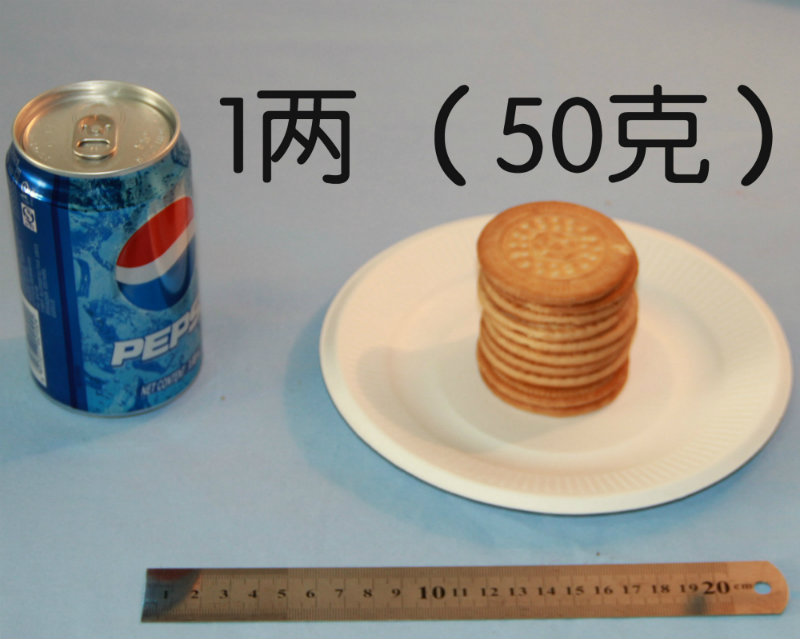
**50g

(1) The frequency of consumption is:

□ Never □ 1 to 3 times per month □ Once a week □ 2 to 3 times a week □ 4 to 5 times a week □ Once a day □ Twice a day □ Three times a day or more

(5)The amount of biscuit you consume each time is:

□ Less than or equal to 50 grams □ 50 to 150 grams □ 200 to 250 grams

□ 300 to 350 grams □ 400 to 450 grams □ 500 grams or more

**6.The situation of consuming potato chips**


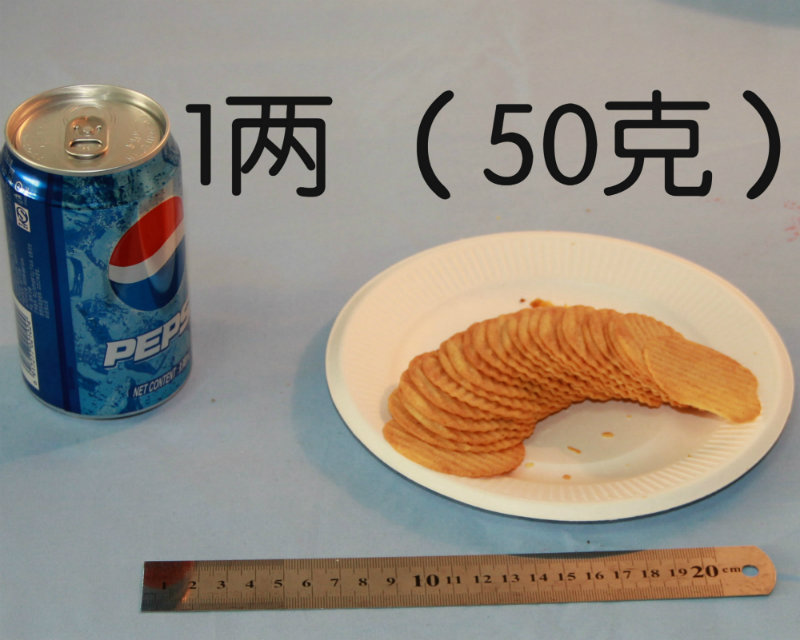
50g

(1) The frequency of consumption is:

□ Never □ 1 to 3 times per month □ Once a week □ 2 to 3 times a week □ 4 to 5 times a week □ Once a day □ Twice a day □ Three times a day or more

(5)The amount of potato chips you consume each time is:

□ Less than or equal to 50 grams □ 50 to 150 grams □ 200 to 250 grams

□ 300 to 350 grams □ 400 to 450 grams □ 500 grams or more

- **The situation of consuming sugar and preserves**

**1.The situation of consuming candy**

**
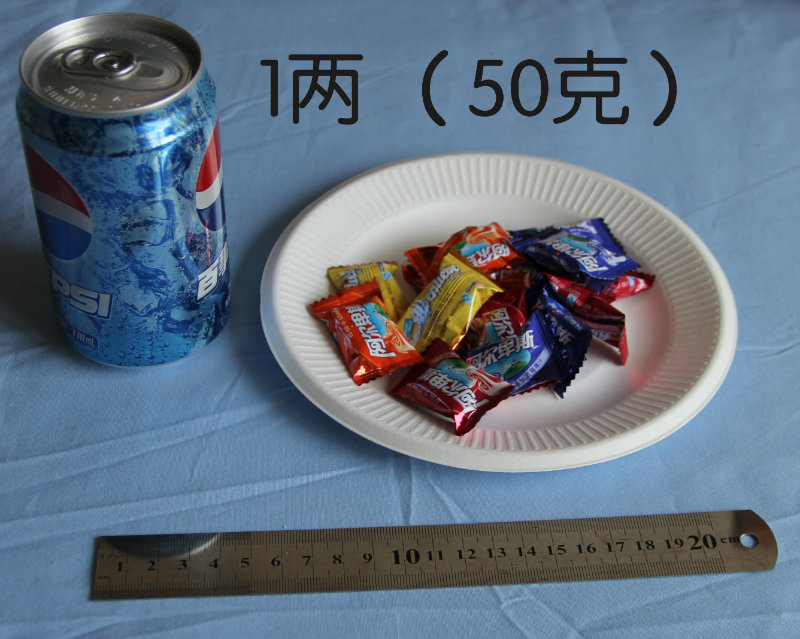
50g**

(1) The frequency of consumption is:

□ Never □ 1 to 3 times per month □ Once a week □ 2 to 3 times a week □ 4 to 5 times a week □ Once a day □ Twice a day □ Three times a day or more

(5)The amount of candy you consume each time is:

□ Less than or equal to 10 grams □ 20 to 30 grams □ 40 to 50 grams

□ 60 to 70 grams □ 80 to 90 grams □ 100 grams or more

**2.The situation of consuming preserved fruit**

(1) The frequency of consumption is:

□ Never □ 1 to 3 times per month □ Once a week □ 2 to 3 times a week □ 4 to 5 times a week □ Once a day □ Twice a day □ Three times a day or more

(5)The amount of preserved fruit you consume each time is:

□ Less than or equal to 10 grams □ 20 to 30 grams □ 40 to 50 grams

□ 60 to 70 grams □ 80 to 90 grams □ 100 grams or more

**3.The situation of consuming chocolate**


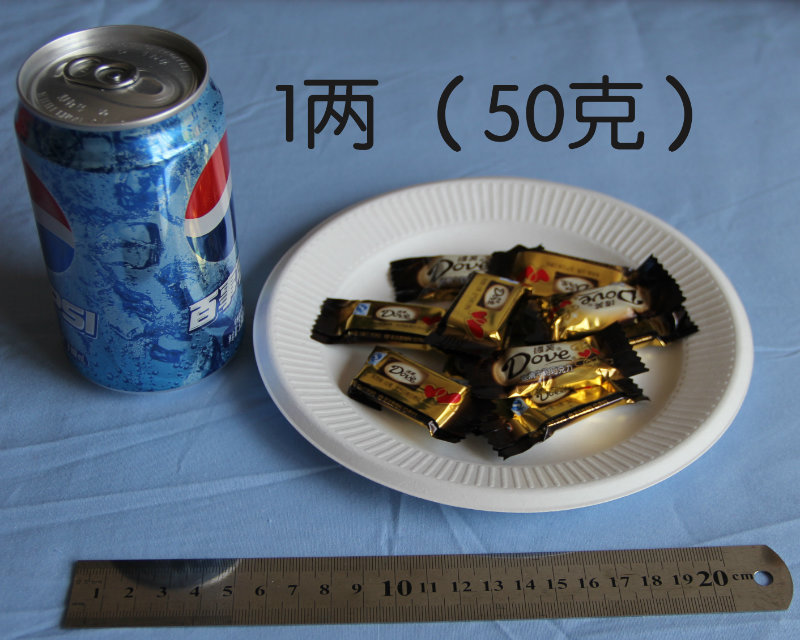
50g

(1) The frequency of consumption is:

□ Never □ 1 to 3 times per month □ Once a week □ 2 to 3 times a week □ 4 to 5 times a week □ Once a day □ Twice a day □ Three times a day or more

(5)The amount of chocolate you consume each time is:

□ Less than or equal to 10 grams □ 20 to 30 grams □ 40 to 50 grams

□ 60 to 70 grams □ 80 to 90 grams □ 100 grams or more

**4.The situation of consuming honey**


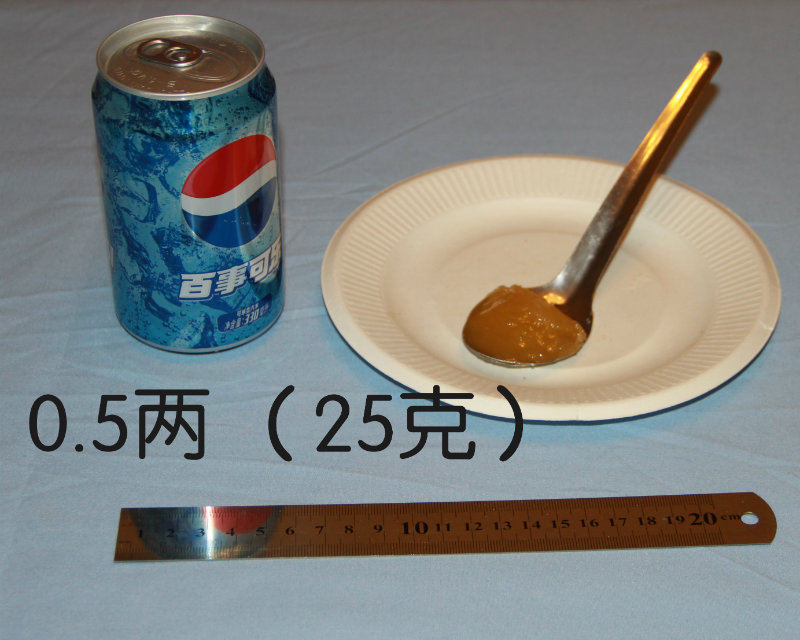


(1) The frequency of consumption is:

□ Never □ 1 to 3 times per month □ Once a week □ 2 to 3 times a week □ 4 to 5 times a week □ Once a day □ Twice a day □ Three times a day or more

(5)The amount of honey you consume each time is:

□ Less than or equal to 2 grams（less than half a spoon） □ 3 to 5 grams(half a spoon)

□ 6 to 8 grams(more than half a spoon) □ 9 to 10 grams (a spoon)

□ 11 to 14 grams (one and a half spoons or more) □ 15 to 20 grams

- **The situation of consuming beverage**

**1.The situation of consuming sweet tea beverage**


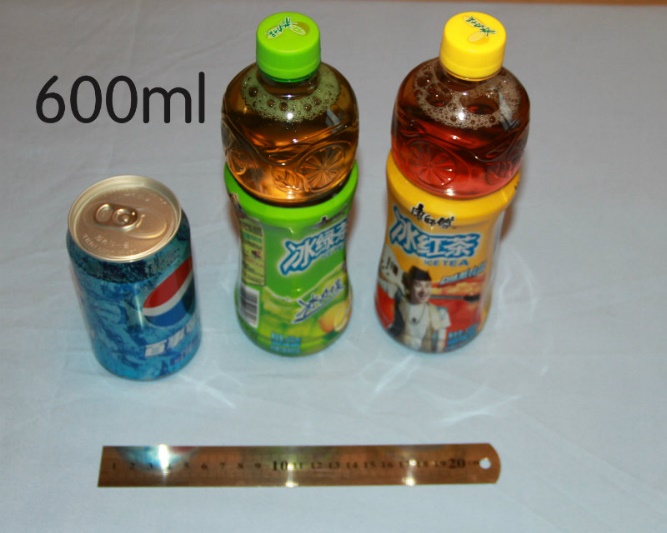
600ml

(1) The frequency of consumption is:

□ Never □ 1 to 3 times per month □ Once a week □ 2 to 3 times a week □ 4 to 5 times a week □ Once a day □ Twice a day □ Three times a day or more

(5)The amount of sweet tea beverage you consume each time is:

□ Less than or equal to 100ml □ 100ml to 150 ml □ 200ml to 250 ml

□ 300ml to 350 ml □ 400ml to 450 ml □ 500 ml or more(about a bottle)

**2.The situation of consuming sweetened carbonated beverage (Pepsi, Coca-Cola, etc.)**


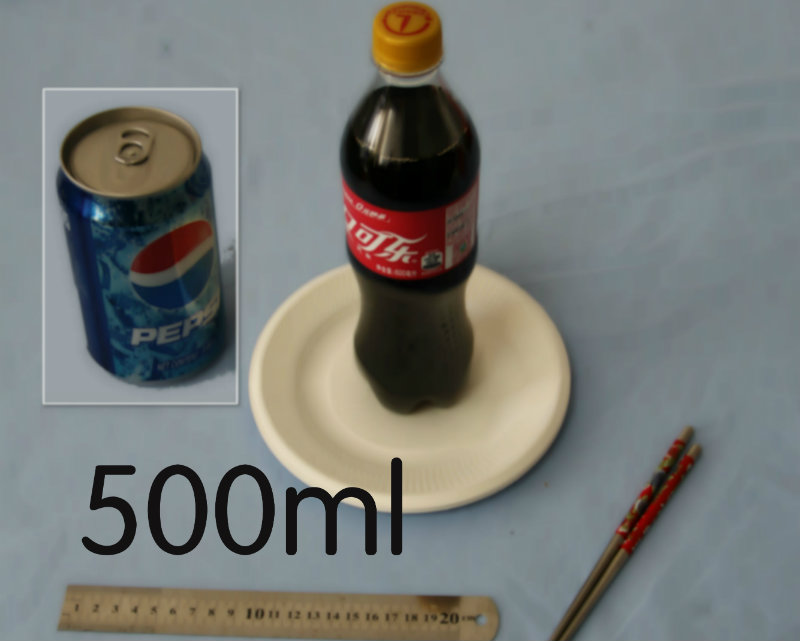
500ml

(1) The frequency of consumption is:

□ Never □ 1 to 3 times per month □ Once a week □ 2 to 3 times a week □ 4 to 5 times a week □ Once a day □ Twice a day □ Three times a day or more

(5)The amount of sweetened carbonated beverage you consume each time is:

□ Less than or equal to 100ml □ 100ml to 150 ml □ 200ml to 250 ml

□ 300ml to 350 ml □ 400ml to 450 ml □ 500 ml or more(about a bottle)

**3.The situation of consuming sweet fruit juice**


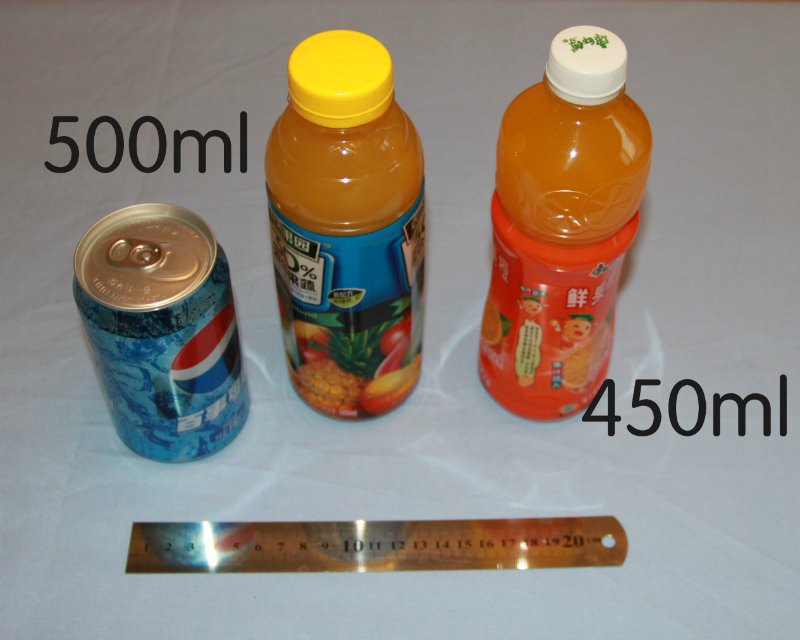


(1) The frequency of consumption is:

□ Never □ 1 to 3 times per month □ Once a week □ 2 to 3 times a week □ 4 to 5 times a week □ Once a day □ Twice a day □ Three times a day or more

(5)The amount of sweet fruit juice you consume each time is:

□ Less than or equal to 100ml □ 100ml to 150 ml □ 200ml to 250 ml

□ 300ml to 350 ml □ 400ml to 450 ml □ 500 ml or more(about a bottle)

- **The situation of condiment**

**1.The situation of consuming tahini**


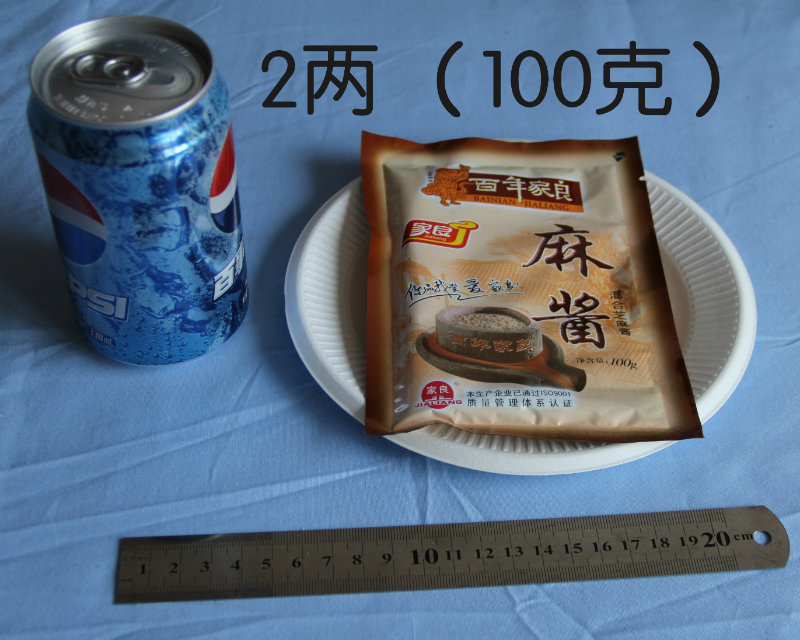
100g

(1) The frequency of consumption is:

□ Never □ 1 to 3 times per month □ Once a week □ 2 to 3 times a week

□ 4 to 5 times a week □ Once a day □ Twice a day □ Three times a day or more

(5)The amount of tahini you consume each time is:

□ Less than or equal to 5 grams □ 10 to 15 grams □ 20 to 25 grams

□ 30 to 35 grams □ 40 to 45 grams □ 50 grams or more

**2.The situation of consuming broad bean paste (ordinary household sauce)**


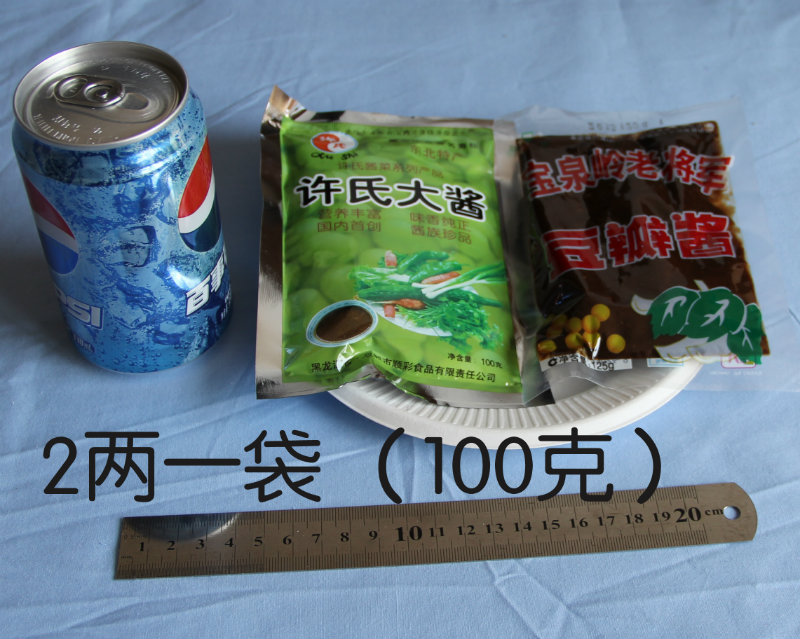
100g

(1) The frequency of consumption is:

□ Never □ 1 to 3 times per month □ Once a week □ 2 to 3 times a week

□ 4 to 5 times a week □ Once a day □ Twice a day □ Three times a day or more

(5)The amount of broad bean paste you consume each time is:

□ Less than or equal to 5 grams □ 10 to 15 grams □ 20 to 25 grams

□ 30 to 35 grams □ 40 to 45 grams □ 50 grams or more

**3.The situation of consuming chilli sauce**


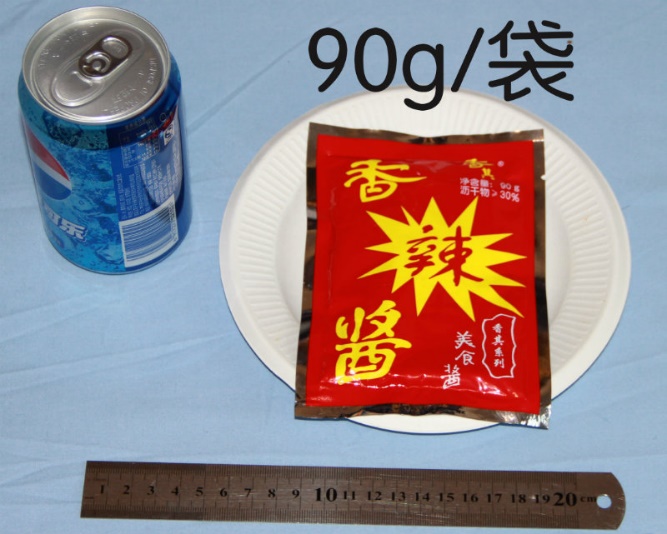
90g

(1) The frequency of consumption is:

□ Never □ 1 to 3 times per month □ Once a week □ 2 to 3 times a week

□ 4 to 5 times a week □ Once a day □ Twice a day □ Three times a day or more

(5)The amount of chilli sauce you consume each time is:

□ Less than or equal to 5 grams □ 10 to 15 grams □ 20 to 25 grams

□ 30 to 35 grams □ 40 to 45 grams □ 50 grams or more

**4.The situation of consuming fermented bean curd**


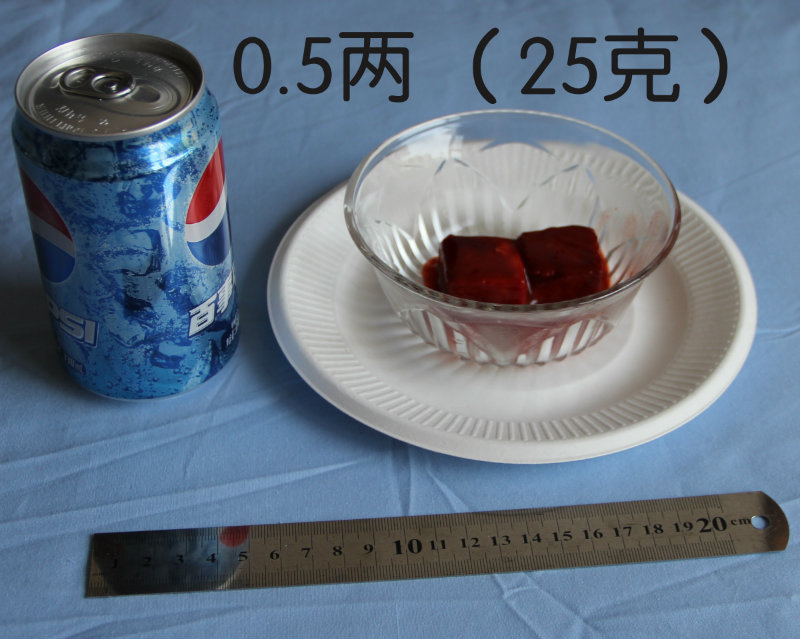
25g

(1) The frequency of consumption is:

□ Never □ 1 to 3 times per month □ Once a week □ 2 to 3 times a week

□ 4 to 5 times a week □ Once a day □ Twice a day □ Three times a day or more

(5)The amount of fermented bean curd you consume each time is:

□ Less than or equal to 5 grams □ 10 to 15 grams □ 20 to 25 grams

□ 30 to 35 grams □ 40 to 45 grams □ 50 grams or more

**5.The situation of consuming pickled cucumber**


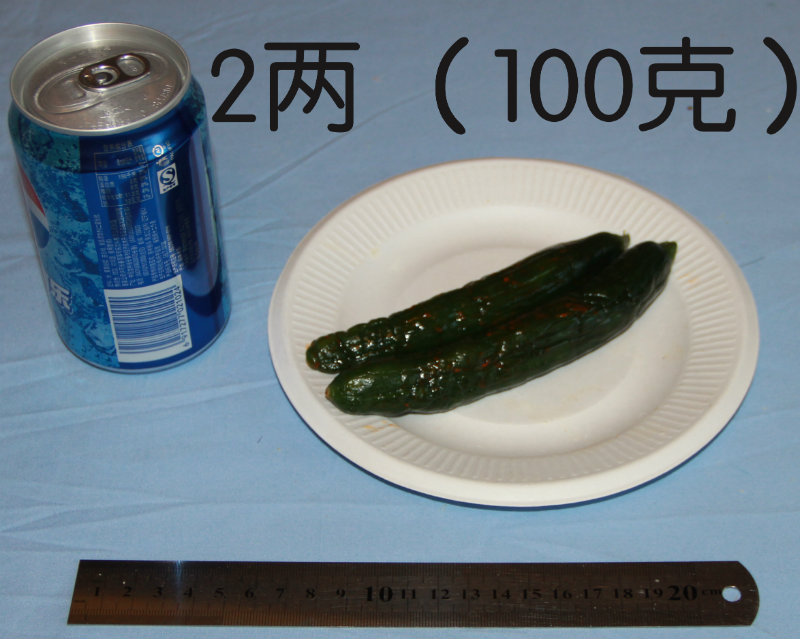
100g

(1) The frequency of consumption is:

□ Never □ 1 to 3 times per month □ Once a week □ 2 to 3 times a week

□ 4 to 5 times a week □ Once a day □ Twice a day □ Three times a day or more

(5)The amount of pickled cucumber you consume each time is:

□ Less than or equal to 5 grams □ 10 to 15 grams □ 20 to 25 grams

□ 30 to 35 grams □ 40 to 45 grams □ 50 grams or more

**6.The situation of consuming dried pickled radish**


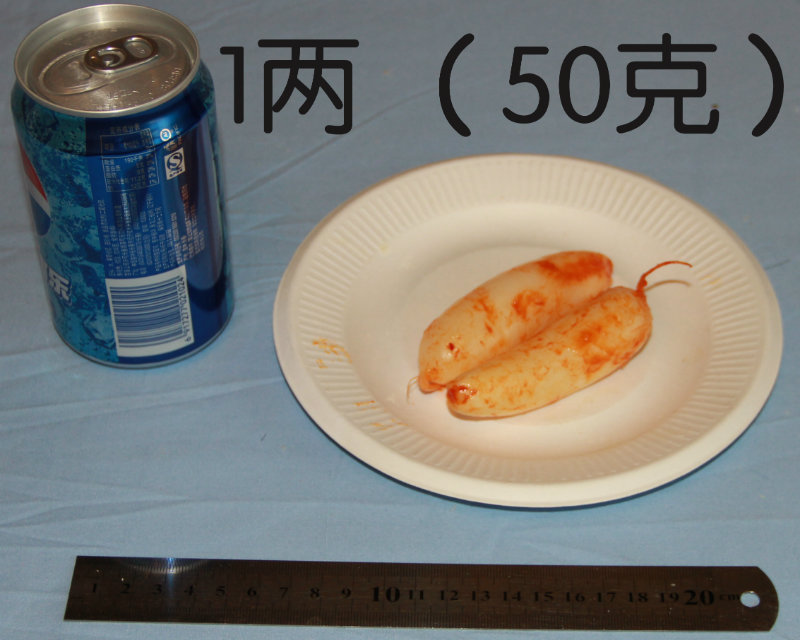
50g

(1) The frequency of consumption is:

□ Never □ 1 to 3 times per month □ Once a week □ 2 to 3 times a week

□ 4 to 5 times a week □ Once a day □ Twice a day □ Three times a day or more

(5)The amount of dried pickled radish you consume each time is:

□ Less than or equal to 5 grams □ 10 to 15 grams □ 20 to 25 grams

□ 30 to 35 grams □ 40 to 45 grams □ 50 grams or more

**7.The situation of consuming pickled garlic**


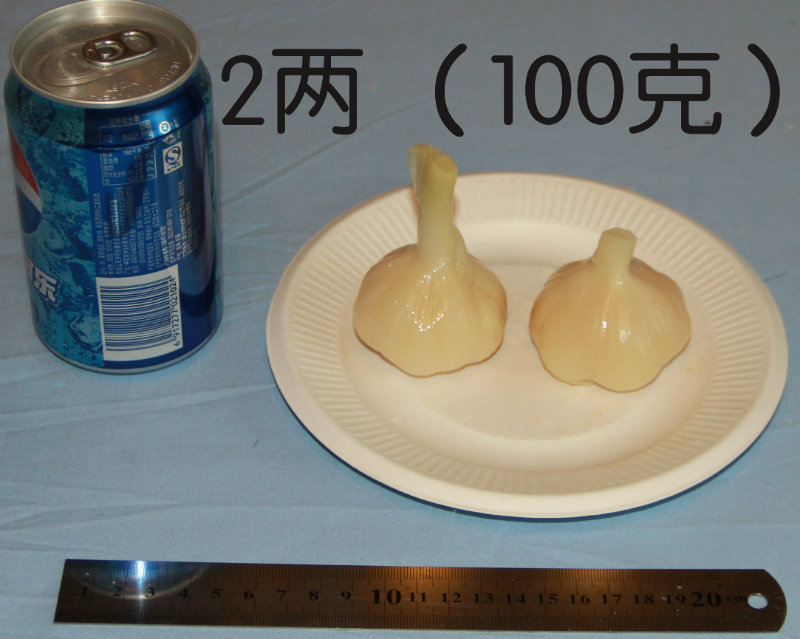
100g

(1) The frequency of consumption is:

□ Never □ 1 to 3 times per month □ Once a week □ 2 to 3 times a week

□ 4 to 5 times a week □ Once a day □ Twice a day □ Three times a day or more

(5)The amount of pickled garlic you consume each time is:

□ Less than or equal to 50 grams □ 100 to 150 grams □ 200 to 250 grams

□ 300 to 350 grams □ 400 to 450 grams □ 500 grams or more

**7.The situation of consuming pickled mustard tuber**


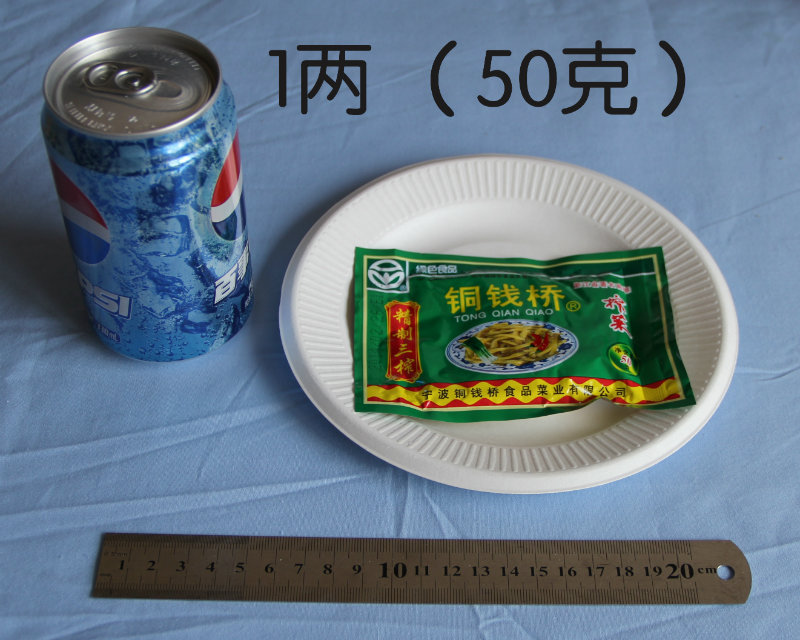
50g

(1) The frequency of consumption is:

□ Never □ 1 to 3 times per month □ Once a week □ 2 to 3 times a week

□ 4 to 5 times a week □ Once a day □ Twice a day □ Three times a day or more

(5)The amount of pickled mustard tuber you consume each time is:

□ Less than or equal to 50 grams □ 100 to 150 grams □ 200 to 250 grams

□ 300 to 350 grams □ 400 to 450 grams □ 500 grams or more

**8.The situation of consuming leek flower**


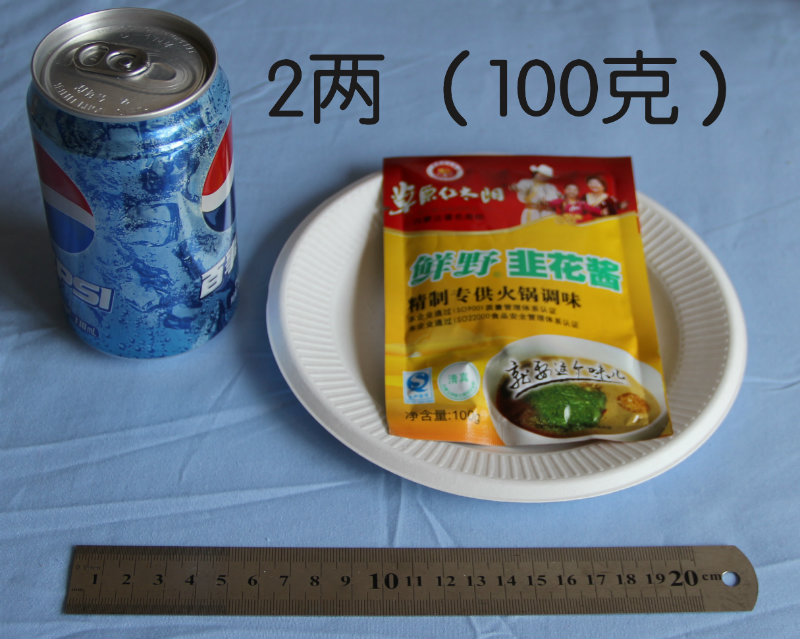


(1) The frequency of consumption is:

□ Never □ 1 to 3 times per month □ Once a week □ 2 to 3 times a week

□ 4 to 5 times a week □ Once a day □ Twice a day □ Three times a day or more

(5)The amount of leek flower you consume each time is:

□ Less than or equal to 50 grams □ 100 to 150 grams □ 200 to 250 grams

□ 300 to 350 grams □ 400 to 450 grams □ 500 grams or more

**9.The situation of consuming mustard**

(1) The frequency of consumption is:

□ Never □ 1 to 3 times per month □ Once a week □ 2 to 3 times a week

□ 4 to 5 times a week □ Once a day □ Twice a day □ Three times a day or more

(5)The amount of mustard you consume each time is:

□ Less than or equal to 5 grams □ 10 to 15 grams □ 20 to 25 grams

□ 30 to 35 grams □ 40 to 45 grams □ 50 grams or more

- **The situation of other**

**1.The situation of consuming silkworm pupae**


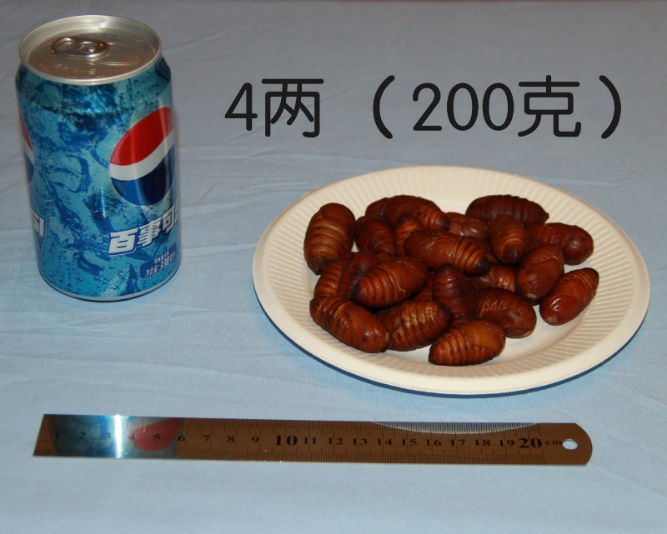
200g

(1) The frequency of consumption is:

□ Never □ 1 to 3 times per month □ Once a week □ 2 to 3 times a week

□ 4 to 5 times a week □ Once a day □ Twice a day □ Three times a day or more

(5)The amount of silkworm pupae you consume each time is:

□ Less than or equal to 50 grams □ 100 to 150 grams □ 200 to 250 grams

□ 300 to 350 grams □ 400 to 450 grams □ 500 grams or more
